# Structural and electronic modulations of lytic polysaccharide monooxygenase (LPMO) upon chitin binding: insights from X-ray spectroscopy

**DOI:** 10.1039/d5sc07620j

**Published:** 2025-11-19

**Authors:** Chris Joseph, Ashish Tamhankar, Ole Golten, Kushal Sengupta, Sergio A. V. Jannuzzi, Morten Sørlie, Liqun Kang, Åsmund K. Røhr, Vincent G. H. Eijsink, Serena DeBeer

**Affiliations:** a Max Planck Institute for Chemical Energy Conversion Stiftstraße 34–36 Mülheim an der Ruhr 45470 Germany serena.debeer@cec.mpg.de; b Faculty of Chemistry, Biotechnology and Food Science, Norwegian University of Life Sciences (NBMU) Ås Norway vincent.eijsink@nmbu.no

## Abstract

Lytic polysaccharide monooxygenases (LPMOs) play a critical role in the depolymerization of recalcitrant polysaccharides, such as chitin, making them of interest in biotechnological applications. These interfacial enzymes are also of great chemical interest because of their unique monocopper catalytic center and their ability to activate high energy C–H bonds. This report investigates the structural and electronic changes at the copper (Cu) site of an LPMO, *Sm*AA10A, upon binding of its chitin substrate, utilizing a suite of spectroscopic and computational methods. Herein, we present the first reported X-ray Absorption (XAS) and Emission (XES) spectroscopic data on substrate-bound LPMO. By comparing the Cu(ii) and Cu(i) states of *Sm*AA10A in both the chitin-bound and unbound states, we provide insights into the structural adjustments facilitating substrate specificity and productive catalytic turnover. Our results indicate a substrate binding-induced conformational change in Cu(i) site geometry and concurrent modulations to the electronic structure, which prime the enzyme for targeted C–H activation with an H_2_O_2_ co-substrate. This work offers an atomistic understanding of interaction dynamics between the LPMO Cu site and the chitin substrate, advancing our knowledge of LPMO functionality and substrate specificity.

## Abstract 2

Characterization of a substrate-bound LPMO using X-ray Absorption (XAS) and Emission (XES) spectroscopies, aided by computational interpretation, reveals key interaction dynamics at the Cu site upon substrate binding.

## Graphical abstract

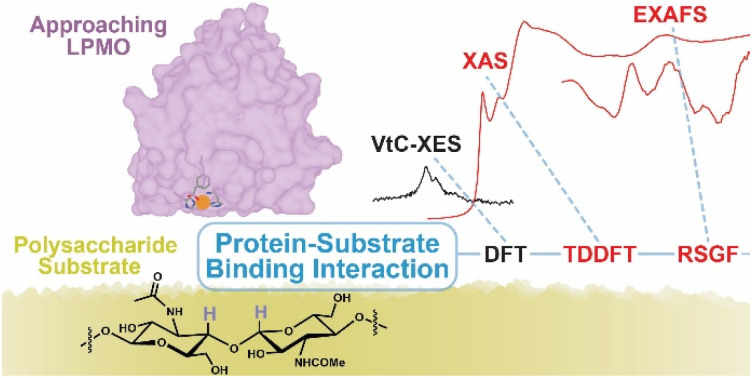

## Introduction

**Fig. 1:**
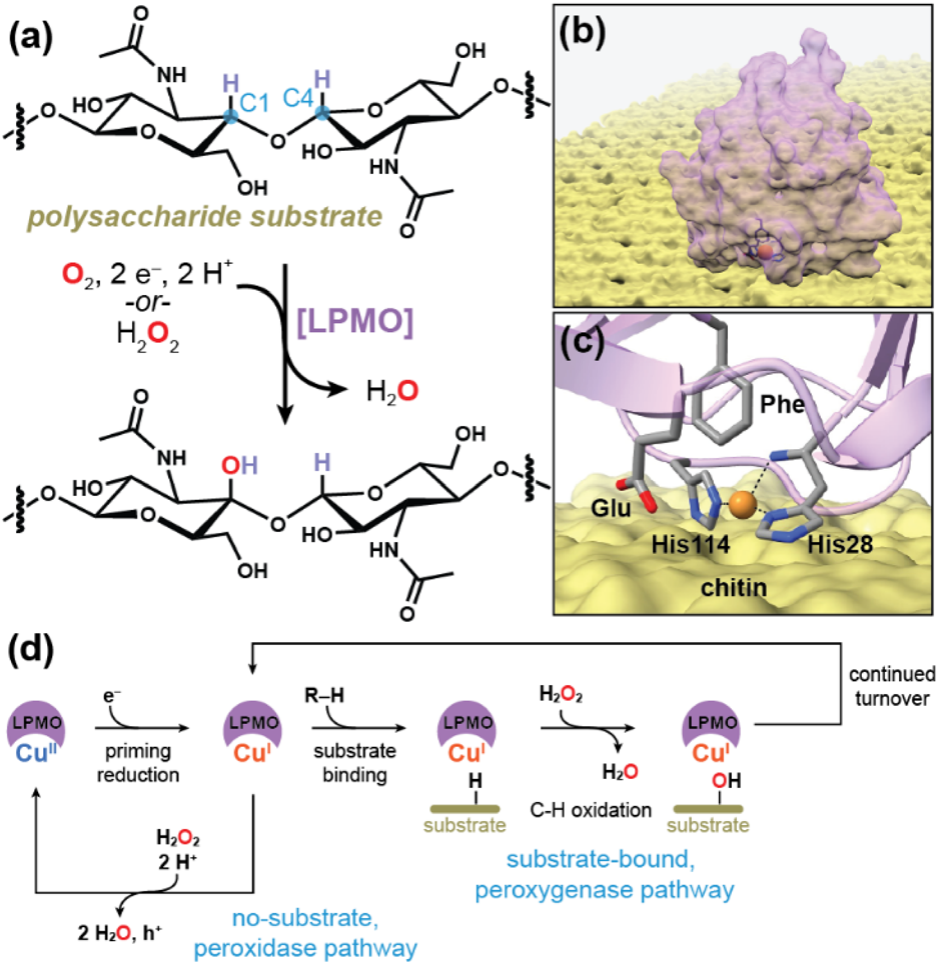
(a) The LPMO metalloenzymes perform C–H oxidation at the glycosidic site of polysaccharides (*e.g.* chitin, cellulose, starch). Notably, *Sm*AA10A displays strong regioselectivity towards hydroxylation at the C1 site of chitin. (b) The copper-centered active site resides at the periphery of the protein, which binds to the surface of the insoluble polysaccharide (*e.g.* chitin, cellulose). (c) The active site of *Sm*AA10A. The black dotted lines indicate coordination to the Cu site. Note that His28 is the N-terminal amino acid following removal of the signal peptide during secretion. However, the original residue numbering scheme has been preserved to remain consistent with published literature on *Sm*AA10A. (d) Overarching mechanism for the productive peroxygenase reaction and the non-productive peroxidase reaction. Note: “h^+^” denotes an electron hole which forms during this pathway and must be diverted away from the active site residues *via* hole hopping paths. Figures (b) and (c) generated using data from ref. 13.

Lytic polysaccharide monooxygenases (LPMOs) comprise a variety of fungal and bacterial carbohydrate-active enzymes responsible for biomass decomposition processes crucial for the re-utilization of bio-organic matter and maintenance of the global carbon cycle.^1–4^ In particular, LPMOs demonstrate a specialized capability for initiating the depolymerization of insoluble and recalcitrant polysaccharides, such as chitin and cellulose, typically by oxidation of the C–H bond located at either the C1 or C4 position (Fig. 1a).^5^ As such, research on LPMOs is driven in part by their potential in biotechnological applications.^3^ More importantly, an improved understanding of LPMOs and their mechanisms may hold the key to designing catalysts for facile C–H activation.

The peripheral, solvent-exposed active site of LPMOs (Fig. 1b) features a single, type-II Cu(ii) center tethered to the enzyme *via* an N-donor-supported, three-coordinate ligation sphere comprised of one conventionally bound His residue (His114) and a unique bidentate utilization of the N-terminal histidine (His28) in a “histidine brace” orientation (Fig. 1c) reminiscent of the coordination sphere supporting the Cu_B_ monocopper site in particulate methane monooxygenase (pMMO).^6–10^ Following an initial Cu(ii) → Cu(i) reduction, LPMOs can activate inert C–H bonds in the presence of O_2_ by selective insertion of a single oxygen atom, which led to the initial characterization of the enzyme as employing a monooxygenase mechanism.^1,11^ However, recent biochemical, computational, and spectroscopic evidence have provided a strong argument for the utilization of a peroxygenase mechanism (Fig. 1d) as the native LPMO mechanism of action.^12–17^ However, excess H_2_O_2_ does promote “off-pathway” peroxidase reactions, especially in the absence of polysaccharide substrate, and the strong oxidative potential intended for glycosidic C–H bond activation may then lead to enzyme damage through oxidation of the damage-susceptible histidine residues that ligate the Cu site.^12,18,19^ In the event of such substrate-free turnover, the LPMO attempts to divert the oxidative hole away from the active site residues through one or more hole hopping paths.^19–24^ Nevertheless, enzyme inactivation does occur, leading the decreased turnover efficiencies over time.^25,26^ Recent kinetic and computational evidence, however, has suggested that binding of LPMO to its polysaccharide substrate increases the kinetic barrier to proceeding onto the unproductive turnover pathway, therefore ensuring that the oxidative potential of the proposed copper-oxyl intermediate is directed towards activation of the glycosidic C–H bond.^18,27^ As such, an understanding of how the LPMO steers itself towards the “productive” turnover pathway necessitates an exploration of the changes effected at the Cu site upon substrate binding.

Several investigations have already begun to interrogate the protein for structural insight relevant to substrate binding.^28–34^ While these and other studies have highlighted the functional roles of various protein residues in substrate binding, few reports have focused on the effects of binding on the Cu site itself. A notable exception can be found in the report by Bissaro *et al.* (2018), in which combined utilization of EPR spectroscopy and QM/MM computational methods described changes at the Cu(ii) site corresponding to chitin binding.^31^ Nevertheless, it is the 1-electron reduced Cu(i) site which is primed for catalytic turnover, and data suggest that the reduced LPMO has a higher affinity for the substrate compared to the Cu(ii) state.^35,36^ Therefore, an atomistic understanding of the changes effected at the Cu(i) site upon substrate binding would serve to inform the molecular attributes which ensure that the LPMO does in fact proceed into the productive glycosidic C–H activating turnover pathway and avoid the undesirable and unproductive hole-hopping or self-destructive reaction pathways. Notably, Tandrup *et al.* (2022) crystallographically characterized oligosaccharide-bound *Ls*AA9A, which had been photoreduced under the exposure to the X-ray beam to induce a tentative Cu(i) state.^33^ As of this writing, however, no published investigation relating experimental characterization of the primed, chitin-bound Cu(i) species has been reported.

In this study, we provide the first reported X-ray spectroscopic study of substrate-bound LPMO to characterize the Cu(ii) and Cu(i) congeners of *Sm*AA10A in both the chitin-free and chitin-bound states, hereafter denoted with following monikers: (1) Cu(ii)-*Sm*AA10A, (2) Cu(ii)-*Sm*AA10A·chitin, (3) Cu(i)-*Sm*AA10A, and (4) Cu(i)-*Sm*AA10A·chitin. X-ray spectroscopic methods present a notable advantage over conventional inorganic spectroscopy techniques (*e.g.*, UV-vis, MCD), which are often incompatible with heterogenous, chitinous samples. In the particular case of the closed-shell d^10^ Cu(i)-containing reductively primed state, X-ray spectroscopic methods are uniquely suited for characterizing the EPR- and optically-silent Cu center. Herein, we utilize a combination of Cu K-edge X-ray absorption spectroscopy (XAS) and extended X-ray absorption fine structure (EXAFS) to experimentally assess the changes that occur in both the Cu(i) and the Cu(ii) congeners in the presence and absence of substrate. Due to the relevance of the Cu(i) site to the catalytic cycle, the electronic structures of the Cu(i) states were further investigated using Valence-to-Core X-ray Emission Spectroscopy (VtC-XES) to characterize the filled valence shell electronic structure of Cu(i)-*Sm*AA10A and Cu(i)-*Sm*AA10A·chitin. The electronic and geometric information provided by X-ray spectroscopy—in conjunction with the interpretive aid enabled by computational chemistry — provides an avenue for understanding changes to the Cu(ii) and Cu(i) sites following chitin binding to the LPMO. Importantly, understanding how these changes prime the Cu site to direct the oxidative potential of H_2_O_2_ for productive C–H activating turnover, rather than off-pathway oxidative damage, serves as a key step towards unravelling the catalytic mechanism of LPMOs.

## Experimental procedures

Experimental procedures detailing the expression & purification of *Sm*AA10A and parameters for EPR and UV-vis spectroscopy are provided in the SI, pages S3 and S4.

### X-ray sample preparation

#### Cu(ii)-*Sm*AA10A

An aliquot of apo-*Sm*AA10A at 2 mM in 50 mM MES, pH 6.0, was incubated with 50 molar equiv. of CuSO_4_ for 30 min at 4 °C, yielding a light-blue solution containing Cu(ii)-*Sm*AA10A. Excess CuSO_4_ was removed by several repeated cycles of spin filtration and dilution with buffer, and the final protein concentration was adjusted to ∼2 mM. A solution of saturated glucose in buffer was added (30% v/v) as a glassing agent, and the final protein concentration was determined by UV-vis (*λ*_280_ = 35 200 M^−1^ cm^−1^) to be 2.4 mM. The sample solution was transferred into Delrin cells with Kapton tape windows, followed by freezing and storage in liquid nitrogen until measurement.

#### Chitin-bound Cu(ii)-*Sm*AA10A

In order to generate Cu(ii)-*Sm*AA10A·chitin, several incubation conditions were used, resulting in a series of chitin-bound LPMO samples with various distributions of Cu(ii)-*Sm*AA10A·chitin*versus*Cu(ii)-*Sm*AA10A (ranging 33–85% chitin-bound LPMO). For each sample, an 80 mg portion of chitin was weighed into a 2 mL Eppendorf tube. For samples for which the chitin was pre-hydrated before incubation, 300 µL of buffer (50 mM MES, pH 6.0) was added, the tubes centrifuged at 9000 rpm for 60 s and allowed to sit at 4 °C for 10 minutes. Protein was drawn from a stock solution of Cu(ii)-*Sm*AA10A and adjusted to the appropriate concentration before being delivered into the chitin-containing tube (see Table S1 for incubation conditions). The mixture was incubated for two hours in an Eppendorf Thermomixer comfort thermomixer at 800 rpm at the target temperature. The mixture was then centrifuged, the supernatant was removed, and the remaining slurry was transferred into Delrin cells and sealed with Kapton tape windows, followed by freezing and storage in liquid nitrogen until measurement. The X-band EPR spectrum of each cell was measured to assess sample purity.

#### Cu(i)-*Sm*AA10A

Samples of Cu(i)-*Sm*AA10A were prepared in 50 mM MES pH 6.0 by reduction of the Cu(ii) congener by 50 molar equiv. ascorbic acid under anaerobic conditions. We wished to avoid S-containing reductants such as *L*-Cys and dithionite, as these have been shown to act non-innocently in affecting the reactivity of LPMOs, as well as hydrogenases.^37,38^ Ascorbic acid has strong precedence as a reductant in LPMO biochemical studies and was therefore selected for this study. The reduced protein was isolated by gel filtration (GE Healthcare illustra NAP-5 column), and saturated glucose solution (30% v/v) was added as a glassing agent. The final protein concentration was determined by UV-vis (*λ*_280_ = 35 200 M^−1^ cm^−1^) to be 2.0 mM. The sample solution was transferred into Delrin cells with Kapton tape windows, followed by anaerobic freezing and storage in liquid nitrogen until measurement. Prior to X-ray spectroscopic experiments, samples were confirmed to be diamagnetic by EPR, indicating full reduction (Fig. S1).

#### Chitin-bound Cu(i)-*Sm*AA10A

An 80 mg portion of chitin was weighed into a 2 mL Eppendorf tube and transferred to an anerobic chamber. A 400 µL aliquot of 2.2 mM Cu(i)-*Sm*AA10A (prepared as described above and used immediately) was added into the chitin-containing tube, and the mixture was incubated for two hours in an Eppendorf Thermomixer comfort thermomixer set to 40 °C temperature and 800 rpm mixing speed. The resulting suspension was then centrifuged, the supernatant removed, and the remaining slurry transferred into Delrin cells and sealed with Kapton tape windows, followed by anaerobic freezing and storage in liquid nitrogen until measurement. Prior to X-ray spectroscopic experiments, sample cells were checked by EPR to be diamagnetic to confirm full reduction (Fig. S1).

### Partial fluorescence yield-detected (PFY) X-ray absorption spectroscopy

#### Data collection

Cu K-edge X-ray absorption data were measured at the I20-scanning beamline at Diamond Light Source (DLS; Oxfordshire, UK) (3 GeV, 300 mA)^39,40^ utilizing scan parameter we previously reported for measurements on Cu(i)-*Sm*AA10A.^41^ A four-bounce monochromator equipped with Si(111) crystals was utilized for upstream energy selection, and rhodium-coated mirrors were used for harmonic rejection, providing an unattenuated flux of ∼1 × 10^12^ photons sec^−1^ at the sample position. The X-ray beam was focused to an approximate beam spot size of 0.3 × 0.4 mm^2^ (*v* × *h*; full width at half maximum (FWHM)). During measurements, the sample temperature was maintained at 10 K using a top loading exchange gas pulse tube He cryostat to minimize photodamage. In general, the data were collected by scanning the incident energy from 8859 to 9640 eV and calibrated by simultaneous measurement of a Cu foil and setting the first inflection point to 8980.3 eV during data processing. For the Cu(ii)-*Sm*AA10A + β-chitin sample composed of 60% chitin-bound species, the scan range was limited to short sweeps from 8961 to 9026 eV. Fluorescence data from the sample were recorded using a 64-element monolithic Ge detector, with read-out performed by the Xspress4 digital pulse processor. In order to assess beam-induced damage of Cu(ii)-containing biological samples,^10,33,42^ Cu K-edge spectroscopic data were collected on Cu(ii)-containing samples using a significantly attenuated X-ray beam following detailed damage assessment studies prior to data collection. Therefore, a series of short, low-resolution scans at the near-edge region were collected to identify the resistance of the sample to X-ray-induced photodamage (Fig. S3). Successful acquisition of undamaged spectra was achieved by attenuation of the incident beam (0.27% of total flux for all Cu(ii) samples and 85% of the beam for Cu(i) samples) and translation to a fresh sample spot between each scan.

#### Background subtraction and normalization

Initial data processing was performed in the Athena module of the Demeter software package.^43^ The energy axis of the PFY-XAS data were first calibrated such that the first inflection point of the Cu foil was set to 8980.3 eV. Data were truncated as needed, and background subtraction and normalization of the data were performed using a linear regression for the pre-edge region and a cubic polynomial regression for the post-edge region. Data were splined along the post-edge range using an R-background of 1.0 and *k*-weight of 3. For the chitin-containing Cu(ii)-*Sm*AA10A samples exhibiting various extents of chitin-binding, contributions of unbound LPMO to the spectra were subtracted from the final spectra using Larch Larix, and the resulting spectrum was again normalized using the procedure described above (Fig. S4).^44^ From these data, the treated 33% chitin-bound data provided the most representative Cu K-edge spectrum for Cu(ii)-*Sm*AA10A·chitin (Fig. S5). Further remarks on this determination can be found in the SI, page S13.

#### FEFF fitting of FT-EXAFS

The EXAFS regions of each spectrum were *k*^3^-weighted to enhance the impact of the high-*k* data. From the available data for Cu(ii)-*Sm*AA10A·chitin, it was determined that the data from the 75% bound sample provided the most superior data quality (Fig. S6). Therefore, the data collected from the 75% chitin-bound sample was deemed most suitable for data modeling Cu(ii)-*Sm*AA10A·chitin, and this data was therefore used for further processing. We note that EXAFS data is not as sensitive to mild photodamage as the near-edge spectra, particularly in the case of Cu(ii).^10,45^ EXAFS modeling and fitting were performed by using the Artemis module within Demeter.^43^ The *k*^3^-weighted EXAFS were Fourier transformed over the following Hanning-windowed *k*-ranges, giving the Δ*R* resolution delineated in parenthesis: (a) Cu(ii)-*Sm*AA10A*k* = 2–12.5 Å^−1^ (Δ*R* = 0.150 Å), (b) Cu(ii)-*Sm*AA10A·chitin*k* = 2–11 Å^−1^ (Δ*R* = 0.174 Å), (c) Cu(i)-*Sm*AA10A*k* = 2–12 Å^−1^ (Δ*R* = 0.157 Å, reported in ref. 41), and (d) Cu(i)-*Sm*AA10A·chitin*k* = 2–11.1 Å^−1^ (Δ*R* = 0.172 Å). Truncating the *k*-ranges to 11 Å^−1^ did not dramatically alter fits. The data for all chitin-containing samples exhibited a Hf L_3_ edge at ∼9560 eV. It has been previously documented that chitin tends to sequester heavy metals, including hafnium, from the environment.^46–48^ Therefore, the data were truncated after ∼9500 eV (*k* = ∼11.45 Å^−1^), and the Fourier transform was performed over shortened *k*-windows relative to their non-substrate bound counterparts. Scattering paths were calculated using FEFF6 and fit to the FT data over a range of *R* = 1.0–4.0 Å (non-phase shift corrected). Similar scattering paths were grouped together as degenerate paths when the path lengths were within the resolution of the Fourier transform. We note that EXAFS cannot reliably distinguish between N and O backscattering atoms due to their similar scattering potentials. Therefore, some paths are tabulated as Cu–N/O and interpreted to have contributions from the amine, imidazoles, and water ligands. A single *E*_0_ variable was applied for all paths in a given fit. The amplitude reduction factor (S_0_^2^) was fixed at 0.9 for all paths. An initial fit was performed using FEFF-calculated paths generated from the geometry optimized structure. Atoms used for the FEFF calculation are shown in Fig. S7. For each multiple-scattering path, *σ*^2^ was defined by multiplying the *σ*^2^ of the single-scattering path of the more proximal scatterer by a factor of 1.5, in order to avoid the introduction of additional variables into the fit. Path distances (*R*) were then iteratively fit to approach a final fit in which all variables *R*, *σ*^2^, and *E*_0_ could be freely floated. Information about selected fits can be found in Tables S4, S5, S9, S10 and Fig. S8, S9, S12. Additional information on the isolation and fitting of the chitin-bound data can be found in the SI, pages S21–S25.

### Kβ valence-to-core X-ray emission spectroscopy (VtC-XES)

#### Data collection

Cu Kβ XES data were collected at beamline 15-2 at the Stanford Synchrotron Radiation Lightsource (SSRL, Menlo Park, California, USA) operating in top-up mode with a beam current ranging from 500 to 493 mA. The incident X-ray energy was selected using a Si(111) double crystal monochromator, and calibration of the monochromator energy was performed by simultaneous collection of a Cu foil spectrum, which was calibrated to 8980.3 eV during data processing. Vertical and horizontal focusing was accomplished using two rhodium coated Kirkpatrick–Baez (KB) mirrors, and the X-ray beam was focused to an approximate spot size of 63 × 525 µm^2^ (*v* × *h*, full width at half maximum (FWHM)), providing, nominally, an unattenuated flux of ∼3 × 10^13^ photons sec^−1^ at the sample position. Fluctuations in the beam intensity were monitored using a He-filled ionization chamber (I_0_) positioned before the sample. To minimize photodamage, the sample temperature was maintained at approximately 10 K using a top-loading He cryostat. Detection was achieved using a Johann spectrometer configuration equipped with seven Si(553) analyzer crystals arranged along a 1 m Rowland circle focused at a Hitachi Vortex silicon drift detector (SDD). Energy and intensity calibration of the spectrometer was done using a series of 17 elastic peak scans with set incident energies spanning from 8894 to 8984 eV and applying quadratically-modeled energy shift and intensity scaling factor. An additional Hitachi Vortex SDD was positioned just above the analyzer crystals and windowed to ±200 eV around the Cu Kα energy, serving as a PFY detector during the experiment. Prior to data collection, the detected emission energy was set to the mainline maximum of each sample, and a series of short HERFD scans at the near-edge region were collected to assess sample vulnerability to X-ray-induced photodamage by monitoring the rate of decreasing intensity of the 1s → 4p rising edge feature at 8983 eV, and the beam was attenuated to ∼40% of total flux for data collection. The Kβ mainline (ML) data were collected by setting the incident energy to 9750 eV and scanning the emission energy from 8880 to 8939 eV, and valence-to-core (VtC) data were collected by scanning from 8920 to 9002 eV.

#### Data processing

Data processing and visualization were performed using a Python (3.11.5) environment equipped with the scipy (1.13.0) and lmfit (1.2.2) modules.^49,50^ The averaged ML and VtC data were stitched together by performing a least-squares minimization fitting of the VtC data to the ML data in the 8920 to 8939 eV range using *y* = *mx* + *b* as the fitting function, where *y* is the ML data intensity and *x* is the VtC data intensity. Energy calibration and intensity corrections were applied as determined by the elastic peak scans collected during the experiment. The emission energy axis was calibrated to agree with a Cu foil first inflection point of 8980.3 eV, providing ML maxima centered at 8904.9 eV (Fig. S13). The data were normalized by setting the minimum to 0, dividing by the total integrated area over the entire ML + VtC scan range, and scaling the result by 1000. The contribution of the mainline to the background of the VtC region, for which features are approximately two orders of magnitude less intense than in the mainline, was approximated using pseudo-Voigt peaks (Fig. S14) and subtracted from the data. Standard Error was determined and propagated as described in our recent work.^51^ Peak-fitting analysis was applied to spline-smoothed (*s* = 0.05) VtC spectra (Fig. S15).

### Computational methods

All Density Functional Theory (DFT) and Time-Dependent Density Functional Theory (TDDFT) calculations were performed using ORCA 5.0.4.^52–56^ Localized orbital overlaps and excited-state hole–electron Hirshfeld populations were calculated using Multiwfn.^57,58^ Additional computational method descriptions detailing the preparation of the *Sm*AA10A small cluster models and Histidine Brace toy complexes, calculations of EXAFS data using FEFF10 code, and binding energy calculations are provided in the SI, pages S4–S6.

#### TDDFT calculation of X-ray absorption spectra

X-ray absorption spectra were calculated as previously reported by our group for *Sm*AA10A with TDDFT, using established methodological protocols with the CAM-B3LYP functional and the Tamm–Dancoff approximation.^41,59–64^ Scalar relativistic effects were included using ZORA with ZORA-def2-TZVP basis sets.^65,66^ The Autoaux procedure was used to assign an auxiliary basis for Coulomb fitting.^67^ The chain of spheres approximation (RIJCOSX) was employed for the RI approximation to the Coulomb integrals in addition to the auxiliary basis.^68^ These calculations were conducted using the implicit solvation model CPCM with default settings.^69^ The SCF convergence criteria were set with “TightSCF” and “SlowConv” keywords. Unrestricted Kohn Sham formalism was employed for open shell systems (*i.e.* the Cu(ii)-containing structures). A sample input for TDDFT is provided in the SI, page S7. The absorption spectra were plotted using the orca_mapspc utility tool with a broadening of 2.40 eV with a Gaussian line fitting with a constant energy shift of −4.65 eV applied for comparison against the experimental data. The transition attributions were based on natural transition orbitals^70^ generated using the orca_plot utility tool and analyzed in PyMOL. The orientation of the histidine brace in all cluster models is such that the *y* axis was set along Cu–N^*δ*1^_His28_ bond involving the His28 imidazole ring, the *z* axis orthogonal to the Cu–N^*δ*1^_His28_–N_term_ plane, leaving the *x* axis along Cu–N_term_ bond of the His28 primary amine.

#### DFT calculation of valence-to-core emission spectra

Cu Kβ X-ray emission spectra were calculated similar to as described in Lee *et al.*^71^ using the BP86 functional,^72,73^ incorporating scalar relativistic effects using ZORA^65^ with ZORA def2-TZVPP basis set for Cu and ZORA-def2-TZVP for all other atoms.^66^ The spin orbit coupling was included. The SCF convergence criteria were set to “TightSCF”. The use of a pure functional is to avoid Hartree–Fock exchange in the DFT potential, so that the orbital energy difference is a well-defined approximation to the state energy difference.^71^ A uniformly constant energy shift of +33 eV was applied for comparison against the experimental data. The calculated emission spectra were plotted with a Gaussian line broadening of 2.40 eV. The orientation of the histidine brace in all cluster models is the same as specified above for the calculated X-ray absorption spectra.

#### Reactivity calculations

The Cu(i)-*Sm*AA10A·H_2_O_2_ models were optimized with B3LYP functional^62,63^ (to account for exact Hartree Fock exchange) using def2-TZVPP basis set for Cu, while using def2-SVP basis set for all other atoms in the cluster models.^74^ Similar to in our previous report,^17^ the chain of spheres approximation (RIJCOSX)^68^ were used for the RI (resolution of identity) approximation to the Coulomb integrals. The calculations were performed using water implicit solvation as per the control of the Conductor-like Polarizable Continuum Model (CPCM)^69,75^ approach and using atom-pairwise dispersion correction based on tight binding partial charges (D4).^76,77^ The optimization convergence criteria were set to “TightOpt”, while the SCF convergence criteria were set with “TightSCF” keyword. The default integration grid (Def2grid) was employed. Thereafter, the optimized Cu(i)-*Sm*AA10A·H_2_O_2_ models were subjected to relaxed surface scan unrestricted calculations varying the O–O bond distance going from 1.2 Å to 3.0 Å in 0.1 Å steps in the low spin state and in high spin state. The local minima, transition state, and global minima were further optimized similar to our protocol in ref. 17, which considers the relativistic effects using ZORA, with ZORA-def2-TZVPP basis set for Cu, while using ZORA-def2-TZVP for the rest of the atoms in the cluster models.^17,65,66^ The RI approximation to Coulomb integrals was calculated using SARC/J basis set.^66^ The optimizations were performed using TightOpt settings. For frequency calculations the criteria were increased to match the TightOpt setting. The optimized structures were then used as input for partial Hessian calculations with the same protocol to avoid the influence of cluster model constraint artifacts on frequency calculations. The thermodynamic functions were calculated at 4 °C.

## Results

### Effects of incubation conditions on chitin binding extents

**Fig. 2:**
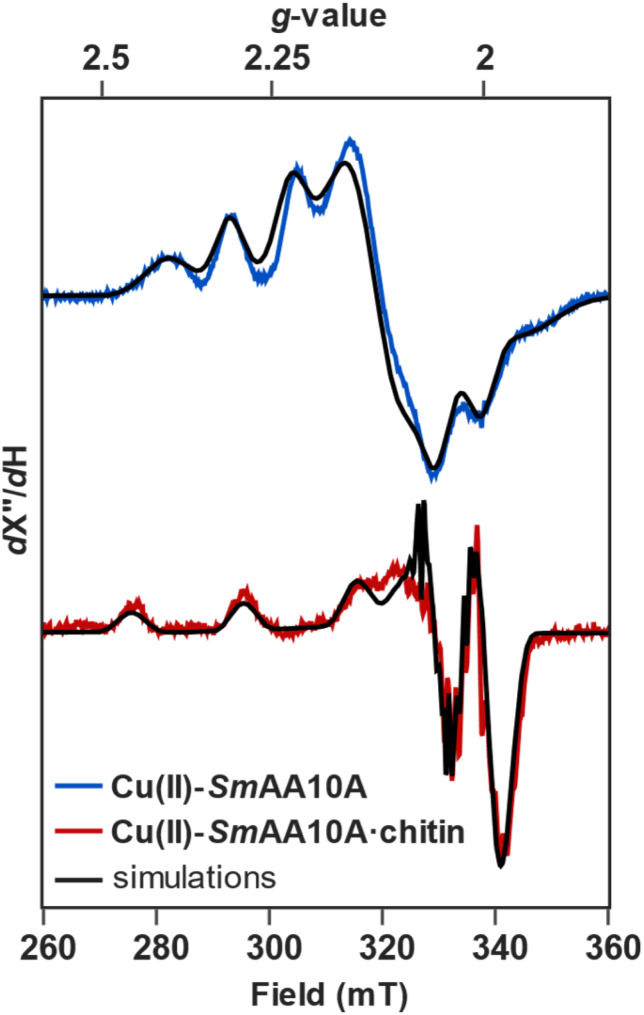
CW X-band EPR of Cu(ii)-*Sm*AA10A (blue) and Cu(ii)-*Sm*AA10A·chitin (red). Simulations are shown in black. Experimental parameters are delineated in Table S2, and simulated spin Hamiltonian parameters are delineated in Table S3. Data for the chitin-bound spectrum is derived from the 33% bound sample, following subtraction of the solution-state component (Fig. S2).

The incubation of Cu(ii)-*Sm*AA10A with chitin yielded X-band EPR spectrum, which presented as a mixture of the protein in the chitin-free (frozen solution-phase) and chitin-bound states, is shown in Fig. S2. Spectral subtraction of the free Cu(ii)-*Sm*AA10A contributions to the spectrum yielded a difference spectrum with simulated spin Hamiltonian parameters (Table S3) similar to those previously reported.^31^ In the solution state, Cu(ii)-*Sm*AA10A produces an EPR spectrum (Fig. 2) characteristic of a Type-II Cu site, with a *g*_*z*_ of 2.263 and rhombic splitting of the *g*_*x*_ and *g*_*y*_ at 2.023 and 2.120, respectively. Upon chitin binding, the *g*_*z*_ exhibits an upfield shift to 2.210, and the *g*_*x*_ and *g*_*y*_ converge towards a more axial spectrum at 2.045 and 2.065, respectively.

In order to generate high-purity samples of chitin-bound *Sm*AA10A for X-ray experiments, we performed an initial optimization study monitored by EPR spectroscopy. To our knowledge, an EPR-monitored substrate-binding optimization study has not thus far been reported for any LPMO. Upon iterating through various incubation parameters (temperature, protein concentration, amount of protein added, Table S1), it was determined that temperature had a significant effect on binding, and the best samples were produced when incubating at 40 °C, which is still well below the apparent melting temperature (*i.e.*, denaturation midpoint) of *Sm*AA10A.^24^

### X-ray absorption spectroscopy

**Fig. 3:**
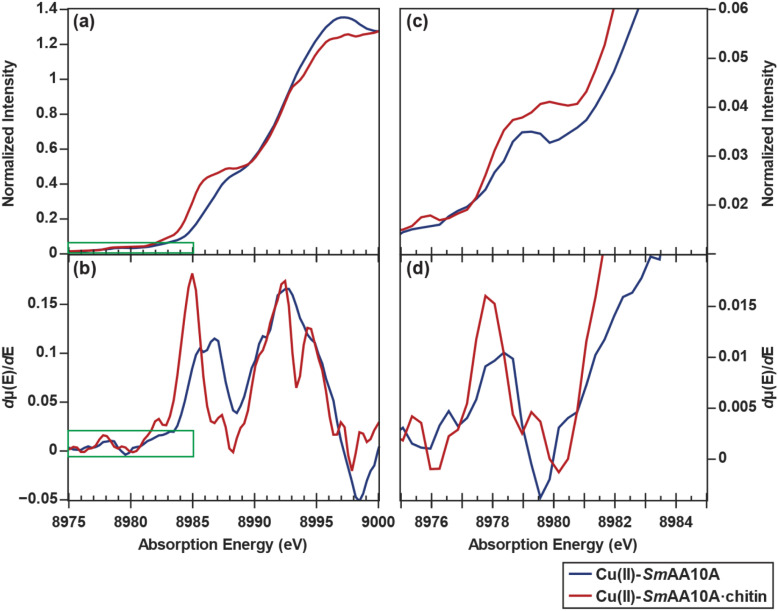
Cu K-edge XAS data for Cu(ii)-*Sm*AA10A (blue) and Cu(ii)-*Sm*AA10A·chitin following subtraction of spectral contributions from the unbound species (red). Data for the chitin-bound spectrum is derived from the 33% bound sample (following subtraction). Shown here are (a) the normalized edge region and (b) the first derivative of the edge region followed by (c) the zoomed-in normalized pre-edge region and (d) its first derivative spectrum. The zoom windows are indicated by the green rectangles in (a) and (b). A three-point smoothing filter has been applied to emphasize signal shape of the pre-edge. The unsmoothed data is provided in the SI (Fig. S5).

Considering the limitations enforced by the chitin matrix on available spectroscopic methods, XAS was deemed the most suitable method for characterizing the Cu site in both the presence and absence of substrate binding and in different oxidation states. Partial fluorescence yield (PFY) XAS data were obtained for the Cu(ii) samples with 33–85% chitin-bound Cu(ii)-*Sm*AA10A·chitin. By measuring a range of samples with different percentages of the LPMO bound to chitin, the features associated with chitin binding could be quantitatively identified. Subtraction of the spectral contribution from unbound Cu(ii)-*Sm*AA10A results in a convergence of all spectra towards a representative spectrum for Cu(ii)-*Sm*AA10A·chitin (Fig. S4). Only the feature at 8983.5 eV (attributed to X-ray induced photoreduction) varies significantly between samples (see remarks in SI, page S13). The Cu K-edge spectrum of Cu(ii)-*Sm*AA10A (Fig. 3) is similar to PFY-XAS data reported on substrate-free AA9 Cu(ii) LPMOs,^17,78,79^ exhibiting a weak, dipole forbidden 1s → 3d pre-edge transition at 8979.2 eV and rising edge feature at 8988.6 eV. Examination of the first derivative spectra for the solution and chitin-bound states reveals a shift in the rising edge feature from 8988.6 eV (in Cu(ii)-*Sm*AA10A) to 8986.4 eV in Cu(ii)-*Sm*AA10A·chitin (a 2.2 eV downward shift). Inspection of the white line maximum at 8997.5 eV shows a decrease in intensity from 1.34 to 1.26 (normalized *µ*(*E*)). Moreover, an increase in the energy of the Cu K-edge is correlated with the emergence of a shoulder feature on the rising edge at ∼8993 eV. Lastly, inspection of the pre-edge feature and the derivative spectra reveals an earlier onset of the feature in the chitin-bound state, as well as an overall broadening and shift from 8979.2 eV to 8979.8 eV (Fig. 3c and d). Interestingly, while examination of the derivative spectrum confirms only negligible X-ray induced photoreduction at ∼8983 eV in Cu(ii)-*Sm*AA10A, the substrate-bound Cu(ii)-*Sm*AA10A·chitin species exhibited increased susceptibility to photoreduction (Fig. S3 and S4), which may have implications for the reduction mechanism (see Discussion).

**Fig. 4:**
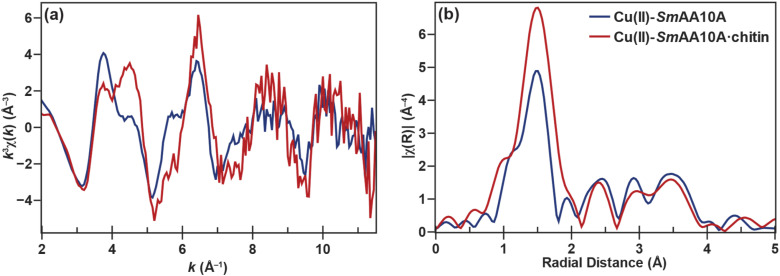
(a) *k*^3^-weighted EXAFS and (b) FT-EXAFS of *Sm*AA10A-Cu(ii) (blue) and *Sm*AA·10A-Cu(ii)·chitin (red). Data for the chitin-bound spectrum is derived from the 75% chitin-bound sample following subtraction.

The *k*^3^-weighted extended X-ray absorption fine structure (EXAFS) data of Cu(ii)-*Sm*AA10A (Fig. 4a) is consistent with previously published data for various LPMOs,^17,78,80^ and the Fourier transform (FT) EXAFS (Fig. 4b) can best be simulated using a fitting model comprised of Cu–N scattering paths at 1.98 Å (*N* = 3) and Cu–O scattering paths at 2.13 Å (*N* = 2) (Table S4 and Fig. S8). Qualitative examination at the *k*^3^-weighted EXAFS of Cu(ii)-*Sm*AA10A·chitin shows a lower frequency in the beat pattern of the EXAFS compared to that of the solution-state. This is consistent with the simulation of the FT-EXAFS, which suggests a contracted primary shell of 1.96 Å and decreased coordination number (*N* = 4) (Table S5 and Fig. S9). Furthermore, the fitted *E*_0_ values confirm the approximately +1 eV edge upward shift qualitatively observed in the K-edge data. For brevity, details on the EXAFS fitting results are omitted here and can be found in the SI (pages S17–S25). Overall, the dramatic differences between the XAS data for the Cu(ii) species signifies the occurrence of a dramatic structural change upon chitin binding, which results in decreased coordination number at the copper site, most likely correlating with the loss of a water ligand.

**Fig. 5:**
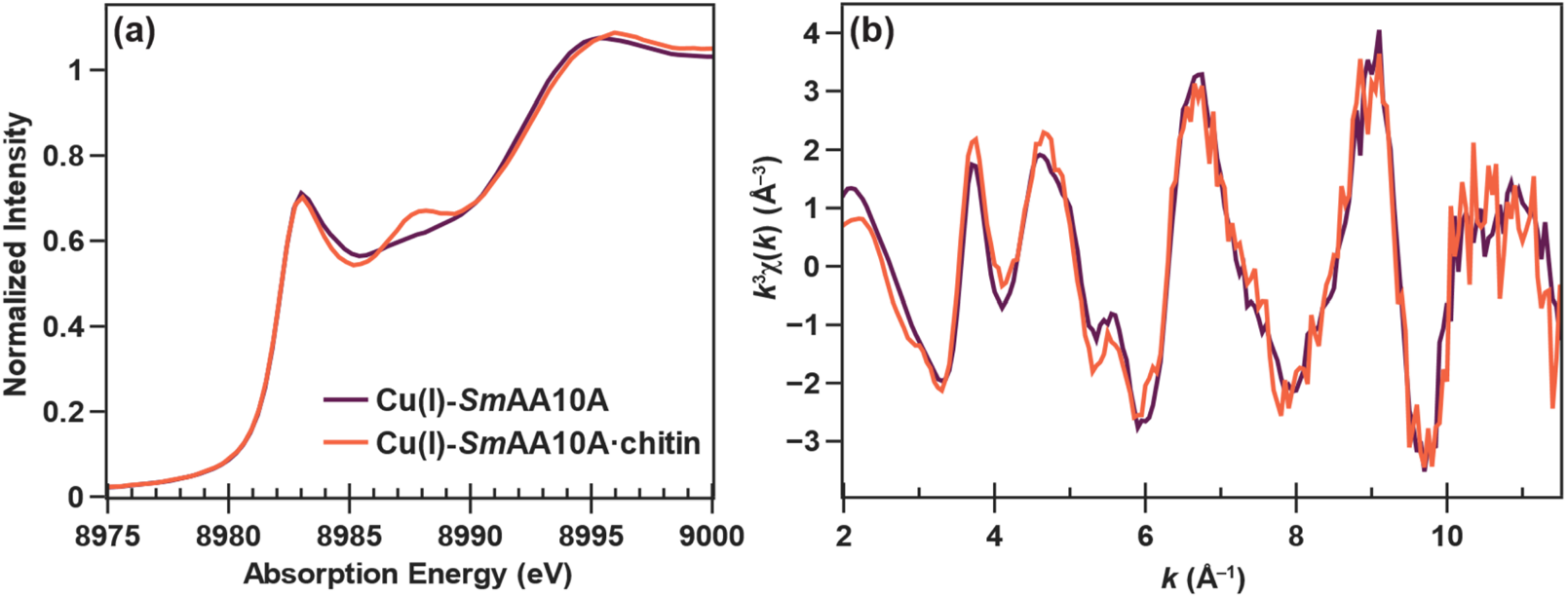
Cu K-edge XAS data for Cu(i)-*Sm*AA10A (purple) and Cu(i)-*Sm*AA10A·chitin (orange). Depicted are (a) the normalized edge region and (b) the *k*^3^-weighted EXAFS data.

The PFY-XAS Cu K-edge spectrum of Cu(i)-*Sm*AA10A (Fig. 5) exhibits a rising edge feature at 8983 eV with a shape and intensity characteristic of a three-coordinate Cu(i) center,^81^ as has been previously observed in Cu(i)-bound AA9 from *T. aurantiacus*^78,79^ and Cu-*Ba*CBM33.^82^ The *k*^3^-weighted EXAFS data confirms this assignment, as fitting of the Fourier transformed data is best modeled with primary Cu–N scattering paths at 1.89 Å (*N* = 2) and a second Cu–N scattering path at 2.21 Å (*N* = 1) (Table S9). Unlike their EPR-active Cu(ii) counterparts, the compositional contribution of Cu(i)-*Sm*AA10A·chitin*versus*Cu(i)-*Sm*AA10A cannot be quantified by EPR in the chitin-incubated Cu(i) sample. Nevertheless, clearly distinguishable featural changes are apparent between the two Cu K-edge spectra (Fig. 5). While the 1s → 4p rising edge feature at 8983 eV remains unshifted and maintains intensity in the chitin-bound data, a decrease in the shoulder intensity at 8985 eV can be observed. More notable is the emergence of a higher-energy feature which maximizes at 8988.1 eV. Additionally, a subtle shift of the edge to higher energy can be observed in the Cu(i)-*Sm*AA10A·chitin data at approximately 8993 eV. Despite the clear changes in the edge spectrum, the *k*^3^-weighted EXAFS data appears to display only minor perturbations overall, when compared to that of Cu(i)-*Sm*AA10A. The fitting model that best simulated the FT-EXAFS of Cu(i)-*Sm*AA10A·chitin, displays negligible changes of Cu–N path distances at 1.90 Å (*N* = 2) and 2.22 Å (*N* = 1) (Table S10 and Fig. S12). Further details regarding the EXAFS fitting results are related in the SI (pages S26 and S27). Taken together, the XAS data on the Cu(i) species suggest that the electronic structure changes indicated in the near-edge data (the appearance of the 8988.1 eV feature) likely do not correspond to dramatic structural changes (*i.e.* bond forming, bond breaking) as seen in the Cu(ii) case. Rather, structural changes at the Cu(i) site are expected to be more subtle, likely conformational.

### Kβ X-ray emission spectroscopy

**Fig. 6:**
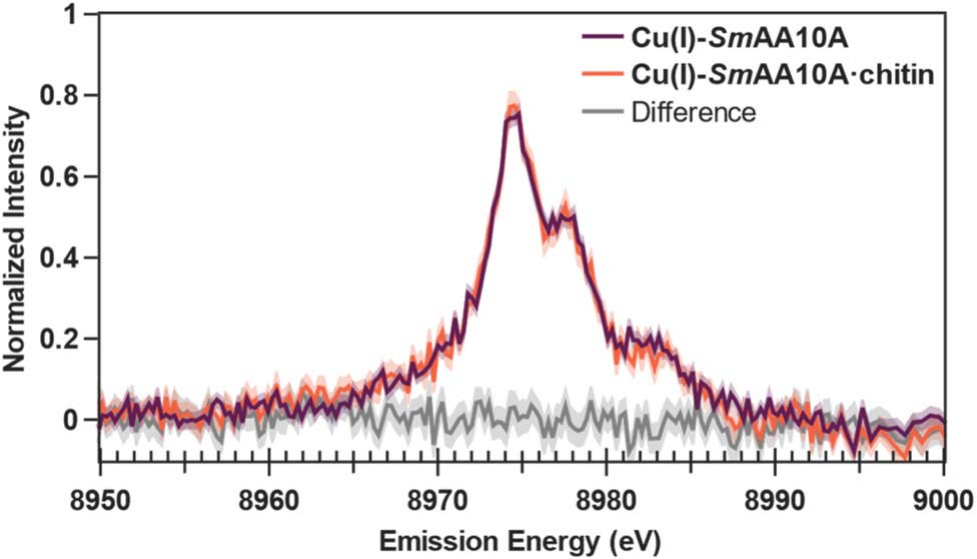
Cu Kβ XES valence-to-core spectra of Cu(i)-*Sm*AA10A (purple) and Cu(i)-*Sm*AA10A·chitin (orange) and the difference spectrum. The standard error of each spectrum is represented by the shaded region.

In contrast to XAS, which probes the electronic structure of the low-lying unoccupied orbitals, Kβ X-ray emission spectroscopy surveys the high-lying occupied orbitals as electrons from these orbitals relax to fill the 1s core–hole generated from ionization by the incident X-ray beam. Often, the Kβ mainline (ML) can serve as a valuable probe in 3d transition metal spectroscopy.^83^ However, it is generally not sensitive to the chemical environment of d^10^ Cu(i) species.^84^ Thus, both Cu(i)-*Sm*AA10A and Cu(i)-*Sm*AA10A·chitin samples afford overlaying ML spectra, with peak maxima at 8904.9 eV and a shoulder at ∼8903 eV (Fig. S13). On the other hand, Kβ valence-to-core (VtC) spectroscopy provides an avenue to probing the valence electronic structure, featuring electron transitions which originate from the ligand *n*p and metal 3d orbitals, providing intensity at the Kβ_2,5_ region of the VtC spectrum.^85^ This method has previously demonstrated utility in His-coordinated Cu(i) proteins, including the first application of the technique to an AA9 LPMO.^86–88^ Subtraction of the mainline contribution to the background of the VtC region yielded the VtC-XES spectra of Cu(i)-*Sm*AA10A and Cu(i)-*Sm*AA10A·chitin (Fig. 6). As illustrated in Fig. 6, the two spectra nearly overlay, and differences cannot be confidently determined outside of the standard error. Peak-fitting analysis of the smoothed VtC spectrum from Cu(i)-*Sm*AA10A could be fit using 4 pseudo-Voigt peaks (Fig. S15): two VtC transition peaks at 8974.5 eV and 8977.9 eV, a higher-energy, low-intensity satellite peak at 8982.7 eV (see Discussion section), and a broad underlying feature centered at 8971.8 eV. The VtC energies of Cu(i)-*Sm*AA10A reflect those previously reported for *Hj*LPMO9A (after accounting for calibration of Cu foil first inflection point to 8980.3 eV).^88^ The same peak fitting analysis could be applied to the Cu(i)-*Sm*AA10A·chitin data, converging to fit VtC peaks centered around 8974.5 eV and 8978.0 eV, respectively (Fig. S15). Overall, these observations indicate that the electronic structure changes observed in the Cu K-edge XAS data are primarily confined to the higher-energy, unoccupied orbitals and do not extend to or significantly affect the electronic structure of the occupied valence orbitals.

### Theoretical calculations on *Sm*AA10A

**Fig. 7:**
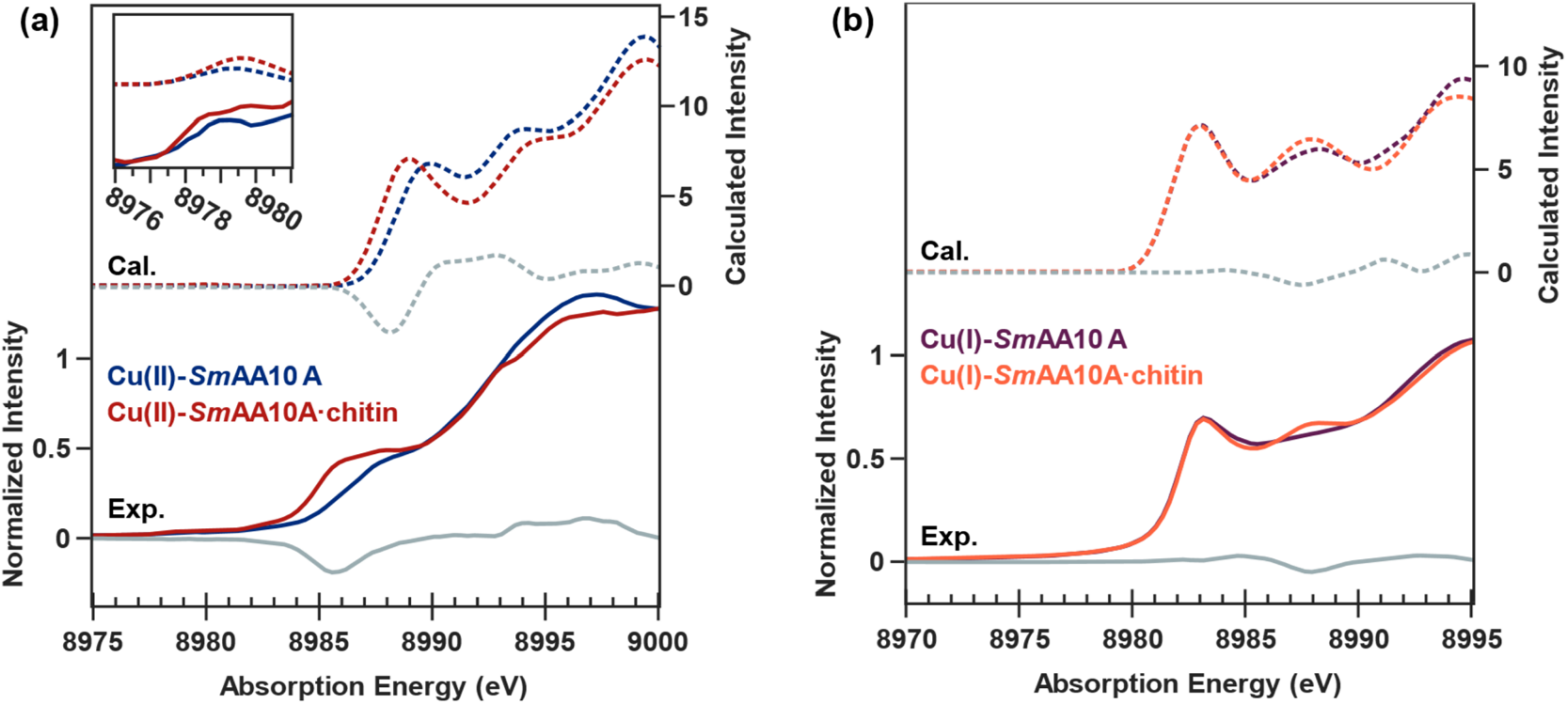
Experimental (solid lines) and calculated (dashed lines) Cu K-edge X-ray spectra for (a) Cu(ii)-*Sm*AA10A (blue) and Cu(ii)-*Sm*AA10A·chitin (red), and (b) Cu(i)-*Sm*AA10A (purple) and Cu(i)-*Sm*AA10A·chitin (orange). The experimental and calculated difference spectra (chitin-free minus chitin-bound) are shown in gray.

A comparison of the DFT-optimized models for Cu(ii)-*Sm*AA10A and Cu(ii)-*Sm*AA10A·chitin (Fig. S16a and b) demonstrates loss of a water ligand and overall contractions of the Cu–N distances (Table S11) in consequence of substrate binding (see remarks in SI, page S32). The spectral trends observed in the experimental Cu K-edge XAS data are well-captured in the calculated TDDFT spectra for the Cu(ii) systems (Fig. 7a), exhibiting a 0.3 eV shift to higher energy of the pre-edge feature when going from Cu(ii)-*Sm*AA10A to Cu(ii)-*Sm*AA10A·chitin. Additionally, the difference spectrum of the experimental data is consistent with the calculated difference data. Natural Transition Orbital (NTO) analysis of the pre-edge, which is conventionally attributed to electric dipole-forbidden quadrupole-allowed 1s → SOMO transitions in Cu(ii) sites, demonstrates the pre-edge feature in both structures to be primarily centered on the Cu site based on the Hirshfeld populations of the acceptor NTO (72% and 69% of Cu 3d for Cu(ii)-*Sm*AA10A and Cu(ii)-*Sm*AA10A·chitin, respectively) (Fig. S18). The mainly 3d_*x*^2^−*y*^2^_ composition of the SOMO displays an increased 4p admixture in Cu(ii)-*Sm*AA10A·chitin of 1.4% (*vs.* 0.5% in Cu(ii)-*Sm*AA10A), resulting in increased intensity of the pre-edge feature, which is consistent with the experimental data (Fig. 7a, inset). This increase suggests a lowering of the ligand-field symmetry associated with water ligand loss upon chitin binding. The contribution of d_z^2^_ to orbital composition exhibits a decrease from 2.2% to 0.1% upon chitin binding. Similarly consistent with the trends observed in the experimental data, the rising edge feature exhibits a 0.8 eV shift to lower energy. Several intense transitions contribute to the rising edge feature, and NTO analysis of these transitions reveals that the main acceptor NTO is primarily distributed over the Cu 4p and His28 imidazole π*. Because of the presence of several significantly contributing NTOs to the rising edge feature, the analysis of the edge spectrum features can be more practically analyzed by inspection of the transition polarization composition of each feature in the *x*, *y*, and *z* directions (Fig. S19), where the *x* axis is aligned along the Cu–N_term_ bond of the His28 primary amine, the *y* axis is set along the Cu–N^*δ*1^ bond of the His28 imidazole ring, and the *z* axis is approximately normal of the Cu–N–N–N plane. The rising edge feature near 8986 eV is predominantly *z*-polarized in both Cu(ii) systems, consistent with the primarily 4p_*z*_ character composition, while examination of the higher energy features reveals predominantly *x*- and *y*-polarization.

**Fig. 8:**
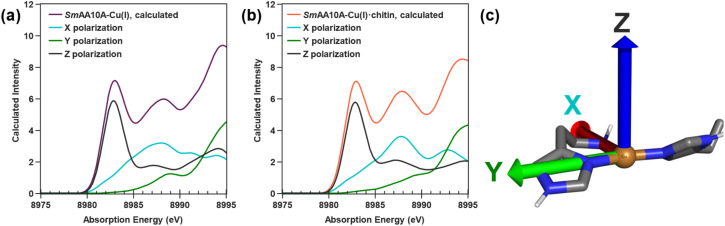
Calculated Cu K-edge XAS spectra with *xyz* polarization components for the models (a) Cu(i)-*Sm*AA10A and (b) Cu(i)-*Sm*AA10A·chitin. The cartesian orientation of the models is depicted in (c) on the right.

In contrast to the calculated Cu(ii) structures, the addition of chitin does not dramatically alter the Cu(i) site (Fig. S16c and d). The Cu–N_term_ distance of 2.19 Å in Cu(i)-*Sm*AA10A exhibits a slight elongation to 2.21 Å in Cu(i)-*Sm*AA10A·chitin. A more significant conformational change, however, can be observed involving the imidazole ligands upon chitin binding. Calculation of the least–square planes of the two rings and measurement of the dihedral angle between the two planes reveals a rotation from 59° in Cu(i)-*Sm*AA10A to 69° in Cu(i)-*Sm*AA10A·chitin. The calculated 1s-to-4p feature at 8983 eV in the TDDFT data is of similar intensity for both Cu(i)-*Sm*AA10A and Cu(i)-*Sm*AA10A·chitin (Fig. 7b), in agreement with the observed experimental spectra, and NTO analysis primarily attributes intensity to a transition with predominantly Cu 4p_*z*_ character (see remarks in SI, pages S35 and S36). The transition polarization analyses of both Cu(i)-*Sm*AA10A and Cu(i)-*Sm*AA10A·chitin (Fig. 8) support a primarily *z*-polarized assignment to the 8983 eV feature. Moving to higher energy, the calculated spectrum for Cu(i)-*Sm*AA10A·chitin exhibits a notable increase in intensity at 8988 eV relative to Cu(i)-*Sm*AA10A, consistent with the apparent emergence of such a feature in the experimental K-edge spectrum following chitin binding (Fig. 7b). While the spectral trends are in fact captured in the TDDFT-calculated data, the large number of excited states which compose the broad feature at 8988 eV hinders facile NTO analysis. Additionally, we note that these transitions, while modeled by TDDFT, correspond to high energy Rydberg and/or continuum states, which cannot reliably be assigned based on a molecular orbital picture.^89^ The transition polarization analysis data attributes the observed modulation of this feature at 8988 eV predominantly to an increase in *x*- and *z*-polarized intensities following chitin binding. Computations of the valence-to-core spectra were performed analogously using ground state DFT methods, and the results are provided in the SI, pages S37 and S38.

### Calculations using histidine brace toy models

In order to identify the subtle conformational changes behind the spectral differences between Cu(i)-*Sm*AA10A and Cu(i)-*Sm*AA10A·chitin (see Discussion), a suite of computational toy models was generated to characterize spectral trends as a function of various conformational perturbations. In the protein environment, multiple conformational parameters are likely interdependent, complicating their individual analysis. Toy complexes, however, provide a means to deconvolute these interdependencies. Therefore, four conformational parameters were chosen for investigation, namely the Cu–N_term_ distance and the angles *θ*_T_, *θ*_3_, and *θ*_D_ (see Fig. S24), based on their relevance in the description of histidine brace conformations found in LPMO diffraction data available in the Protein Data Bank.^33,90^ Four sets of histidine brace toy complexes were developed comprised only of Cu, His28, and His114 atoms, and structures were generated by iterating through each of the four conformational parameters. The effects of the histidine brace conformational changes to EXAFS data were calculated using the FEFF10 code to perform real-space Green's function (RSGF) theory calculations (Fig. S25). Only changes to the Cu–N_term_ distance incurred any significant modulations of the calculated EXAFS. Dramatic changes in the *θ*_T_, *θ*_3_, or *θ*_D_ angles, on the other hand, resulted in only negligible modulations to the calculated EXAFS spectra. The analogous investigation using TDDFT to determine the conformational effects to the calculated Cu K-edge spectra (Fig. S26) demonstrated that *θ*_T_ results in intensity changes exclusively at the lower energy 8983 eV feature. In contrast, iteration along *θ*_3_ or *θ*_D_ modulates intensity primarily at the 8988 eV feature, similar to modulations observed in the experimental data, and changes in Cu–N_term_ and *θ*_D_ resulted in subtle modulation to the sharpness of the 8983 eV feature. Finally, DFT calculation of the VtC-XES spectra demonstrate the emission spectra to be considerably sensitive to *θ*_T_ and *θ*_3_ in the Kβ_2,5_ region while relatively insensitive to changes in Cu–N_term_ distance or *θ*_D_ (Fig. S27).

The collective of experimental X-ray spectra (Cu K-edge spectra, EXAFS, and VtC-XES) can be considered in the context of the data calculated from the histidine toy complexes. This allows us to determine the most likely and significant conformational change induced at the Cu(i) site in Cu(i)-*Sm*AA10A following chitin binding by systematic process of elimination. As reported above, minimal perturbation is observed in the experimental EXAFS data between Cu(i)-*Sm*AA10A and Cu(i)-*Sm*AA10A·chitin (Fig. 5b). The RSGF calculations, therefore, eliminate the possibility that the Cu–N_term_ distance changes significantly upon chitin binding (Fig. S25). Similarly, the observed modulations in the experimental Cu K-edge XAS data at 8988 eV (Fig. 5a) are inconsistent with the changes calculated from the TDDFT when adjusting Cu–N_term_ distance and *θ*_T_ in the toy complexes (Fig. S26). Notably, the spectral modulations reflected in an increasing *θ*_D_ angle best represent the modulations in the experimental data. However, *θ*_3_ cannot yet be eliminated. Finally, the minimal spectral modulations observed in the experimental VtC-XES (Fig. 6) eliminate the likelihood of changes in *θ*_3_ and reconfirm the elimination of *θ*_T_, based on the calculated VtC-XES for the toy complexes (Fig. S27). Furthermore, the mild spectral perturbations again suggest an increasing *θ*_D_ angle, though we acknowledge that these changes fall within the standard error of the valence-to-core experiment. Taken together, the computed Cu K-edge spectra, EXAFS, and VtC-XES strongly suggest a conformational change of a more perpendicular dihedral angle *θ*_D_ between the imidazole rings following substrate binding (Fig. 11b). Importantly, this is consistent with the geometric parameters observed in the optimized Cu(i) structures (Table S11), wherein *θ*_D_ displays significant changes, while the other conformational parameters remain largely unchanged.

### Binding and activation of H_2_O_2_

To estimate the impact and functional implications of the conformational change in the histidine brace following chitin binding, the H_2_O_2_ binding energies and the reaction coordinate pathways for H_2_O_2_ activation by the Cu(i)-*Sm*AA10A and Cu(i)-*Sm*AA10A·chitin models were calculated. Firstly, our study reconfirms the previously reported role of Glu60 in the binding and activation of H_2_O_2_,^13^ identifying two H-bond interactions between H_2_O_2_ binds and Glu60 in both models (Fig. S28). Absent, however, is the H-bond with the chitin *N*-acetyl group (in Cu(i)-*Sm*AA10A·chitin), likely due to increased model rigidity for cluster models (compared to QM/MM). Nevertheless, even without this additional H-bond to the *N*-acetyl group, the binding energies of Cu(i)-*Sm*AA10A and Cu(i)-*Sm*AA10A·chitin diverge slightly: Δ*E*_bind_ = −19.7 kcal mol^−1^ for the former and Δ*E*_bind_ = −21.5 kcal mol^−1^ for the latter (Table S12). The resulting ΔΔ*E*_bind_ = −1.8 kcal mol^−1^ indicates favorable H_2_O_2_ binding in the LPMO-chitin complex. The binding enthalpy and free energy follow the same trend, hinting towards favorable H_2_O_2_ binding in the chitin-bound model. We note, however, that the ∼2 kcal mol^−1^ difference of is within the error limits of DFT. Additionally, the Hessian calculations of Cu(i)-*Sm*AA10A·chitin are more affected by the limitations imposed by the increased number of constraints relative to Cu(i)-*Sm*AA10A. Thus, future studies employing more sophisticated QM/MM methods with explicit solvation models will be necessary to substantiate these results.

Following this, we explored the differences in the H_2_O_2_ activation pathway in Cu(i)-*Sm*AA10A and Cu(i)-*Sm*AA10A·chitin models. In Cu(i)-*Sm*AA10A·H_2_O_2_, the optimized structure of the reactant complex (^1^RC) (Fig. 10 and S29) revealed that both hydrogen atoms of H_2_O_2_ are hydrogen bonded to the carboxylic group of Glu60 in a strained conformation with a dihedral angle of 33°. The O–O bond length is 1.46 Å and the Cu–O1 (proximal oxygen atom to Cu center) distance is 3.79 Å. By comparison, ^1^RC of the chitin-bound model, Cu(i)-*Sm*AA10A·chitin·H_2_O_2_, displays a similar H_2_O_2_ binding mode and dihedral angle. Additionally, the proximal oxygen is 0.03 Å (3.76 Å) closer to Cu, while the O–O bond length remains unchanged (Table S13).

The transition state (^1^TS1) for Cu(i)-*Sm*AA10A·H_2_O_2_ on the broken-spin singlet surface occurs at an O–O bond length of 1.72 Å, with an electronic energy barrier of 16.4 kcal mol^−1^ relative to the initial minimum. Interestingly, at this point, the H_2_O_2_ dihedral angle increases to 114°, and only one hydrogen bond remains between H_2_O_2_ and Glu60 (with the proton of the distal oxygen, Fig. S29). After homolytic cleavage, the resulting intermediate complex (^1^IC1) consists of a Cu(ii)–OH^−^ species and an OH˙ radical, with the latter hydrogen-bonded to Glu60. This interaction is indicated by a higher spin population on the Cu center (3d_*x*^2^−*y*^2^_) and on the distal oxygen atom (Fig. S30). Notably, the hydroxyl radical is stabilized by its hydrogen bond with Glu60.

**Fig. 9:**
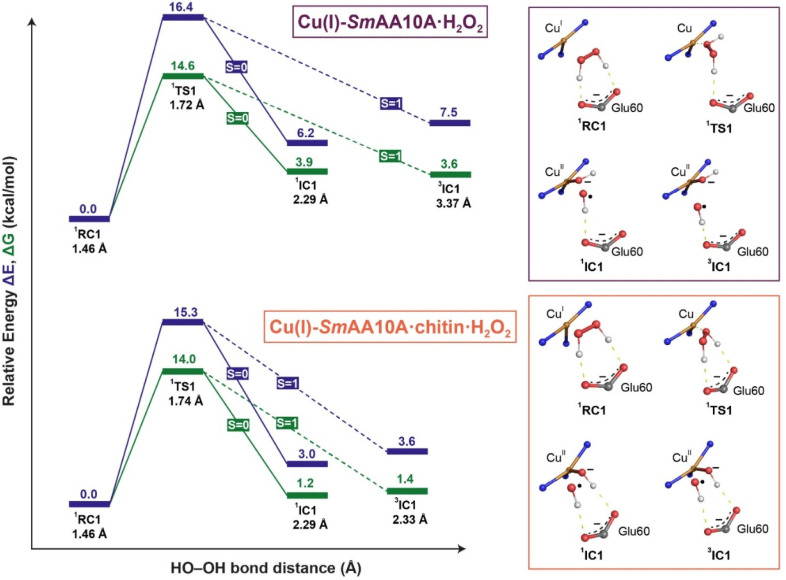
Relative electronic energetics in kcal mol^−1^ of minima and transition states along the H_2_O_2_ activation pathway in the Cu(i) state in Cu(i)-*Sm*AA10A·H_2_O_2_ (top) and Cu(i)-*Sm*AA10A·chitin·H_2_O_2_ (bottom). The electronic energies (Δ*E*) are depicted in indigo, and the free energies (Δ*G*) are depicted in green. Solid lines indicate singlet open shell singlet (*S* = 0) and dashed lines indicate triplet manifold (*S* = 1), respectively. RC1 = reactant complex, TS1 = transition state, IC1 = intermediate complex. Spin multiplicity is indicated by the left superscript. The O–O bond distance (Å) of H_2_O_2_ is indicated in black for the ^1^RC1, ^1^TS1, ^1,3^IC1. The respective ^1^RC1, ^1^TS1, and ^1,3^IC1 are shown as reaction schematics on the left for Cu(i)-*Sm*AA10A·H_2_O_2_ (top) and Cu(i)-*Sm*AA10A·chitin·H_2_O_2_ (bottom). Hydrogen bonds are shown as dashed yellow lines.

In chitin-bound Cu(i)-*Sm*AA10A·chitin·H_2_O_2_, the ^1^TS1 state (Fig. S31) is located at an O–O bond length of 1.73 Å, with an electronic energy 1.1 kcal mol^−1^ lower than the chitin-free model. In this transition state, H_2_O_2_ maintains two hydrogen bonds to Glu60, involving both proximal and distal oxygen protons (Fig. 9). The distance between Cu and the closest Glu60 head group oxygen is shortened by more than 1 Å compared to in the Cu(i)-*Sm*AA10A·H_2_O_2_ in the ^1^TS1 state (Fig. S33b and Table S13). We also note a 0.4 Å elongation in Cu–Phe187 distance in Cu(i)-*Sm*AA10A·chitin·H_2_O_2_ compared to chitin-free model, likely attributable to the strain imposed on the Phe187 residue by the shortened Glu60–Cu distance. For the intermediate species (^1^IC1 and ^3^IC1), the geometric structures remain similar to those observed in Cu(i)-*Sm*AA10A·H_2_O_2_, with the ^3^IC1 having the O–O bond 1.03 Å shorter than in the Cu(i)-*Sm*AA10A·H_2_O_2_ model. In both spin states, the OH^−^ species forms a H-bond interaction with Glu60, which was absent in the Cu(i)-*Sm*AA10A. This makes the product state in the chitin-bound model more stabilized (ΔΔ*E* = 3.2 kcal mol^−1^) than in Cu(i)-*Sm*AA10A·H_2_O_2_. Interestingly, the Cu–Phe187 distance shortens back to 3.66 Å. The more stabilized products (IC1) in the chitin-bound model and shorter distance between Cu and the proximal oxygen in ^1^RC1 translate to a lower activation energy for O–O bond cleavage.

## Discussion

### Water ligand dissociation at the Cu(ii) site

The EPR, X-ray absorption K-edge, EXAFS, and computational data for Cu(ii)-*Sm*AA10A and Cu(ii)-*Sm*AA10A·chitin are all consistent with the loss of a water ligand at the Cu(ii) site upon chitin binding. A decrease in coordination number upon chitin binding is clearly demonstrated in the EXAFS data, and DFT calculations reproduce the trends in the Cu K-edge spectra following removal of one of the two water ligands. Previous Cu K-edge XAS studies on small-molecule Cu(ii) complexes, as well as the Cu(ii)-containing blue copper proteins, have demonstrated correlations between coordination number and axial geometry with changes in edge shape.^81,91,92^ In *Sm*AA10A, an apparent shift to lower energy of the rising edge shoulder feature from 8988.6 eV to 8986.4 eV can be observed upon chitin binding (Fig. 3). The distorted square planar geometry following the loss of one water ligand stabilizes the 4p_*z*_ orbital thus shifting the envelope of the rising edge transitions to lower energy, as also predicted by the TDDFT modeling. While the calculated transitions in the pre-edge regions are generally localized and can often be assigned to only a few (or even one) discrete NTO, transition compositions upon approaching the rising edge become significantly more diffuse. Therefore, transition polarization composition analysis is advantageous in the interpretation of this regime. The rising edge main feature is predominantly *z*-polarized for both Cu(ii) systems, consistent with the 4p_*z*_ character. The adjacent higher-energy features in the TDDFT data are *x*- and *y*-polarized, respectively. The *z-x-y* energy order reflects the directional ligand field strengths such that the *z*-direction features fewer and longer bonds (with solvent), while the *y*-direction features the strongest ligand field due to shorter bonds with the imidazole rings. Following the loss of a solvent-facing water ligand and incorporation of the remaining water ligand into the equatorial *x*,*y* plane, the *z*-polarized feature shifts lower in energy while, conversely, the *x*- and *y*-polarized features shift to higher energies.

**Fig. 10:**
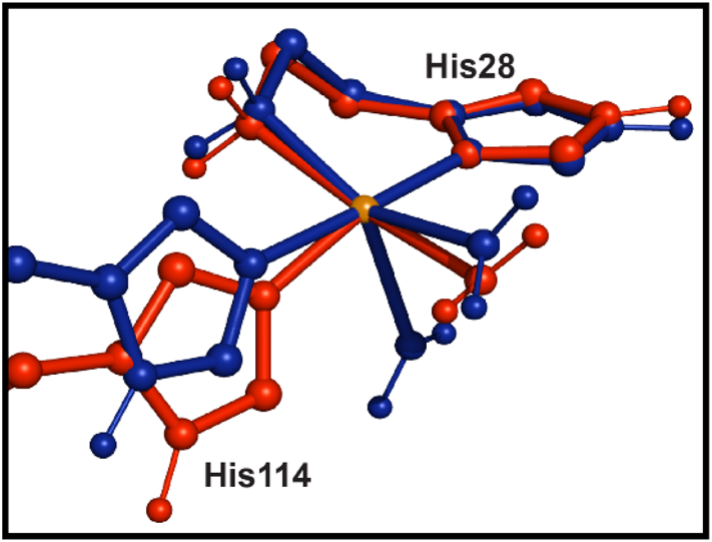
Overlay of Cu(ii)-*Sm*AA10A (blue) and Cu(ii)-*Sm*AA10A·chitin (red). The Cu site is shown in orange. Following the loss of a water ligand, the previously rhombic geometry observed in Cu(ii)-*Sm*AA10A (blue) converts to a more axial geometry in Cu(ii)-*Sm*AA10A·chitin (red) as the remaining ligands adopt a planar geometric arrangement.

Consistent with previous reporting,^31^ the initial rhombic geometry observed in the EPR for Cu(ii)-*Sm*AA10A transitions towards that of an axial Cu(ii) center in Cu(ii)-*Sm*AA10A·chitin (Fig. 2), suggesting decreased mixing of d_*z*^2^_ with d_*x*^2^−*y*^2^_. This decreased mixing is additionally supported by the K-edge data, which shows broadening of the pre-edge feature upon chitin-binding, as well as a 0.6 eV shift to higher energy of the pre-edge maximum. The NTO analysis assigns this feature to a transition orbital which is primarily composed of 3d_*x*^2^−*y*^2^_ character and exhibits a sharply decreased 3d_*z*^2^_ contribution (2.2% to 0.1%) upon substrate binding. The shift of the primarily d_*x*^2^−*y*^2^_ SOMO to higher energy, therefore, results in an increased splitting between the d_*z*^2^_ and d_*x*^2^−*y*^2^_ energies, rationalizing the decreased mixing evidenced in the EPR. We note that similar EPR-detected geometric transitions have been previously reported in the pH-dependent behaviors of other AA10 LPMOs, whereby increasing the pH environment of the protein converts a rhombic spectrum at pH ∼6 into a clearly axial signal at pH ∼8–10.^93,94^ EPR data on collected on AA9 LPMOs treated with soluble oligosaccharides in chloride-containing buffers has been previously reported, showing only subtle changes to the g-tensor following substrate binding,^6,95^ consistent with electronic structure calculations on *Ls*AA9A.^34^ Our EPR data on *Sm*AA10A, however, presents a dramatic example of the conversion from rhombic to axial upon chitin binding (Fig. 10), and the corresponding Cu K-edge, EXAFS, and TDDFT data indicate significant geometric and electronic structure changes consistent with the loss of a water ligand and rearrangement around the Cu(ii) site.

### Reduction mechanism insights from Cu site photosensitivity modulations

The X-ray damage susceptibility of Cu(ii)-containing metalloproteins, including pMMO and LPMOs, is a well-documented phenomenon in bioinorganic X-ray spectroscopy and X-ray crystallography.^10,33,42,78,96^ In XAS, photoreduction induces Cu(ii) → Cu(i) conversion, which manifests as an emergent feature at ∼8983 eV just before the Cu(ii) rising edge. Our investigation found that increasing the chitin-bound component of the sample composition yields increasingly photoreduced spectra, suggesting that Cu(ii)-*Sm*AA10A·chitin displays increased X-ray photosensitivity relative to the unbound species. This suggests that Cu(ii)-*Sm*AA10A·chitin has an enhanced reduction rate and a positively shifted reduction potential compared to Cu(ii)-*Sm*AA10A. The X-ray photoreduction rate of a metalloenzyme does not necessarily correlate with its reduction potential, though such trends have emerged when comparisons are constrained to highly similar active sites.^97–99^

Support for the above hypotheses can be derived from comparison of the damage behavior and reported reduction potentials between Cu(ii)-*Sm*AA10A and Cu(ii)-*Nc*AA9C. We previously reported a positively shifted reduction potential of 275 mV (*vs.* NHE) for *Sm*AA10A compared to 211 mV for *Nc*AA9C.^17^*Sm*AA10A also exhibits an enhanced rate of reduction by ascorbate of 4.2 × 10^5^ M^−1^ s^−1^*vs.* 1.4 × 10^5^ M^−1^ s^−1^ in *Nc*AA9C. Conversely, XAS data collection for Cu(ii)-*Sm*AA10A in this study utilized 0.2% of the total available flux at Diamond I20-scanning, while Cu(ii)-*Nc*AA9C could withstand 1.2% of the total flux with the same scan parameters, beamline configuration, and storage ring current,^17^ demonstrating an increased susceptibility in Cu(ii)-*Sm*AA10A to X-ray photoreduction. Therefore, the increased susceptibility of substrate-bound Cu(ii)-*Sm*AA10A·chitin to X-ray photoreduction may in fact correspond to a slightly more positively shifted (*i.e.*, anodically shifted) reduction potential, relative to both Cu(ii)-*Sm*AA10A and Cu(ii)-*Nc*AA9C. Additional precedence for an anodic shift in reduction potential following substrate binding has been reported for *Nc*AA9C in the presence of soluble xyloglucan (+64 mV shift).^22^ We note that this hypothesis does follow some chemical intuition when comparing the 5-coordinate Cu(ii)-*Sm*AA10A Cu(ii) site against the 4-coordinate site of Cu(ii)-*Sm*AA10A·chitin. Considering that both species reduce to 3-coordinate Cu(i) sites, the geometric relaxation required for reducing the 4-coordinate site is expected to be energetically less than that of the 5-coordinate site. Furthermore, the dissociation of a water ligand and confinement of the solvent-facing Cu ion into the protein-substrate enclosure in Cu(ii)-*Sm*AA10A·chitin decreases the local dielectric constant, increasing the reduction potential of the Cu site.^92^

Such a hypothesis can then be extrapolated into an interesting, albeit speculative, line of reasoning. As of yet, the identity of the physiologically relevant reductant(s) for AA10 LPMOs is unknown, leading to the use of common laboratory reductants with rather large overpotentials for LPMO reduction, the utilization of which has been shown to introduce convoluting factors in LPMO reactivity studies.^37,100,101^ Furthermore, once the reduced Cu(i)-LPMO is generated, it has the possibility to proceed down one of two pathways upon reaction with an H_2_O_2_ co-substrate molecule (Fig. 1d). If the LPMO is substrate-bound, the highly oxidizing potential of the copper-oxyl intermediate formed can be directed towards productive substrate hydroxylation. However, when the LPMO is not substrate bound, it has been shown to enter into off-pathway turnover mechanisms, in which the highly reactive species may instead result in LPMO inactivation if it is not diverted safely away from the active site *via* a tyrosyl/tryptophanyl hole-hopping pathway.^20–22,24,25,30,79^ The shift to a more positive reduction potential in Cu(ii)-*Sm*AA10A·chitin relative to Cu(ii)-*Sm*AA10A introduces a “reduction potential window.” In the biological setting, it would therefore be highly advantageous if the natural LPMO reductant choice were to minimize the overpotential for reduction of the chitin-bound Cu(ii) species. The consequence that follows would be a catalytic system by which primarily substrate-bound Cu(i) sites are formed, primed for productive turnover.

### Conformational changes at the Cu(i) site influence reactivity

**Fig. 11:**
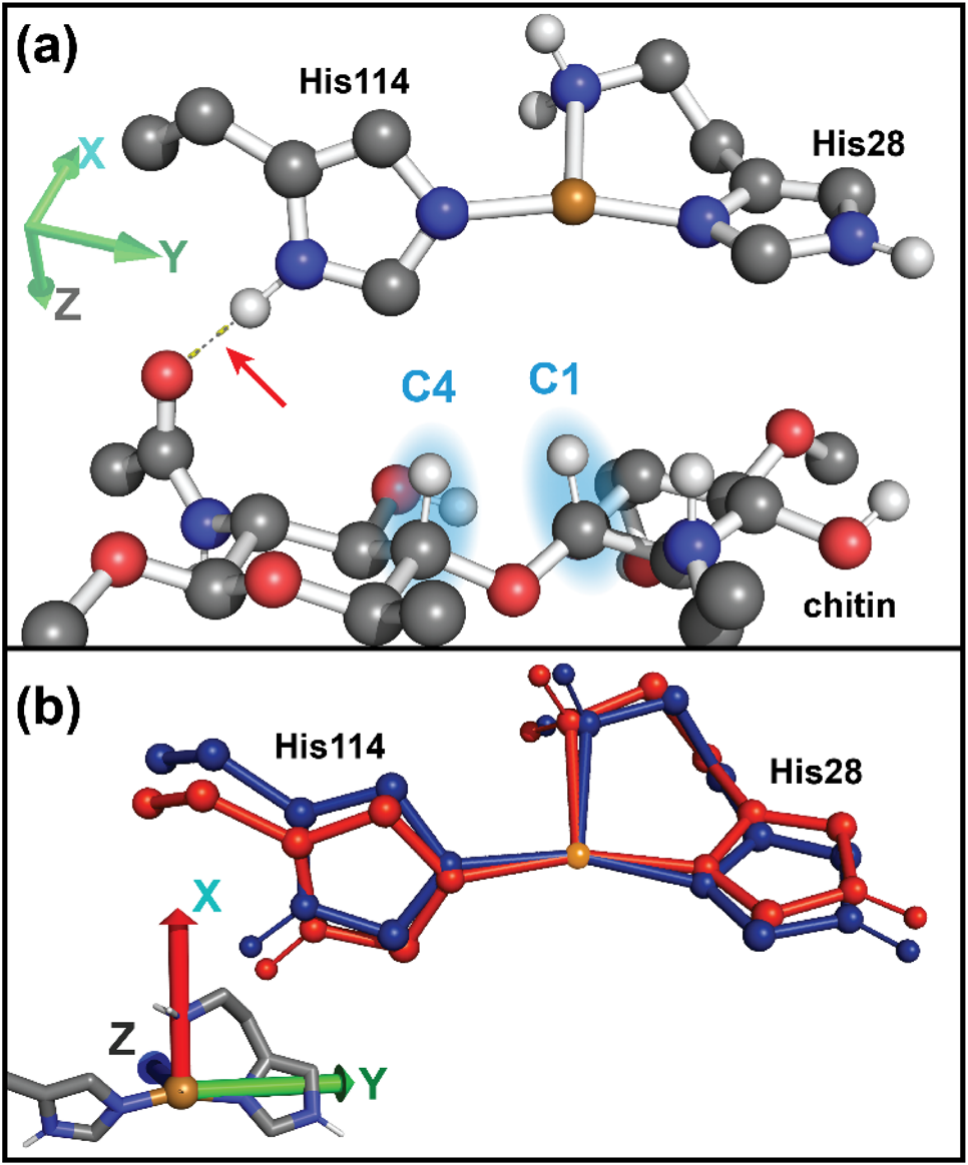
(a) Optimized structure for Cu(i) *Sm*AA10A·chitin indicating the hydrogen-bonding interaction (red arrow) between N^*δ*1^_His114_ and an acetamidyl O from chitin. (b) Overlay of Cu(i)-*Sm*AA10A (blue) and Cu(i)-*Sm*AA10A·chitin (red) depicting the change induced in His114 upon chitin binding. The Cu site is depicted in orange.

Although the Cu(ii) samples lend themselves to thorough investigation by both EPR and X-ray techniques, the Cu(i) pair is of particular interest for its role as the reductively primed species in the LPMO catalytic cycle. It is the Cu(i) state of the protein that proceeds down either a productive catalytic pathway or off-pathway turnover (Fig. 1d). The closed-shell d^10^ Cu(i)-containing species are invisible to EPR spectroscopy, for which an open-shell system is a strict requirement for detection. X-ray spectroscopic methods, however, are uniquely suited to investigate both the solution-state and chitinous Cu(i) proteins. While the lack of an EPR handle hindered our ability to quantifiably ascertain the extent of chitin-binding, we note that previous precedent with *Nc*AA9C has suggested an increased substrate-binding affinity in the reduced-state LPMO.^35^ The K-edge, EXAFS, and DFT data suggest both Cu(i)-*Sm*AA10A and Cu(i)-*Sm*AA10A·chitin to be three-coordinate Cu(i) centers, with a slightly elongated Cu–N bond length to the terminal amine relative to the imidazole Cu–N bonds. Upon conversion from Cu(i)-*Sm*AA10A to Cu(i)-*Sm*AA10A·chitin, clear electronic structure modulations are indicated by changes in the K-edge data: a decreased intensity of the shoulder feature at 8985 eV and, most notably, the emergence of a higher-energy feature at 8988.1 eV. Although the higher-energy feature cannot be reliably assigned based on a molecular orbital picture, transition polarization analysis shows this spectral region to be dominated by *x* polarized transitions. The increased intensity of this feature upon chitin binding from the *x*,*z* direction (see Fig. 11 for orientation) can be associated with two factors: (1) the orientation of the His-brace π* system being more aligned with the Cu 4p_*x*_ due to an increase in *θ*_D_ (increased perpendicularity), and (2) the increase in the Cu–N_term_ distance which localizes the 4p_*x*_ more onto the Cu, increasing its 4p character.

Due to the combination of weak signal intensity of VtC features and the typically dilute samples when working with metalloproteins, only a few examples of VtC-XES on a Cu protein have been reported, including one report on a frozen solution sample of *Hj*LPMO9A.^86–88^ Our study provides the first application of VtC-XES towards characterizing an LPMO bound to a polysaccharide substrate. It is worth noting that, as a probe for low-lying unoccupied orbitals, XAS for d^10^ Cu(i) is sensitive to changes in the electronic structure of the more diffuse antibonding orbitals. This stands in contrast with VtC-XES, which serves as a more local probe of relatively contracted bonding orbitals. Nevertheless, conversion of Cu(i)-*Sm*AA10A to Cu(i)-*Sm*AA10A·chitin manifests as only subtle perturbations in the observed VtC features. Molecular orbitals analysis of the VtC features assigns these perturbations to the N 2p orbital on the His114 imidazole for the 8974.5 eV feature at lower energy and to the bonding 3d_*x*^2^−*y*^2^_ orbital for the higher energy 8978 eV feature. This is further confirmed by the strong *y* polarization contribution of the lower energy feature and the primarily *x* and *y* composition of the transition polarization of the higher energy feature. The notable absence of the 3d_*z*^2^_ in the VtC molecular orbitals analysis and lack of *z* transition polarization contribution to the VtC features suggests the 3d_*z*^2^_ is a non-bonding orbital. In addition to the assigned VtC features in the emission spectrum, a higher-energy, lower-intensity feature was observed at ∼8983 eV in both spectra, which is not captured by the DFT calculations. Recent studies on Cu VtC have attributed this feature to a KMβ emission originating from a 1s + 3d double ionization event,^87^ and similar assignments have been determined in the emission spectra of Ni and Zn.^102,103^ However, the electronic structure origin of this feature remains unclear, and furthering theoretical methods towards reliably reproducing these features would provide significant insight into transition metal electronic structure and bonding.^103^

Despite the clear electronic structure modulations indicated by the K-edge spectrum, the *k*^3^-weighted EXAFS data indicate only minor geometric perturbations at the Cu(i) site upon chitin binding to Cu(i)-*Sm*AA10A. Thus, we concluded that no major bond formation or breaking occurs. Rather, the analysis of the EXAFS data correlate chitin binding with likely conformational changes of the imidazole side chains of His28 and His114 ligating the copper. In order to decipher these structural changes, a series of histidine brace toy models were generated to investigate four conformational parameters: Cu–N_term_, *θ*_T_, *θ*_3_, and *θ*_D_ (Fig. S24). Taken together, evaluation of the near-edge XAS (TDDFT), XES (DFT), and EXAFS (FEFF) suggests *θ*_D_ (the dihedral angle between the imidazole ring planes) to describe the most prominent conformational change. Indeed, examination of the geometry-optimized protein models does in fact exhibit a notable contortion of the *θ*_D_ from 59° to 69° upon chitin binding, induced by a hydrogen bonding interaction of the N^*δ*1^ from His114 with an acetamidyl O from chitin (Fig. 11). In contrast, comparison of the *θ*_D_ values for the C4-regioselective *Ls*AA9A, reported by Tandrup *el al.* (2022),^33^ shows an opposite trend from 59° to 55° for Cu(i)-*Ls*AA9A and Cu(i)-*Ls*AA9A·Cell_4_ (Cell_4_ = cellotetraose), respectively (PDB: 7pxv, 7pyi). This may be partially attributed to the lack of the acetamide group in the cellulosic substrate. We note that the electron withdrawing hydrogen bonding interaction in *Sm*AA10A would presumably increase the covalency of the imidazole ligand with the Cu ion. However, rotation of the dihedral angle to a more perpendicular conformation would decrease overlap between the Cu p_*x*_ and the imidazole ring π*, leading to competing effects acting upon the Cu–N_His114_ bond. In this regard, it is most interesting that *Sm*AA10A demonstrates regioselective activation of the C1 site over the C4 site,^1,104^ and the hydrogen bonding interaction illustrated in Fig. 11a could perhaps explain this selectivity, since it helps to position the Cu site to interact with the C1 site. However, further experimental and computational explorations into the role and dynamics of this interaction in *Sm*AA10A, as well as other LPMOs featuring alternative regioselectivities, are needed to understand these behaviors.

A computational investigation by Lim *et al.* suggested that conformational effects on the histidine brace may play a role in raising the energy of the Cu(i) site and thereby lowering the barrier for H_2_O_2_ binding and activation compared to the broken His-brace models by about 3 kcal mol^−1^ (Δ*G*).^88^ Therefore, we examined the binding of H_2_O_2_ to Cu(i)-*Sm*AA10A in both the free and chitin-bound states to understand how structural changes induced by chitin binding influence reactivity. The results reveal a more favorable binding energy for H_2_O_2_ in the chitin-bound LPMO compared to the free enzyme in terms of Δ*E*, Δ*H*, or Δ*G*. While the absolute values may be subject to the choice of the solvation method, the energy difference between free and bound models should approximately cancel out the solvation effect. The binding of H_2_O_2_ to the chitin-bound LPMO model is favored by 2 kcal mol^−1^ according to the DFT method employed here. We note, however, that the increased structural constraints in the chitin-bound model impose additional rigidity on the histidine brace and surrounding residues, causing its Hessian calculation to be more severely affected by the increased number of constraints than the chitin-free model. Nevertheless, the results do indicate that chitin binding stabilizes the interaction of H_2_O_2_ with the Cu(i) site, potentially optimizing its geometry for subsequent catalytic steps. We note that a stabilizing effect has been proposed in an AA9 LPMO when bound to cellulose oligosaccharide substrate in electronic structure calculations, whereby the presence of substrate lessens the energy gap between the Cu d_*x*^2^−*y*^2^_ and H_2_O_2_ σ* orbitals.^34^ These findings therefore suggest that Cu site reactivity is modulated by substrate binding, providing a mechanism that would serve to prevent potentially harmful overactivity when the substrate is absent.

To understand how chitin binding influences the H_2_O_2_ activation pathway, we analyzed structural and electronic differences between the Cu(i)-*Sm*AA10A·H_2_O_2_ and Cu(i)-*Sm*AA10A·chitin·H_2_O_2_ states. The results suggest that while H_2_O_2_ binds similarly in both models, chitin binding introduces subtle but important changes that lower the energy barrier for O–O bond cleavage and enhance product stabilization. In both systems, H_2_O_2_ initially interacts with Glu60 through two hydrogen bonds, with a strained dihedral angle (30 and 33°) and longer O–O bond distance (1.46 Å) relative to a free H_2_O_2_ (dihedral: 116°, bond: 1.45 Å) optimized at the same theory level. Upon progressing to the first transition state (^1^TS1), the electronic energy barrier is reduced by 1.1 kcal mol^−1^ in the chitin-bound model (Fig. 9). While this energy difference does fall within the range of uncertainty for DFT, the observed decrease in the energy barrier is consistent with stabilization of the transition state by the presence of chitin, facilitating O–O bond cleavage. Notably, the H_2_O_2_ dihedral angle remains more constrained (37°) in Cu(i)-*Sm*AA10A·chitin, due to the double hydrogen bonding with Glu60, probably associated with the closer distance between Glu60 head group and Cu. These factors contribute to the enhanced stabilization of the transition state relative to the chitin-free model. The final product state is also more stabilized in the presence of chitin (ΔΔ*E* = −3.2 kcal mol^−1^), supporting the hypothesis that modulations in the histidine brace after chitin binding enhances the reactivity of LPMO by lowering the activation barrier and stabilizing reaction products. Interestingly, in the Cu(i)-*Sm*AA10A·chitin reported by Bissaro *et al.*,^13^ the Cu–O1 distance in ^1^RC is more than 1 Å lower (2.98 Å) than our finding, and the H_2_O_2_ dihedral angle is 53°, significantly larger than what we report. Additionally, the ^1^TS1 reported in this study does not maintain two hydrogen bonds with Glu60 and the H_2_O_2_ dihedral angle is further relaxed to 94°. Due to this, the energy of the products in the ^1,3^IC1 states is similar to those reported here for the chitin-free model. The differences may be attributed to the smaller basis set (def2-SVP) used in the optimization protocol in the previous study.^13^ Nevertheless, we acknowledge that the findings disclosed in this study would certainly benefit from future validation studies employing more sophisticated QM/MM methods with explicit solvation models.

Overall, we have demonstrated that a conformational effect does occur at the Cu(i) site following chitin binding, and these findings are grounded in experimental X-ray spectroscopic evidence. This conformational change manifests as increased transition polarization into the *x*,*z* plane observed along the rising edge upon chitin binding. The computational analysis suggests that chitin binding modulates the conformation of the active site, changes the electronic environment of Cu, enhances O–O bond dissociation rate, and stabilizes the reaction intermediates. Notably, this is in qualitative agreement with the experimental kinetic rates, which show increased turnover of H_2_O_2_ by *Sm*AA10A when chitin substrate is present.^13,26^ This could have important implications for LPMO catalysis, as substrate binding not only orients the enzyme for oxidative cleavage but also tunes the electronic properties to optimize reactivity. Based on the observed electronic structure changes deduced from the K-edge data for Cu(i)-*Sm*AA10A and Cu(i)-SmAA10A·chitin, it is clear that a true understanding of the LPMO electronic structure and mechanism during on-pathway, catalytic turnover requires experimentally characterizing the system in the presence of polysaccharide substrate.

## Concluding remarks

In this study, we utilized a suite of advanced spectroscopic and computational techniques, including EPR, Cu K-edge XAS, EXAFS, VtC-XES, (TD)DFT, and real space Green's function theory (using FEFF10 code) calculations, to elucidate the effects of chitin binding on both the Cu(ii) and Cu(i) sites of the *Sm*AA10A LPMO. These methods provided detailed insights into the structural and electronic alterations induced by substrate binding and mark the first X-ray spectroscopic characterization of a substrate-bound LPMO. Our findings underscore the utility of X-ray techniques in examining heterogeneous, closed-shell systems, such as LPMOs, which operate on insoluble polysaccharide substrates, thus limiting the applicability of conventional bio-inorganic spectroscopic techniques. The data are consistent with dissociation of a water ligand from the Cu(ii) center upon chitin binding, possibly resulting in an anodic shift in reduction potential. In the reduced Cu(i) state, we observed a subtle, but important, conformational change: an increasingly perpendicular imidazole dihedral angle correlated with a hydrogen bonding interaction, which may help explain the observed regioselectivity for activation at the chitin C1 site. Our reactivity calculations highlight the implications of the conformational change on H_2_O_2_ activation, whereby the more favorable H_2_O_2_ binding energy, the transition state stabilization, and the more stabilized products of the H_2_O_2_ splitting imply enhanced reactivity when chitin is bound. Overall, our combined spectroscopic and computational results provide an atomistic-level insight into the impact of substrate binding on the LPMO copper center and its reactivity, deepening our understanding of LPMO function and substrate specificity.

## Author contributions

SD and VGHE conceived and supervised the project. CJ performed the sample preparation, chitin-binding assays, EPR & X-ray spectroscopy data collection & processing, and RSGF calculations under the supervision of SD. AT performed DFT and TDDFT calculations, including calculation of optimized geometries, K edges, VtC, and H_2_O_2_ activation with data provided from ÅKR and under the supervision of SAVJ and SD. OG expressed and purified apo-*Sm*AA10A under the supervision of MS and VGHE. KS and LK contributed to the X-ray data collection. CJ and AT prepared the initial draft of the manuscript. All authors contributed to discussing the results and provided input on the manuscript.

## Conflicts of interest

There are no conflicts to declare.

## Supplementary Material

SC-016-D5SC07620J-s001

## Data Availability

The data utilized in this study will be available for download on Edmond, the open data repository of the Max Planck Society, at https://doi.org/10.17617/3.YCRTYS. Supplementary information (SI): experimental procedures, figures, tables, and remarks referred to throughout this text. Atomic coordinates and sample ORCA input files. See DOI: https://doi.org/10.1039/d5sc07620j.
