# Supplementary material for: Structural and electronic modulations of lytic polysaccharide monooxygenase (LPMO) upon chitin binding: insights from X-ray spectroscopy
Source: Chem Sci. 2025 Nov 19;16(48):22952–69. doi: 10.1039/d5sc07620j (PMC12658904; doi:10.1039/d5sc07620j)
Supplement: SC-016-D5SC07620J-s001 [file SC-016-D5SC07620J-s001.pdf]

This journal is © The Royal Society of Chemistry 2025

Supporting Information for:

Structural and Electronic Modulations of Lytic Polysaccharide Monooxygenase (LPMO) upon Chitin Binding: Insights from X-ray Spectroscopy

Chris Joseph,[1] Ashish Tamhankar,[1] Ole Golten,[2] Kushal Sengupta,[1] Sergio A. V. Jannuzzi,[1] Morten Sørlie,[2] Liqun Kang,[1] Åsmund K. Røhr,[2] Vincent G. H. Eijsink,[2*] Serena DeBeer[1*]

[1] Max Planck Institute for Chemical Energy Conversion, Stiftstraße 34–-36, 45470, Mülheim an der Ruhr, Germany

[2] Faculty of Chemistry, Biotechnology and Food Science, Norwegian University of Life Sciences (NMBU), Ås, Norway.

*Correspondence to: serena.debeer@cec.mpg.de, vincent.eijsink@nmbu.no

**Contents**	**Page**
**Additional Experimental Procedures**	S3–S7
**Table S1.** Incubation conditions tested for optimal chitin binding to **Cu(II)-*Sm*AA10A**	S8
**Figure S1.** EPR spectra of sample cells collected prior to X-ray experiments	S9
**Table S2.** X-band EPR experimental scan parameters	S10
**Table S3.** Simulated Spin Hamiltonian parameters for **Cu(II)-*Sm*AA10A** and **Cu(II)-*Sm*AA10A·chitin**	S10
**Figure S2.** EPR spectra of **Cu(II)-*Sm*AA10A** and **Cu(II)-*Sm*AA10A·chitin**	S11
**Figure S3.** XAS damage assessment on a chitin-incubated **Cu(II)-*Sm*AA10A**	S12
**Figure S4.** Subtraction of unbound component from chitin-bound Cu K-edge spectra	S13
**Figure S5.** Cu K-edge spectra for **Cu(II)-*Sm*AA10A** and **Cu(II)-*Sm*AA10A·chitin**	S14
**Figure S6.** k^3-EXAFS data for the Cu(II) samples.	S15
**Figure S7.** Atom labels used for scattering paths in fitting of the EXAFS data	S16
EXAFS data fitting for **Cu(II)-*Sm*AA10A** and **Cu(II)-*Sm*AA10A·chitin**	S17–20
Isolation and validity-testing of **Cu(II)-*Sm*AA10A·chitin** EXAFS data	S21–25
EXAFS data fitting for **Cu(I)-*Sm*AA10A** and **Cu(I)-*Sm*AA10A·chitin**	S26–27

Contents (cont'd)	Page
**Figure S13.** Cu Kβ XES mainlines of **Cu(I)-*Sm*AA10A** and **Cu(I)-*Sm*AA10A·chitin**	S28
**Figure S14.** Modeling background of the VtC region for subtraction	S29
**Figure S15.** Peak fitting analysis of the VtC spectra	S30
**Figure S16, Table S11.** Geometry-optimized cluster models for the *Sm*AA10A LPMO	S31–32
**Figure S17.** Preliminary cluster model for **Cu(I)-*Sm*AA10A·chitin** optimization setup	S33
**Figures S18–19.** NTO and *xyz* polarization component analyses for Cu K-edge XAS of **Cu(II)-*Sm*AA10A** and **Cu(II)-*Sm*AA10A·chitin**	S34
NTO analysis for Cu K-edge XAS of **Cu(I)-*Sm*AA10A** and **Cu(I)-*Sm*AA10A·chitin**	S35–36
Calculated Valence-to-Core Spectra for **Cu(I)-*Sm*AA10A** and **Cu(I)-*Sm*AA10A·chitin**	S37
**Figures S22–23.** MO and *xyz* polarization component analyses for VtC-XES of **Cu(I)-*Sm*AA10A** and **Cu(I)-*Sm*AA10A·chitin**	S38
**Figure S24.** Conformational parameters investigated with histidine brace toy complexes	S39
**Figures S25–27.** Calculated EXAFS, Cu K-edge XAS, and VtC of histidine brace toy complexes	S40–42
**Figure S28, Table 12.** Optimized geometries and calculated binding energies for **Cu(I)-*Sm*AA10A·H_2O_2** and **Cu(I)-*Sm*AA10A·chitin·H_2O_2**	S43
**Figures S29–30.** Optimized geometries and spin density plots for intermediate complexes in H_2O_2 activation by **Cu(I)-*Sm*AA10A·H_2O_2**	S44
**Table S13.** Selected structural metrics from H_2O_2 activation intermediates	S45
**Figures S31–32.** Optimized geometries and spin density plots for intermediate complexes in H_2O_2 activation by **Cu(I)-*Sm*AA10A·chitin·H_2O_2**	S46
**Figure S33.** Overlay of various intermediates of **Cu(I)-*Sm*AA10A·H_2O_2** and **Cu(I)-*Sm*AA10A·chitin·H_2O_2** along reaction pathway	S47
Geometry-optimized coordinates for the *Sm*AA10A LPMO	S48–82
References	S83–85

Additional Experimental Procedures

Protein expression & purification. Protein expression and purification were performed as previously described.[1] In brief, LB- Ampicillin (50 $\mu g \cdot ml^{-1}$) cultures of One Shot™ BL21 Star™ DE3 (*Invitrogen*, Waltham, MA, USA) cells harboring the pRSETB-*cbp21* plasmid were setup by inoculating the media with a glycerol stab prior to 16 hours incubation at 37 ºC with 200 rpm agitation. Due to the leaky nature of the promoter controlling *cbp21* expression, no induction was needed. The cells were harvested, and the periplasmic space was disrupted using a cold osmotic shock method,[2] releasing the periplasmic extract including *Sm*AA10A.

Purification of *Sm*AA10A was performed using a self-packed 15 mL column with a chitin resin (*NEB*, Ipswich, MA, USA) equilibrated with the binding buffer (50 mM Tris-HCl, pH 8.0, 1.5 M NH_4SO_4). The periplasmic extract was adjusted to the same conditions as the binding buffer prior to applying it on the column. Unspecific bound proteins were washed out of the column using 5 column volumes of binding buffer prior to eluting *Sm*AA10A from the column with 20 mM acetic acid. The elution fractions were accessed by SDS-PAGE, and pure fractions (>95%) were collected. We deemed it important, in all samples, to avoid the presence of chloride ions, as previous crystallography and EPR studies on AA9 LPMOs have demonstrated an interaction of chloride with the Cu site, particularly under substrate binding conditions.[3] Furthermore, previous reports of pH effects on the spectroscopic signature of AA10 LPMOs, as well as our own independent investigations on **Cu(II)-*Sm*AA10A**, demonstrate that additional species arise in high pH conditions.[4–6] Therefore, a pH 6.0 MES environment was chosen to circumvent any possible convoluting pH and chloride interaction effects and additionally provide X-ray spectroscopy data directly comparable to those we previously collected on *Nc*AA9C.[7] Therefore, the collected fractions were buffer exchanged to 50 mM MES pH 6.0 using several rounds of concentration and dilution with Amicon® Ultra-15 Centrifugal filters with 10 kDa cut-off (*Merck*, Darmstadt, Germany).

β-chitin sourcing. All chitin-containing experiments described in this work utilized *β*-chitin, which has previously demonstrated the highest affinity for *Sm*AA10A.[8] The *β*-chitin substrate was sourced from squid pen *β*-chitin (*France Chitine*, Orange, France; Batch No. 20140101) and was delivered as long fibrils, which were milled in-house. Substrate fractions < 75 µm were achieved by successive ball milling using a PM 200 planetary ball mill equipped with zirconium oxide milling tools (*Retsch*, Haan, Germany). The milling procedure consisted of a sequence of a 5 minute clockwise and counterclockwise milling step separated by a 2-minute pause to alleviate heat buildup during the initial milling step.

EPR spectroscopy. EPR spectroscopic data were measured directly on the X-ray spectroscopy samples as prepared in Delrin cells. Each Delrin sample cell was affixed to a 4 mm (OD) quartz EPR tube under liquid nitrogen using heat shrink tubing. The continuous-wave X-band (~9.46 GHz) EPR spectra were measured on a Freiberg Instruments Magnettech MS-5000 spectrometer equipped with a temperature controller set to 94 K. Spectra were collected with a field modulation amplitude of 7.500 G at a frequency of 100 kHz and utilizing a microwave power of 0.0500 mW. Spectra were collected with ~24 k points, to which an effective time constant of 0.04 s was applied. A total of at least 6 scans were collected for each sample. Experimental scan parameters are delineated in Table S2. All spectra were processed and simulated in MATLAB 2021b with the *EasySpin* package (v 6.0.0-dev.51, release 2023-01-18),[9] and simulated spin Hamiltonian parameters are listed in Table S3. Spin Hamiltonian parameters for **Cu(II)-*Sm*AA10A·chitin** were determined by simulating the difference spectrum afforded by subtraction of the spectral contribution from the unbound species (Figure S2). For samples which contained a mixture of **Cu(II)-*Sm*AA10A** and **Cu(II)-**

***Sm*AA10A·chitin**, the percent chitin-binding fraction was determined by modeling each of the two Cu(II) sites (using the simulated Spin Hamiltonian parameters in Table S3) and adjusting their relative weight contributions to the overall signal until the simulated signal suitably reproduced the experimental spectrum.

UV-vis Absorbance. UV-vis absorbance spectra were collected on an Agilent Cary 60 UV-vis spectrophotometer. Samples were loaded into a 50-µL Eppendorf cuvette and diluted to a concentration at which the intensity of the peak of interest fell within the linear range of the detector. A scan rate of 80 nm/sec was used to sweep from 800 to 200 nm. Prior to collection on the LPMO sample, an initial spectrum was collected on a blank solution composed of only buffer, which was used for baselining of the final spectrum.

Computational Methods.

Cluster models of *Sm*AA10A were prepared using *PyMOL* (2.5.4, *Schrödinger, LLC*).[10] All Density Functional Theory (DFT) and Time-Dependent Density Functional Theory (TDDFT) calculations were performed using ORCA 5.0.4.[11–15] Localized orbital overlaps and excited-state hole-electron Hirshfeld populations were calculated using Multiwfn.[16,17]

Generation of Cu(II)-containing SmAA10A Cluster Models. Optimized structures for **Cu(II)-*Sm*AA10A** and **Cu(II)-*Sm*AA10A·chitin** have previously been generated as optimized QM/MM models.[8,18] From these reported models, cluster models for this study were generated by the extraction of a subset of atomic coordinates from these existing models to include Cu, water ligands, His28, and the head groups from His114, Phe187, Glu60, and Ala112 (Figure S16, Table S11). His28 was truncated after the Cα, where the carboxylic acid group was replaced by hydrogen, while the N-terminal amino group was unchanged. His114, Phe187, Glu60, and Ala112 were prepared by truncation at the Cα and substitution with hydrogens to form a methyl group at each Cα site, following our previously reported methodology.[7] For the chitin bound **Cu(II)-*Sm*AA10A·chitin**, the cluster model additionally included two *N*-acetylglucosamine (NAG) units (chitobiose) truncated from the *β*-chitin substate structure in the existing model. It was determined that only two NAG units (containing one glycosidic linkage) were enough to adequately reproduce the intensity modulations observed in the experimental spectra, eliminating the necessity for a longer oligomeric chain. The terminal ends of the chitobiose were capped with methyl groups, which were then kept fixed in space. These Cu(II) models were used as inputs for TDDFT-calculated X-ray absorption spectra and DFT-calculated X-ray emission spectra.

Generation of Cu(I)-containing SmAA10A Cluster Models. The cluster models for **Cu(I)-*Sm*AA10A** and **Cu(I)-*Sm*AA10A·chitin** (Figure S16, Table S11) were prepared using coordinates from the previously-reported XRD crystal structure for *Sm*AA10A available in the *Protein Data Bank* (PDB ID: 2BEM, resolution 1.55 Å, Chain C).[1] The PDB crystal structure was reported with a bound Na rather than Cu. Thus, the Na atom was replaced with a Cu atom, and hydrogen assignments for all structures were performed using the Molprobity webserver,[19] and the protonation states were reconfirmed based on pK_a values at pH 6.0 using PROPKA3.[20] The amino acid residues were prepared as described for the Cu(II) models. To mimic the constraints imposed on the active site by the full protein, including bonds to the backbone and strong hydrogen bonds, Cα atoms (Cβ for His28) were fixed in position using a fragment constraint (Keyword: ConnectFragments) protocol during optimizations. Each amino acid and Cu were considered as a separate fragment, and fragments were connected through the following atoms: Cβ of His28, Cα of

His114, Cα of Phe187, Cα of Glu60, Cα of Ala112 and Cu. For **Cu(I)-*Sm*AA10A·chitin**, the NAG fragments were extracted from the Cu(II) substrate-bound QM/MM model and aligned with the Cu(I) free protein cluster model keeping the same Cu-chitin distance (as in Cu(II) substrate bound state), i.e. same Cu-C1(H) and Cu-C4(H) distances. Since the Cu(I) models had to be optimized, each NAG unit was considered as a separate fragment and fragments were connected through the following atoms: Cα of His28, Cα of His114, Cα of Phe187, Cα of Glu60, Cα of Ala112, Cu, glycosyl ring C2 atom bound to the *N*-acetyl group of NAG fragment 1, and C3 atom in NAG fragment 2. This was done to avoid torsional strain on the two NAG units. Lastly, the two NAG fragments 1 and 2 were interconnected to each other by connecting the carbon atoms of the glycosidic bond to prevent dissociation of individual NAG fragments (see Figure S17). The Cu(I) models were geometry-optimized by DFT calculations as described below, and the results used as inputs in TDDFT and DFT calculations. In the case of **Cu(I)-*Sm*AA10A·$\mathbf{H_2O_2}$** and **Cu(I)-*Sm*AA10A·chitin·$\mathbf{H_2O_2}$** models, H_2O_2 was added in the respective Cu(I) optimized cluster models with two H-bonds to Glu60 based on prior studies[18], followed by DFT optimizations as described below.

Geometry optimization. Geometry optimizations were performed using the GGA functional BP86[21,22] and the def2-TZVPP[23] basis set for Cu while using the def2-TZVP[23] basis set for all other atoms in the cluster model. Similarly to reported previously,[7] the def2/J[24] basis sets along with the chain of spheres approximation (RIJCOSX)[25] were used for the RI (resolution of identity) approximation to the Coulomb integrals. The calculations were performed using water implicit solvation as per the control of the Conductor-like Polarizable Continuum Model (CPCM)[26,27] approach and using atom-pairwise dispersion correction based on tight binding partial charges (D4).[28,29] The optimization convergence criteria were set to "TightOpt", while the SCF convergence criteria were set with "TightSCF" keyword. The default integration grid (Def2grid) was employed. A sample input for geometry optimization is provided below.

Generation of Histidine Brace Toy Complexes. The optimized cluster model structure for **Cu(I)-*Sm*AA10A** was used as a starting structure to prepare a series of Cu(I) His-brace "toy" models. Four conformational parameters (Figure S24) were iteratively varied, and the resulting generated structures used for FEFF10 (EXAFS), TDDFT (K-edge), and DFT (VtC-XES) calculations (see below). First, the Cu–N_{term} distance, between the Cu ion and the terminal amine N from His28, was varied from 2.19 to 2.21 Å in 0.002 Å increments. The second parameter investigated (θ_T) is defined as the angle between the Cu–$N^{\varepsilon 2}_{His114}$ bond and the histidine brace plane defined by Cu, $N^{\delta 1}_{His28}$, and N_{term}, and reflects the planarity of the T-shaped brace.[30,31] The θ_T value was varied from 0° to 24° in 3° increments by adjustments of the $N^{\delta 1}_{His28}$–N_{term}–Cu–$N^{\varepsilon 2}_{His114}$ torsional angle. The third parameter (θ_3) is defined as the $N^{\varepsilon 2}_{His114}$–Cu–$N^{\delta 1}_{His28}$ angle (analogous to a tridentate ligand bite angle) and was varied from 180° to 145° in 5° increments. The fourth parameter (θ_D) describes the dihedral angle between the imidazole ring planes. Structures were generated by rotating the His114 atoms around the Cu–$N^{\varepsilon 2}_{His114}$ bond axis in 15° increments. The θ_D for each structure was determined by defining the two least-squared planes from the non-hydrogen atoms of each imidazole ring and calculating the resulting dihedral angle.

FEFF10-Calculated EXAFS. FEFF-calculated EXAFS data were obtained by using the *FEFF10* code.[32] An amplitude reduction factor of 1.0 was applied in the calculations (S02 1.0). Scattering paths were calculated at distances up to 5 Å (RPATH 5), and a maximum of three legs was permitted (NLEG 3). The Random Phase Approximation was applied for core-hole effects (COREHOLE RPA), and the Debye-Waller factor was modeled at 15 K using a Correlated Debye (CD) model with a Debye temperature of

1000 K (DEBYE 15 1000 0). We have previously demonstrated that, for mono- and multi-metallic molecular systems, a Debye temperature of 1000 K provides reasonable results while circumventing the need for expensive DFT calculations.[33] Hydrogen atoms were excluded from calculations due to the weak scattering contribution of hydrogen atoms to the EXAFS signal.

Binding energies. H_2O_2 binding energies were calculated as $\Delta E = E_{AB} - (E_A + E_B)$, where E_{AB}, E_A, and E_B are the total electronic energies of the optimized H_2O_2 bound systems, the H_2O_2 unbound systems, and H_2O_2 (with implicit solvation), respectively.

Sample input for geometry optimization

```
!UKS BP86 TIGHTOPT TIGHTSCF def2-TZVP DEF2/J D4 CPCM(Water) SlowConv LargePrint

%basis newGTO Cu "def2-TZVPP" end
end

%pal nprocs 8
end

%geom
  ConnectFragments
  {1 2 C 2 17 }
  {1 3 C 2 30 }
  {1 4 C 2 38 }
  {1 5 C 2 53 }
  {1 6 O 2 71 }
  end
end

%output
Print[ P_Basis ] 2
Print[ P_MOs ] 1
Print[P_ReducedOrbPopMO_L] 1
Print[ P_Hirshfeld] 1
end

* xyz 0 1
```

Sample input for TDDFT K-edge XAS

```
!RKS RIJCOSX CAM-B3LYP ZORA ZORA-def2-TZVP AUTOAUX
!SlowConv TightSCF LargePrint CPCM(water)

%basis newGTO Cu "ZORA-def2-TZVPP" end
end
%Maxcore 3000
%pal nprocs 12
end

%scf
MaxIter 700
end

%tddft
orbwin[0]= 0,0,-1,-1
doquad true
nroots 300
maxdim 50
DoNTO true
NTOThresh 1e-4
NTOStates 1,2,3,4,5
end

%output
Print[ P_Basis ] 2
Print[ P_MOs ] 1
Print[P_ReducedOrbPopMO_L] 1
Print[ P_Hirshfeld] 1
end

*xyzfile 0 1 finame.xyz
```

Table S1. Incubation conditions tested for optimizing chitin binding to **Cu(II)-*Sm*AA10A**. Each reaction was performed in 50 mM MES, pH 6.0, using 80 mg *β*-chitin in a 2-mL Eppendorf tube, and incubated for two hours. The resulting slurry was transferred into a Delrin cell, sealed with Kapton tape, frozen in liquid nitrogen, and the X-band EPR was then measured at 94 K. The EPR data was simulated using PCA with components derived from the spin Hamiltonian parameters delineated in Table S3 in order to determine the relative amounts of chitin-bound vs solution state species in the sample.

Trial	Volume of Protein added	Protein concentration	Temperature	Chitin Pre-hydration	% chitin-bound
1	600 µL	2 mM	4 °C	Yes, 300 µL	33 %
2	100 µL	2 mM	r.t.	Yes, 300 µL	60 %
3	300 µL	2 mM	r.t	Yes, 300 µL	55 %
4	600 µL	2 mM	r.t	Yes, 300 µL	38 %
5	600 µL	1 mM	r.t.	Yes, 300 µL	55 %
6	600 µL	0.33 mM	r.t.	Yes, 300 µL	70 %
7	100 µL	2 mM	40 °C	Yes, 300 µL	85 %
8	400 µL	2 mM	40 °C	No	75 %

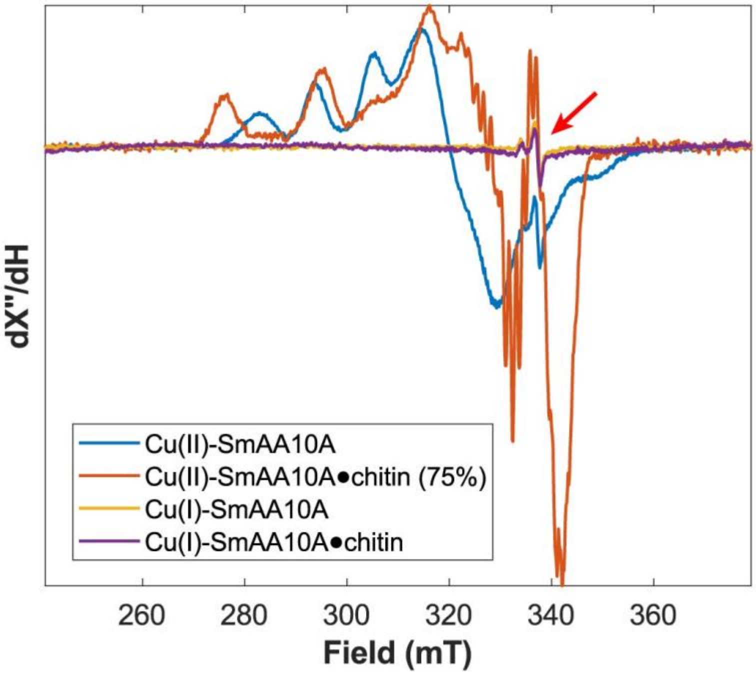

Figure S1. Background-subtracted X-band CW EPR spectra of sample cells collected prior to X-ray experiments. For **Cu(II)-*Sm*AA10A·chitin**, only the 75% chitin-bound sample (Trial 8 in Table S1) is shown. No normalization has been applied to the data. Note that the contribution to the signal at ~335 mT (indicated with a red arrow) is attributed to the Delrin polymer used to manufacture the X-ray cell. The signal originates from an organic radical and does not affect the X-ray spectroscopic data.

Table S2. X-band continuous wave (CW) EPR scan parameters for the Cu(II) samples. Similar scan parameters were used for all samples in this study.

Microwave Freq (GHz)	9.46
Power (mW)	0.0500
Field sweep (mT)	240 to 380
Sweep time (s)	100
Modulation amplitude (mT)	0.7500
Modulation Frequency (kHz)	100
Number of points	~24400
Effective time constant (ms)	0.04
Accumulations	6
Temperature (K)	94

Table S3. Spin Hamiltonian parameters for the Cu(II)-*Sm*AA10A samples without and with chitin. The parameters were determined by simulation in *MATLAB* 2021b with the *EasySpin* package (v 6.0.0-dev.43, release 2022-08-18).[9]

	**$[g_x, g_y, g_z]$**	**A_{Cu} $[A_x, A_y, A_z]$ ($\times 10^{-4}$ cm^{-1})** [a]	**A_N $[A_x, A_y, A_z]$ ($\times 10^{-4}$ cm^{-1})** [a]
**Cu(II)-*Sm*AA10A**	[2.023, 2.120, 2.263]	[73, 38, 112]	
**Cu(II)-*Sm*AA10A·chitin** [b]	[2.045, 2.065, 2.210]	[12, 8.3, 203]	[11, 11, 16.6]<br>[13, 9, 9.6]<br>[9.6, 12.6, 10]

[a] to convert to MHz, multiply by 29979.2458 × 10^{-4}

[b] data were simulated following subtraction of the solution-state species from the observed spectrum

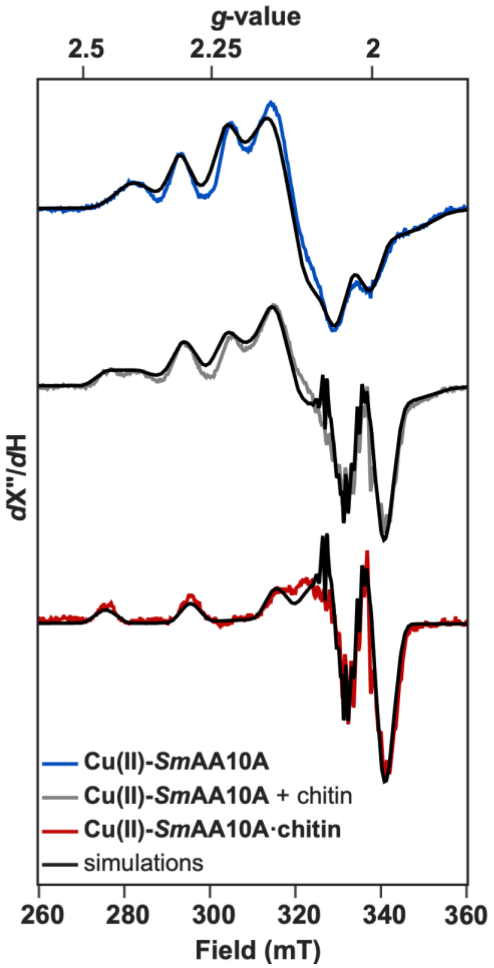

Figure S2. X-band CW EPR of **Cu(II)-*Sm*AA10A** (blue), **Cu(II)-*Sm*AA10A** following treatment with *β*-chitin (grey), and **Cu(II)-*Sm*AA10A·chitin**, obtained by subtraction of unbound species (red). The spectra are max-min normalized. The simulation for each spectrum is shown in black, overlaying the experimental data. Spin Hamiltonian parameters for the simulations are provided in Table S3. Principle component analysis (PCA) of the EPR spectrum before subtraction of the solution state species indicated that only ~33% of the spectral contribution derived from chitin-bound **Cu(II)-*Sm*AA10A·chitin** and that the sample composition primarily featured **Cu(II)-*Sm*AA10A** in the solution state.

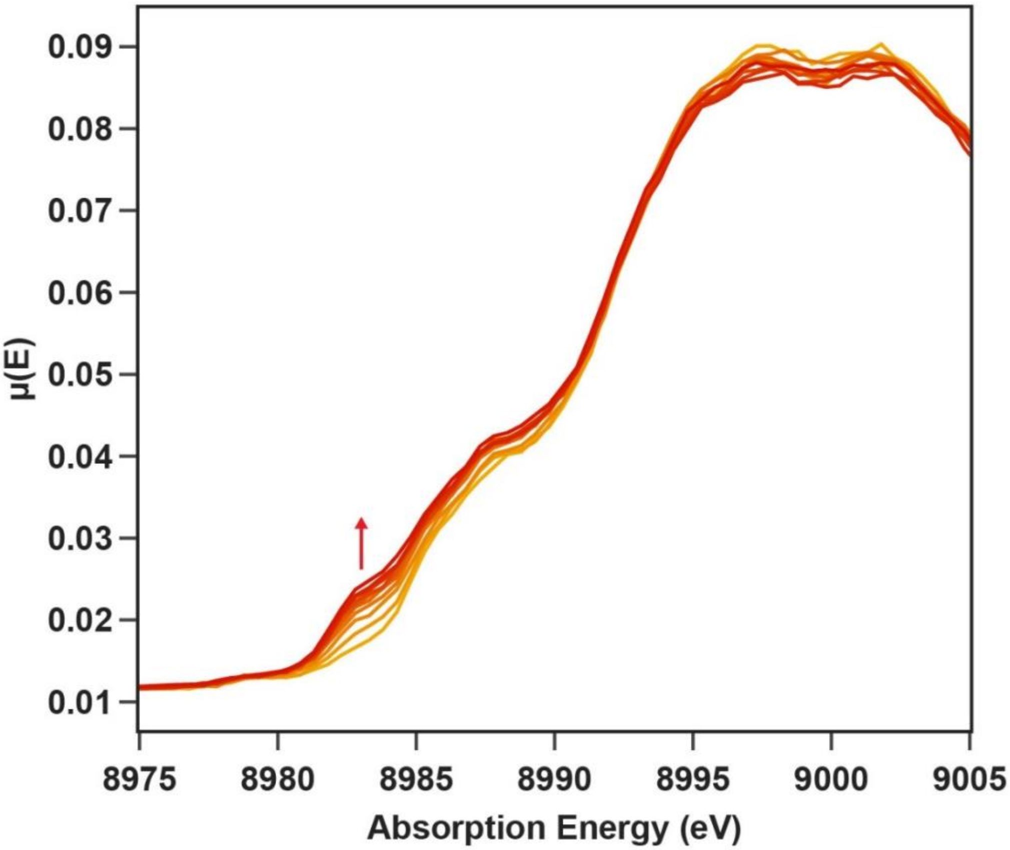

Figure S3. A series of short, edge-region XAS scans obtained during damage assessment of a chitin-incubated **Cu(II)-*Sm*AA10A** (33% chitin-bound), revealing the emergence of a photoinduced feature at 8983.5 eV, attributed to Cu(II)→Cu(I) reduction. The X-ray beam was attenuated to utilize 15% of the total available flux. All scans were taken on the same sample spot, with each scan lasting approximately 90 seconds. The first scan is depicted in yellow-orange and the tenth scan in red. The red arrow indicates the direction of increasing scan number. Spectra are normalized by I_0 (incident flux).

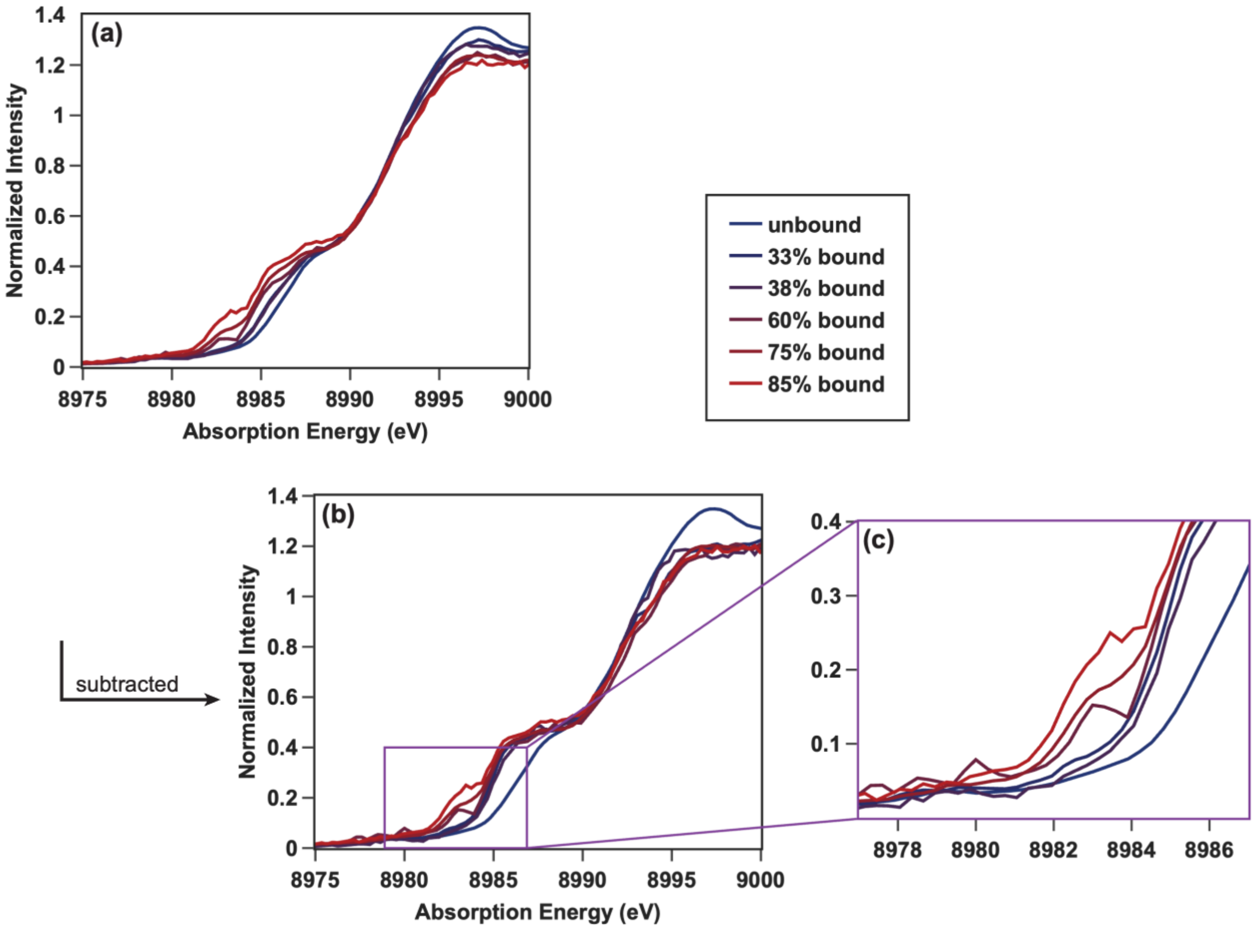

Figure S4. Cu K-edge X-ray absorption data for **Cu(II)-*Sm*AA10A** in solution state (no chitin / "unbound") or as a mixture of chitin-bound and solution-state species with increasing relative chitin-bound component. The data are shown (a) before subtraction of the unbound (solution state) component and (b) after the unbound component has been subtracted from each spectrum. The zoom region (c) depicts the region associated with the X-ray photoinduced feature at 8983.5 eV.

Remarks: Only the feature at 8983.5 eV varies significantly between samples and was therefore determined to be indeed attributable to X-ray induced photodamage. In addition to its position being characteristic for photoreduction of LPMO (see Figure S3),[7,34] the peak intensity is found to be dependent upon the sample composition. Specifically, increasing the chitin-bound component of the sample composition results in increased intensity at 8983.5 eV, suggesting that the chitin-bound **Cu(II)-*Sm*AA10A·chitin** displays increased X-ray photosensitivity relative to the unbound species. Likely, the presence of increased relative amounts of unbound **Cu(II)-*Sm*AA10A** in the lower percentage chitin-bound samples provide a screening effect and therefore diminish the extent of photodamage observed. As such, the data collected from the 33% chitin-bound sample was deemed most representative of the undamaged Cu K-edge spectrum and is used for further analysis in the main paper (Figure 3).

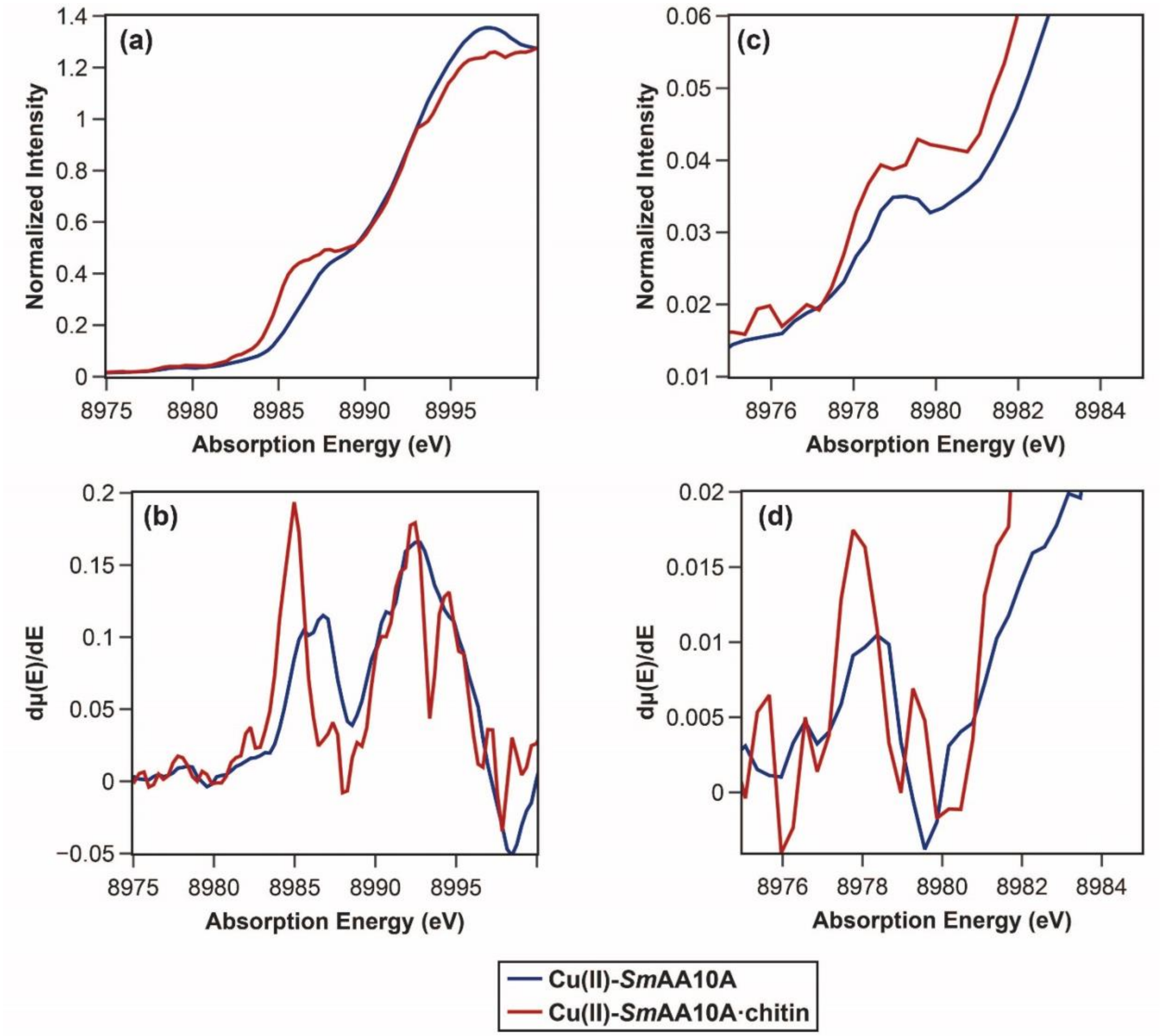

Figure S5. Cu K-edge XAS data for **Cu(II)-*Sm*AA10A** (blue) and **Cu(II)-*Sm*AA10A·chitin** following subtraction of spectral contributions from the unbound species (red). Data for the chitin-bound spectrum is derived from the 33% bound sample (following subtraction). Shown here are (a) the normalized edge region and (b) the first derivative of the edge region followed by (c) the zoomed-in normalized pre-edge region and (d) its first derivative spectrum. No smoothing has been applied to these data.

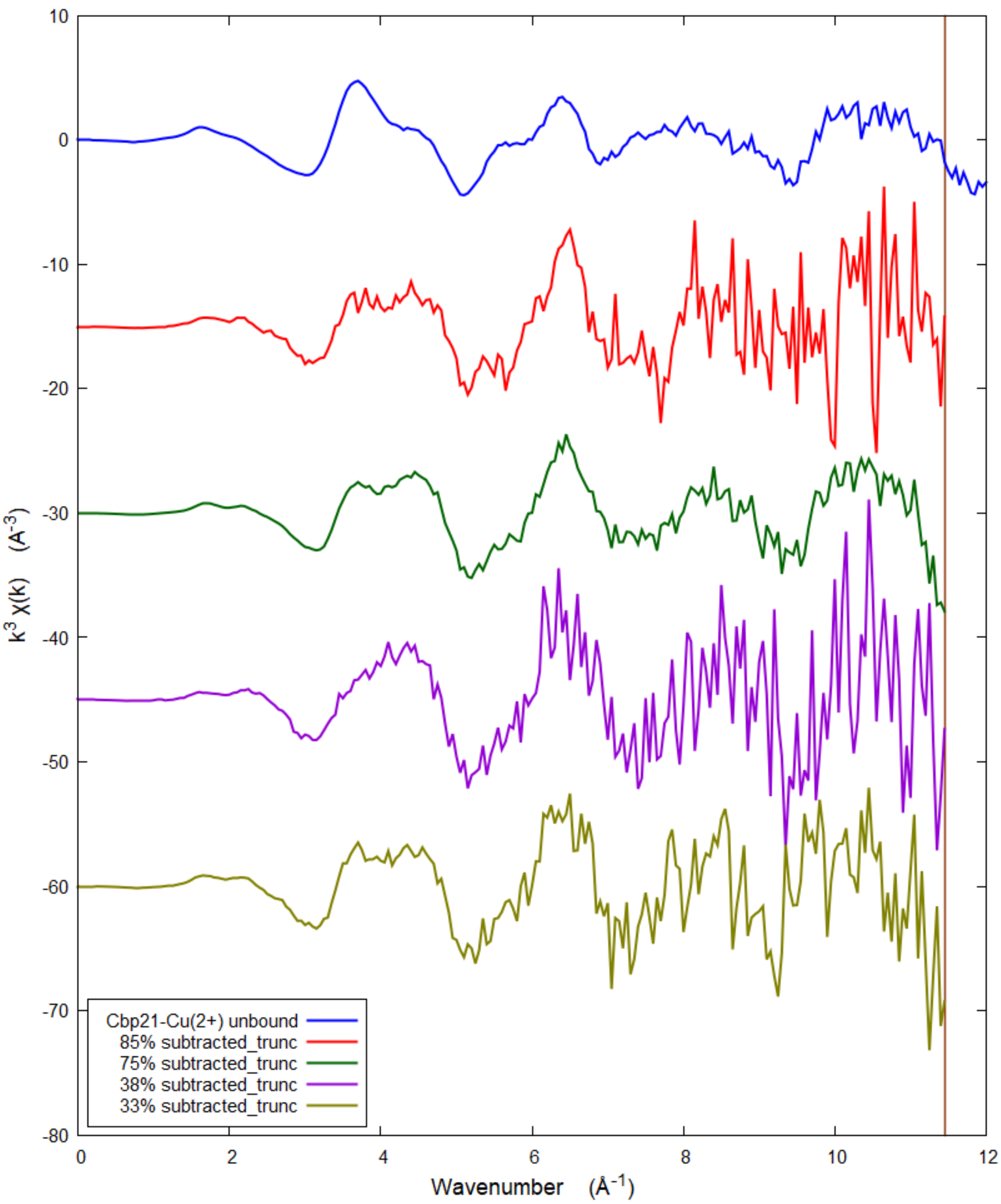

Figure S6. Cu K-edge k^3-weighted EXAFS data for the various Cu(II)-*Sm*AA10A samples collected for this study. The data shown for the chitin-containing samples have been processed following subtraction of the solution state component from the data. From the available chitin-bound data, the 75% bound provides the best signal-to-noise ratio. As such, the data collected from the 75% chitin-bound sample was deemed most suitable for data modeling **Cu(II)-*Sm*AA10A·chitin**.

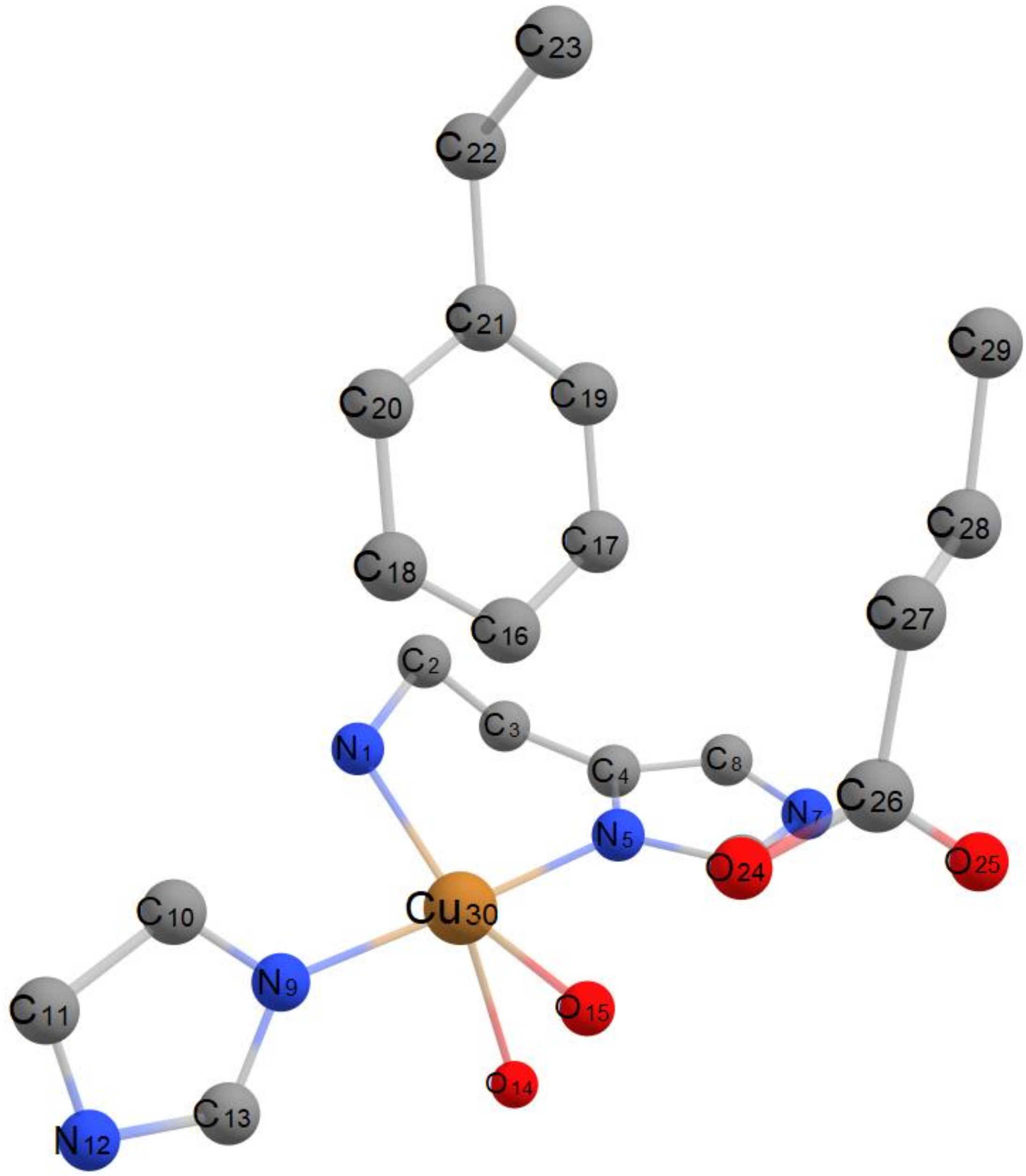

Figure S7. Model and atom labels used for scattering paths in fitting of the EXAFS data for **Cu(II)-*Sm*AA10A** delineated in Table S4. Similar numbering schemes were used for all EXAFS fitting reported in this work. Scattering paths were calculated using FEFF6 and fit to the Fourier Transformed (FT)-EXAFS data, with similar scattering paths grouped together as degenerate when within the resolution (ΔR) determined by the *k*-range of the FT: (a) **Cu(II)-*Sm*AA10A** k = 2–12.5 Å^{-1} (ΔR = 0.150 Å), (b) **Cu(II)-*Sm*AA10A·chitin** k = 2–11 Å^{-1} (ΔR = 0.174 Å), (c) **Cu(I)-*Sm*AA10A** k = 2–12 Å^{-1} (ΔR = 0.157 Å, see ref. [35]), and (d) **Cu(I)-*Sm*AA10A·chitin** k = 2–11.1 Å^{-1} (ΔR = 0.172 Å).

Table S4. Selected EXAFS fitting parameters for **Cu(II)-*Sm*AA10A**. *N* is the path degeneracy, *R* describes the path distance, σ^2 is the Debye-Waller factor, and E_0 is the origin of the photoelectron wave vector. Atom labels are illustrated in Figure S7. Note that the assigned atom numbering only conveys an interpretation of the scattering path. Selected fits are depicted in Figure S8. Scattering paths were calculated using FEFF6 and fit to the FT-EXAFS data, with similar scattering paths grouped together as degenerate when within the ΔR = 0.150 Å resolution limit.

Fit	Path	N	R (Å)	σ^2 ($\times10^{-3}$ Å^2)	E_0 (eV)	red χ^2 *R*-factor
**Fit 1**	Cu-$N_{1/5/9}$	3	1.98	4.66	8990.6	5.324
	Cu-$O_{14/15}$	2	2.14	10.22		0.032
	Cu-$C_{4/6/10/13}$	4	2.98	7.00		
	Cu-C_{16}	1	3.26	–0.02		
	Cu···$N_{5/9}$···$C_{8/11}$/$N_{7/12}$	8	4.16	6.98[a]		
**Fit 2**	Cu-$N_{1/5/9}$	3	1.97	4.75	8990.1	7.648
	Cu-$O_{14/15}$	2	2.13	10.38		0.052
	Cu-$C_{4/6/10/13}$	4	2.97	6.19		
	Cu···$N_{5/9}$···$C_{8/11}$/$N_{7/12}$	8	4.15	7.13[a]		
**Fit 3**	Cu-$N_{1/5/9}$	3	1.98	4.56	8990.9	4.895
	Cu-$O_{14/15}$	2	2.14	9.81		0.029
	Cu-$C_{4/6/10/13}$	4	2.98	5.97		
	Cu-C_{16}	2	3.26	3.97		
	Cu···$N_{5/9}$···$C_{8/11}$/$N_{7/12}$	8	4.16	6.85[a]		
**Fit 4**	Cu-$N_{1/5/9}$	3	1.98	5.02	8990.4	2.693
	Cu-$O_{14/15}$	2	2.13	11.14		0.015
	Cu-$C_{4/6/10/13}$	4	2.98	6.06		
	Cu-C_{16}	2	3.26	3.55		
	Cu···$N_{5/9}$···$C_{8/11}$/$N_{7/12}$	8	4.16	7.54[a]		
	Cu···$C_{4/6/10/13}$···$C_{8/11}$/$N_{7/12}$	8	4.20	9.10[a]		
**Fit 5**	Cu-$N_{1/5/9}$	3	1.98	4.35	8990.3	3.088
	Cu-O_{14}	1	2.14	4.13		0.0017
	Cu-$C_{4/6/10/13}$	4	2.98	6.42		
	Cu-C_{16}	2	3.26	3.90		
	Cu···$N_{5/9}$···$C_{8/11}$/$N_{7/12}$	8	4.16	6.52[a]		
	Cu···$C_{4/6/10/13}$···$C_{8/11}$/$N_{7/12}$	8	4.19	9.63[a]		
**Fit 6**	Cu-$N_{5/9}$	2	1.97	4.01	8990.0	2.505
	Cu-N_1/$O_{14/15}$	3	2.08	17.40		0.0014
	Cu-$C_{4/6/10/13}$	4	2.98	6.46		
	Cu-C_{16}	2	3.26	3.16		
	Cu···$N_{5/9}$···$C_{8/11}$/$N_{7/12}$	8	4.16	6.01[a]		
	Cu···$C_{4/6/10/13}$···$C_{8/11}$/$N_{7/12}$	8	4.17	9.69[a]		
**Fit 7**	Cu-$N_{1/5/9}$	2	1.99	5.84	8990.1	11.95
	Cu-$C_{4/6/10/13}$	4	2.96	6.20		0.076
	Cu-C_{16}	2	3.26	4.17		
	Cu···$N_{5/9}$···$C_{8/11}$/$N_{7/12}$	8	4.15	8.75[a]		
	Cu···$C_{4/6/10/13}$···$C_{8/11}$/$N_{7/12}$	8	4.18	9.30[a]		

[a] σ^2 is defined as 1.5 times that of the single scattering path involving the first scattering atom.

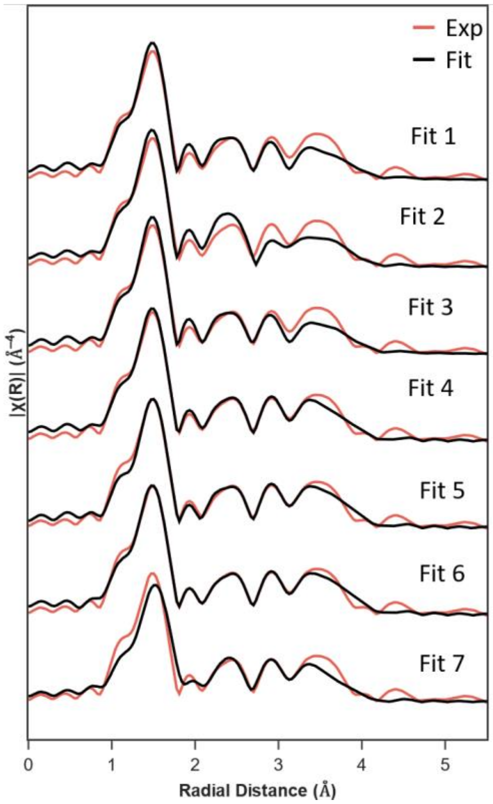

Figure S8. Selected EXAFS fits for **Cu(II)-*Sm*AA10A**. Fitting parameters are delineated in Table S4.

Further remarks on FT-EXAFS fits of Cu(II)-*Sm*AA10A. Initial fitting attempts utilizing only single scattering paths determined that the region of R = 1–3.2 Å could be adequately simulated considering a model consistent with 2 imidazole ligands, a ligating terminal amine, and either one or two water ligands. Attempts to utilize only one unique multiple scattering path with a degeneracy N = 8 (Fits 1–3) were inadequate for simulation of the full data, necessitating a second multiple scattering path. The data suggests that modeling with two water ligands (Fits 4 and 6) does provide a better fit than a model with one water ligand (Fit 5), albeit only slightly. However, either one or two water ligands provides a far superior fit over omission of water ligands entirely (Fit 7). Fits 4 and 6 attempt to determine the grouping of the Cu-N single scattering path from the terminal amine nitrogen (N_1) into either the 1st shell (with imidazole N) or the 2nd shell (water O). Neither fit is significantly superior over the other, with the exception that the exceptionally large Debye-Waller factor which emerges in Fit 6 for the Cu-N_1/$O_{14/15}$ scattering path does undermine the fit. The DFT-optimized structure for **Cu(II)-*Sm*AA10A** suggests a Cu–N_1 distance which is intermediate to the Cu–$N_{imidazole}$ and Cu–O_{water} distances (see main text), likely convoluting the fitting. As a result, characterizing the Cu-N_1 scattering path as degenerate with either group likely contributes to the inflated σ^2 value. However, the 0.150 Å resolution of the EXAFS does not justify introduction of a unique Cu-N_1 path.

Table S5. Selected EXAFS fitting parameters for **Cu(II)-*Sm*AA10A·chitin**. The data from the 75% chitin-bound sample was selected for EXAFS fitting (see *Experimental Procedures, FEFF fitting of FT-EXAFS* in the main text). The contribution from the chitin-unbound **Cu(II)-*Sm*AA10A** was subtracted from the data before processing and fitting. *N* is the path degeneracy, *R* describes the path distance, σ^2 is the Debye-Waller factor, and E_0 is the origin of the photoelectron wave vector. Atom numbering is similar to that illustrated in Figure S7. Note that the assigned atom numbering only conveys an interpretation of the scattering path. Selected fits are depicted in Figure S9. Scattering paths were calculated using FEFF6 and fit to the FT-EXAFS data, with similar scattering paths grouped together as degenerate when within the ΔR = 0.174 Å resolution limit.

Fit	Path	N	R (Å)	σ^2 ($\times10^{-3}$ Å^2)	E_0 (eV)	red χ^2 / *R*-factor
**Fit 1**	$Cu\text{-}N_{1/5/9}$	3	1.96	2.71	8991.1	8.804
	$Cu\text{-}O_{14}$	1	2.46	10.61		0.042
	$Cu\text{-}C_{2/4/6/10/13}$	5	3.00	17.24		
	$Cu\cdots N_{5/9}\cdots C_{8/11}/N_{7/12}$	8	4.16	4.06[a]		
	$Cu\cdots C_{4/6/10/13}\cdots C_{8/11}/N_{7/12}$	8	4.39	25.86[a]		
**Fit 2**	$Cu\text{-}N_{1/5/9}$	3	1.96	2.72	8991.7	7.546
	$Cu\text{-}O_{14}$	1	2.47	12.69		0.031
	$Cu\text{-}C_{2/4/6/10/13}$	5	3.01	11.39		
	$Cu\text{-}C_{16}$	2	3.28	3.10		
	$Cu\cdots N_{5/9}\cdots C_{8/11}/N_{7/12}$	8	4.17	4.08[a]		
**Fit 3**	$Cu\text{-}N_{1/5/9}/O_{14}$	4	1.96	4.45	8991.4	2.678
	$Cu\text{-}C_{2/4/6/10/13}$	5	3.03	9.81		0.014
	$Cu\text{-}C_{16}$	2	3.28	1.59		
	$Cu\cdots N_{5/9}\cdots C_{8/11}/N_{7/12}$	8	4.17	6.68[a]		
**Fit 4**	$Cu\text{-}N_{1/5/9}/O_{14}$	4	1.96	4.44	8991.2	3.069
	$Cu\text{-}C_{2/4/6/10/13}$	5	3.04	11.81		0.016
	$Cu\text{-}C_{16}$	1	3.28	–2.71		
	$Cu\cdots N_{5/9}\cdots C_{8/11}/N_{7/12}$	8	4.17	6.66[a]		
**Fit 5**	$Cu\text{-}N_{1/5/9}/O_{14}$	4	1.96	4.46	8991.3	2.684
	$Cu\text{-}C_{4/6/10/13}$	4	3.02	7.08		0.014
	$Cu\text{-}C_{16}$	2	3.27	2.23		
	$Cu\cdots N_{5/9}\cdots C_{8/11}/N_{7/12}$	8	4.17	6.69[a]		
**Fit 6**	$Cu\text{-}N_{1/5/9}$	3	1.96	2.72	8991.6	7.607
	$Cu\text{-}O_{14}$	1	2.46	12.06		0.032
	$Cu\text{-}C_{4/6/10/13}$	4	3.00	8.36		
	$Cu\text{-}C_{16}$	2	3.27	3.85		
	$Cu\cdots N_{5/9}\cdots C_{8/11}/N_{7/12}$	8	4.17	4.09[a]		
**Fit 7**	$Cu\text{-}N_{1/5/9}$	3	1.96	2.71	8992.3	6.965
	$Cu\text{-}C_{2/4/6/10/13}$	5	3.03	10.10		0.037
	$Cu\text{-}C_{16}$	2	3.28	1.75		
	$Cu\cdots N_{5/9}\cdots C_{8/11}/N_{7/12}$	8	4.18	4.06[a]		

[a] σ^2 is defined as 1.5 times that of the single scattering path involving the first scattering atom.

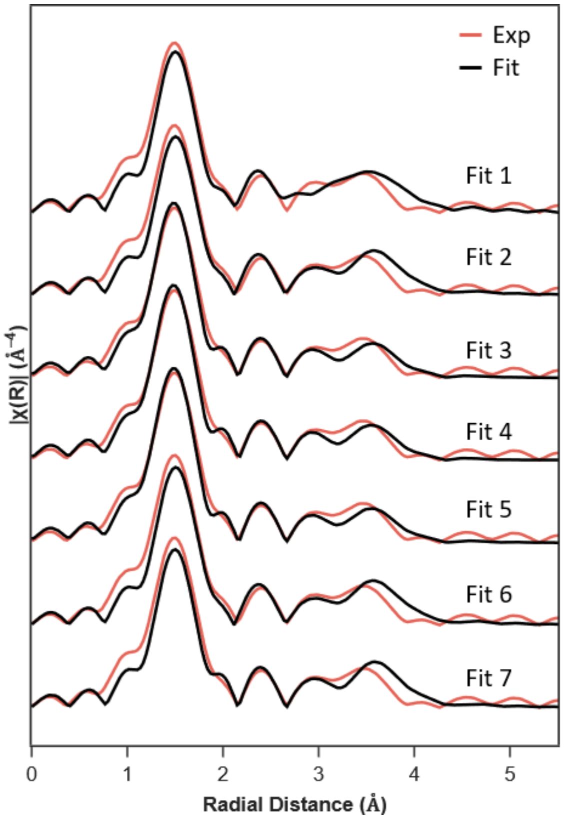

Figure S9. Selected EXAFS fits for **Cu(II)-*Sm*AA10A·chitin**. Fitting parameters are delineated in Table S5.

Further remarks on FT-EXAFS fits of Cu(II)-*Sm*AA10A·chitin. Initial fits of only the single scatterer primary shell paths suggested that the FT-EXAFS could best be fit with a model consistent with one water ligand rather than two or zero, both of which resulted in inflated Debye-Waller factors. Initial simulation of the longer range scattering paths employed paths from only the histidine residues (Fit 1). However, it was found that the inclusion of a single scatterer Cu-C path corresponding to the distance to C_{16} from phenylalanine markedly improved the fits. Interestingly, better fits are obtained with a path degeneracy N = 2 for the Cu-C path (Fits 2–4). The best fits (Fit 3 and Fit 5) grouped together the water ligand with the scattering N atoms from the His ligands (Cu-N/O path, $N = 4$), while separating these paths into two shells (Fit 6) or omitting the water ligand (Fit 7) provided a notably worse fit.

Isolation and validity-testing of Cu(II)-*Sm*AA10A·chitin EXAFS data:

Comparison between the available EXAFS datasets from the chitin-containing samples demonstrates that the 75% chitin-bound sample provides the most superior data quality for interpretation after data subtraction. To demonstrate the validity of the data subtraction, an initial attempt was made to fit two sets of non-subtracted data, collected from the 33% chitin-bound and the 75% chitin-bound samples, as these offered the highest-quality data (Figure S6). The expectation was that these data would fit with models intermediate between those of the **Cu(II)-*Sm*AA10A** and the **Cu(II)-*Sm*AA10A·chitin** EXAFS fits, an assumption which was deduced by qualitative comparison of the two non-subtracted k^3-weighted EXAFS datasets against those collected on the pure **Cu(II)-*Sm*AA10A** sample and the **Cu(II)-*Sm*AA10A·chitin** spectrum determined by data subtraction from the 75% chitin-bound sample (Figure S10). As expected, the fit for the non-subtracted data from the 33% chitin-bound sample (Table S6) more closely resembled that of **Cu(II)-*Sm*AA10A**, with a primary shell scattering path distance of 1.98 Å. Conversely, the fit for the non-subtracted data from the 75% chitin-bound sample (Table S7) was more similar to that of **Cu(II)-*Sm*AA10A·chitin**, with a primary shell scattering path distance of 1.96 Å. In both cases, however, the fitted Debye-Waller values (σ^2) were severely inflated and attempts to alleviate the problem were generally unsuccessful.

As an alternative approach, it has been previously reported that collection of EXAFS data on mixed-species samples affords a spectrum which is a linear combination of the component species.[36] Therefore, Linear Combination Fitting (LCF) analysis of the non-subtracted 33% chitin-bound data, using **Cu(II)-*Sm*AA10A** and **Cu(II)-*Sm*AA10A·chitin** (derived from the 75% chitin-bound data) as the fitting components. The LCF analysis was performed using the *Athena* module within *Demeter*.[37] The weighted components used for the fitting were data from **Cu(II)-*Sm*AA10A** and **Cu(II)-*Sm*AA10A·chitin** (derived from the subtracted 75% chitin-bound data). The EXAFS of all data sets were k^3-weighted and three k-range fitting windows were employed as the fitting space: 3–8 $Å^{-1}$, 3–10 $Å^{-1}$, and 3–11.5 $Å^{-1}$. All component weights were constrained to values between 0 and 1, the sum of weights set to 1, and a least-squares minimization fitting performed. All three LCF analyses showed good agreement with the expected sample composition of 33% chitin-bound species, yielding 36.9%, 36.5%, and 32%, respectively (Table S8, Figure S11).

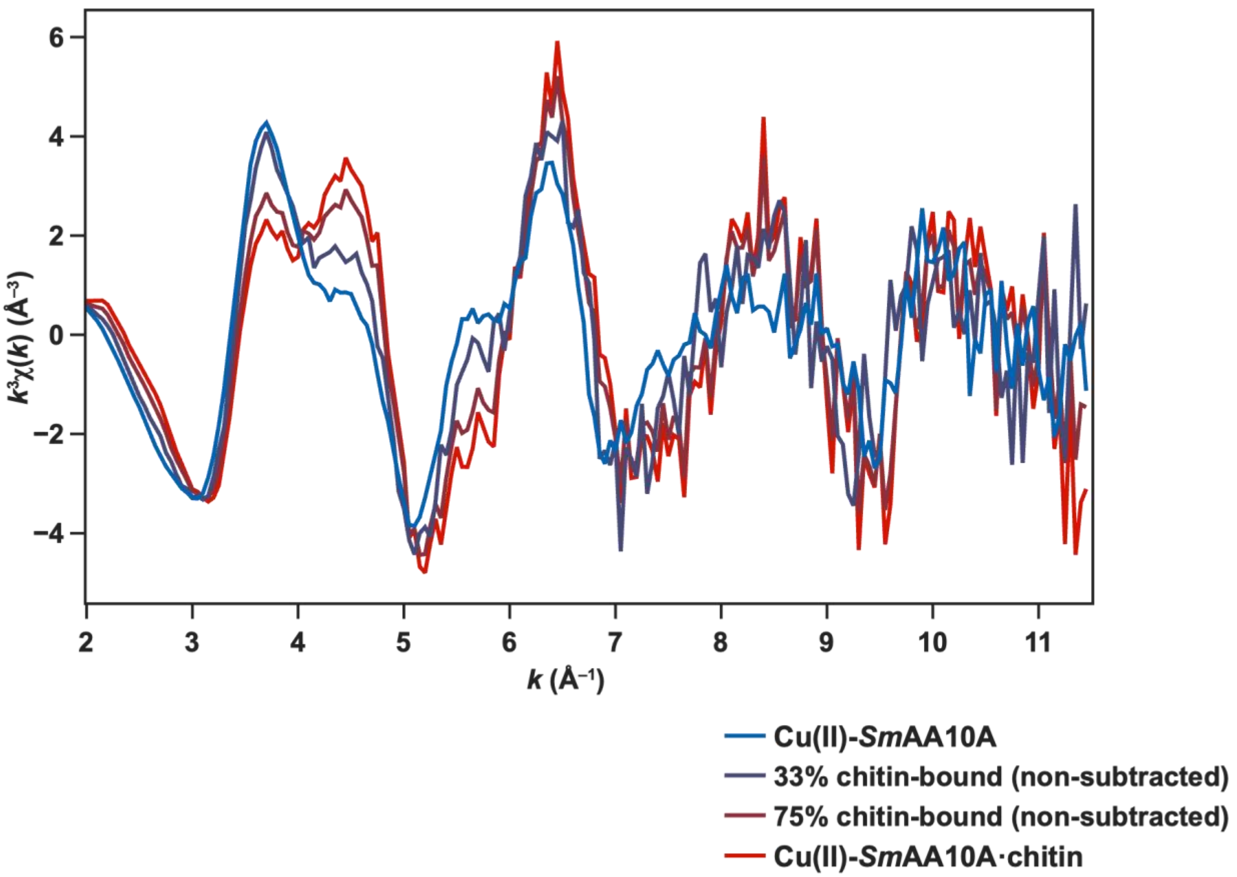

Figure S10. k^3-weighted EXAFS showing the progression of the spectrum from ***Sm*AA10A-Cu(II)** to ***Sm*AA10A-Cu(II)•chitin**, using the 33% chitin-bound and 75% chitin-bound non-subtracted data as intermediate spectra. Data for ***Sm*AA10A-Cu(II)•chitin** is derived from the 75% chitin-bound sample following subtraction of the unbound component.

Table S6. EXAFS fitting parameters for chitin-treated Cu(II)-SmAA10A (33% chitin-bound sample) *without* subtraction of the unbound **Cu(II)-*Sm*AA10A** component. *N* is the path degeneracy, *R* describes the path distance, σ^2 is the Debye-Waller factor, and E_0 is the origin of the photoelectron wave vector. Atom numbering is similar to that illustrated in Figure S7. Note that the assigned atom numbering only conveys an interpretation of the scattering path. Scattering paths were calculated using FEFF6 and fit to the FT-EXAFS data, with similar scattering paths grouped together as degenerate when within the ΔR = 0.169 Å resolution limit (*k* window = 2–11.25 $Å^{-1}$).

Fit	Path	N	R (Å)	σ^2 ($\times 10^{-3}$ $Å^2$)	E_0 (eV)	red χ^2 *R*-factor
**Fit 1**	Cu-$N_{1/5/9}$	3	1.98	6.70	8986.8	13.81
	Cu-$O_{14/15}$	2	1.94	11.53		0.042
	Cu-$C_{4/6/10/13}$	4	2.96	9.57		
	Cu-C_{16}	2	3.31	3.36		
	Cu···$N_{5/9}$···$C_{8/11}$/$N_{7/12}$	8	4.09	10.05[a]		
	Cu···$C_{4/6/10/13}$···$C_{8/11}$/$N_{7/12}$	8	4.17	14.36[a]		
**Fit 2**	Cu-$N_{1/5/9}$	3	1.98	4.85	8990 (locked)	12.59
	Cu-$O_{14/15}$	2	2.04	21.15		0.044
	Cu-$C_{4/6/10/13}$	4	2.99	6.13		
	Cu-C_{16}	2	3.29	4.21		
	Cu···$N_{5/9}$···$C_{8/11}$/$N_{7/12}$	8	4.19	7.27[a]		
	Cu···$C_{4/6/10/13}$···$C_{8/11}$/$N_{7/12}$	8	4.11	9.20[a]		
**Fit 3**	Cu-$N_{1/5/9}$	3	2.00	4.83	8994.2	10.20
	Cu-O_{14}	1	1.48	17.61		0.036
	Cu-$C_{4/6/10/13}$	4	3.03	7.68		
	Cu-C_{16}	2	3.32	2.70		
	Cu···$N_{5/9}$···$C_{8/11}$/$N_{7/12}$	8	4.23	7.24[a]		
**Fit 4**	Cu-$N_{1/5/9}$/O_{14}	4	1.98	6.14	8989.8	10.19
	Cu-$C_{4/6/10/13}$	4	2.99	9.05		0.045
	Cu-C_{16}	2	3.32	3.51		
	Cu···$N_{5/9}$···$C_{8/11}$/$N_{7/12}$	8	4.15	9.22[a]		

[a] σ^2 is defined as 1.5 times that of the single scattering path involving the first scattering atom.

Further remarks on FT-EXAFS fits of non-subtracted chitin-treated Cu(II)-*Sm*AA10A (33% chitin-bound sample). As a starting point, an initial free-floating fit was attempted using the paths from the best fit model for the **Cu(II)-*Sm*AA10A** data (Table S4, Fit 4), yielding a fit with chemically unreasonable path distances for the primary shell, inflated Debye-Waller values, and uncharacteristically low E_0 (Fit 1). Locking the E_0 value at 8990 eV does provide more reasonable scattering path distances (Fit 2), elongating the Cu-$O_{14/15}$ path to 2.04, though the Debye-Waller factor for this path becomes inflated. Initializing the fitting procedure with only one water ligand (similar to fits for the **Cu(II)-*Sm*AA10A·chitin** data (Table S5)) resulted in a severely inflated Debye-Waller value and an unreasonably short Cu-O_{14} scattering path distance when the primary shells were split into two (Fit 3), but converged to lower values upon grouping into one shell (Fit 4). In both fits 3 and 4, the Debye-Waller factor for the Cu-C_{16} path was unreasonably low. In general, however, the 1st shell distance tends to converge toward 1.98 Å, with the exception of Fit 3.

Table S7. EXAFS fitting parameters for chitin-treated Cu(II)-SmAA10A (75% chitin-bound sample) *without* subtraction of the unbound **Cu(II)-*Sm*AA10A** component. *N* is the path degeneracy, *R* describes the path distance, σ^2 is the Debye-Waller factor, and E_0 is the origin of the photoelectron wave vector. Atom numbering is similar to that illustrated in Figure S7. Note that the assigned atom numbering only conveys an interpretation of the scattering path. Scattering paths were calculated using FEFF6 and fit to the FT-EXAFS data, with similar scattering paths grouped together as degenerate when within the ΔR = 0.169 Å resolution limit (*k* window = 2–11.25 $Å^{-1}$).

Fit	Path	N	R (Å)	σ^2 ($\times 10^{-3}$ $Å^2$)	E_0 (eV)	red χ^2 *R*-factor
**Fit 1**	Cu-$N_{1/5/9}$	3	1.96	3.96	8988.3	3.555
	Cu-$O_{14/15}$	2	1.94	22.43		0.011
	Cu-$C_{4/6/10/13}$	4	2.98	6.69		
	Cu-C_{16}	2	3.25	3.31		
	Cu···$N_{5/9}$···$C_{8/11}$/$N_{7/12}$	8	4.15	5.93[a]		
	Cu···$C_{4/6/10/13}$···$C_{8/11}$/$N_{7/12}$	8	4.11	10.03[a]		
**Fit 2**	Cu-$N_{1/5/9}$	3	1.96	3.80	8990	3.745
	Cu-$O_{14/15}$	2	2.00	27.37	(locked)	0.013
	Cu-$C_{4/6/10/13}$	4	3.00	6.00		
	Cu-C_{16}	2	3.25	1.96		
	Cu···$N_{5/9}$···$C_{8/11}$/$N_{7/12}$	8	4.17	5.71[a]		
	Cu···$C_{4/6/10/13}$···$C_{8/11}$/$N_{7/12}$	8	4.13	9.01[a]		
**Fit 3**	Cu-$N_{1/5/9}$	3	1.96	3.48	8990.6	7.471
	Cu-O_{14}	1	2.64	31.61		0.026
	Cu-$C_{4/6/10/13}$	4	2.99	6.24		
	Cu-C_{16}	2	3.25	3.25		
	Cu···$N_{5/9}$···$C_{8/11}$/$N_{7/12}$	8	4.16	5.22[a]		
**Fit 4**	Cu-$N_{1/5/9}$/O_{14}	4	1.96	5.27	8990.1	2.458
	Cu-$C_{4/6/10/13}$	4	3.00	7.10		0.011
	Cu-C_{16}	2	3.26	2.92		
	Cu···$N_{5/9}$···$C_{8/11}$/$N_{7/12}$	8	4.16	7.90[a]		

[a] σ^2 is defined as 1.5 times that of the single scattering path involving the first scattering atom.

Further remarks on FT-EXAFS fits of non-subtracted chitin-treated Cu(II)-*Sm*AA10A (75% chitin-bound sample). As a starting point, an initial free-floating fit was attempted using the paths from the best fit model for the **Cu(II)-*Sm*AA10A** data (Table S4, Fit 4). This converged, however, to wildly inflated Debye-Waller factors and chemically unreasonable path distances for the primary shell and unusually low E_0 (Fit 1). Locking the E_0 value at 8990 eV does provide more reasonable scattering path distances (Fit 2), elongating the Cu-$O_{14/15}$ path to 2.00, though the Debye-Waller factor for this path also increases to an untenable value. Conversely, initializing the fitting procedure with only one water ligand (similar to fits for the **Cu(II)-*Sm*AA10A·chitin** data (Table S5)) resulted in a severely inflated Debye-Waller value when the primary shells were split into two (Fit 3), but converged to lower values upon grouping into one shell (Fit 4). In both fits 3 and 4, the Debye-Waller factor for the Cu-C_{16} path was unreasonably low. In general, however, the 1st shell distance tends to converge toward 1.96 Å.

Table S8. Linear combination fitting analysis of the k^3-weighted EXAFS for the non-subtracted chitin-treated Cu(II)-*Sm*AA10A (33% chitin-bound) sample. Fits are depicted in Figure S11.

**Fitting Window**	***k* 3–8 Å^{-1}**	***k* 3–10 Å^{-1}**	***k* 3–11.5 Å^{-1}**
**Component Weights**			
**Cu(II)-*Sm*AA10A·chitin**	0.369	0.365	0.320
**Cu(II)-*Sm*AA10A**	0.631	0.635	0.680
**red χ^2**	0.39	0.71	0.99
***R*-factor**	0.067	0.144	0.224

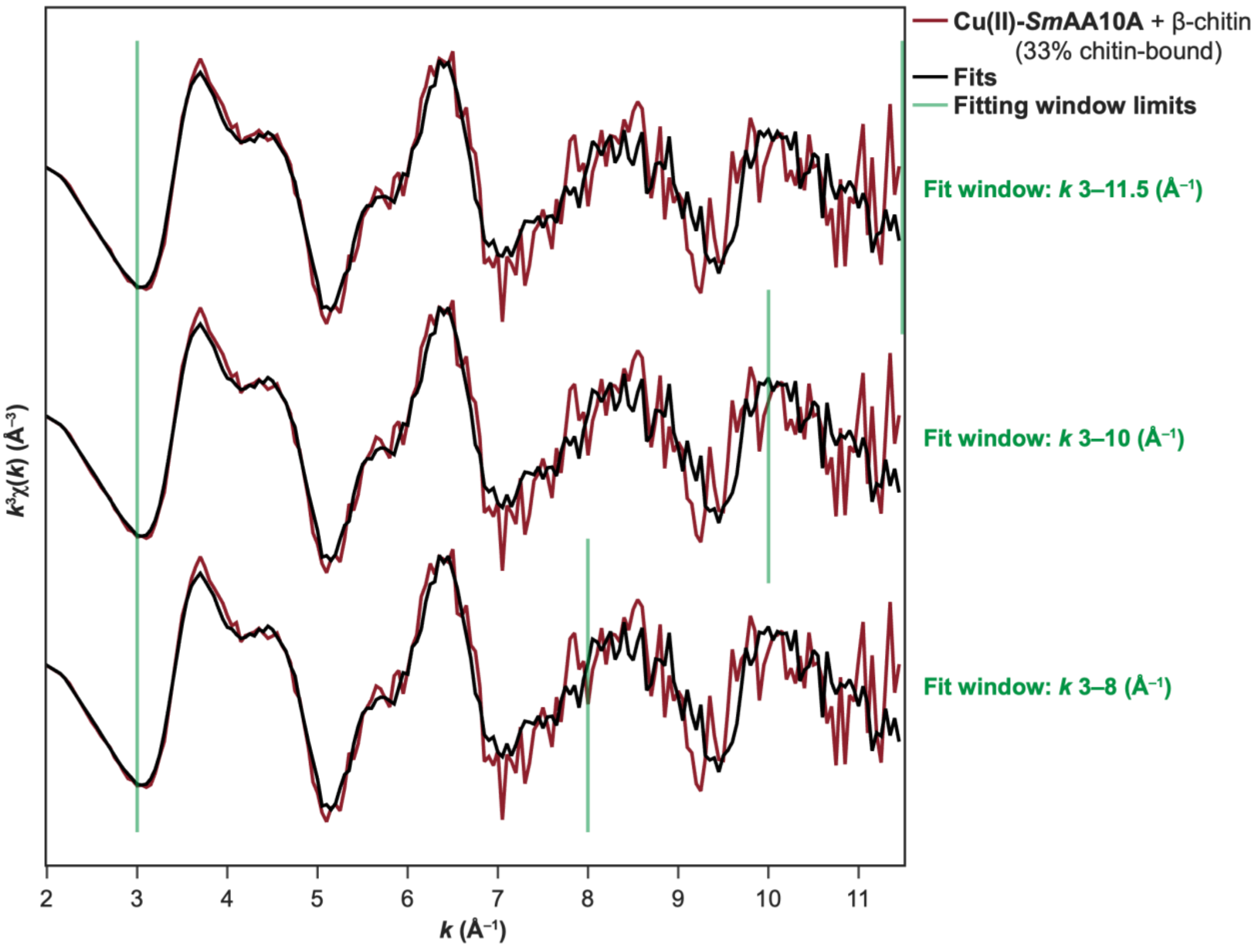

Figure S11. Linear combination fits delineated in Table S8. The fitted experimental k^3-weighted EXAFS data is depicted in red, and the linear combination fits are depicted in black.

Table S9. Best EXAFS fitting parameters for (chitin-free) **Cu(I)-*Sm*AA10A**, previously reported in ref. [35]. *N* is the path degeneracy, *R* describes the path distance, σ^2 is the Debye-Waller factor, and E_0 is the origin of the photoelectron wave vector. Atom numbering is similar to that illustrated in Figure S7. Note that the assigned atom numbering only conveys an interpretation of the scattering path.

**Path**	**N**	**R (Å)**	**σ^2 ($\times10^{-3}$ Å^2)**	**E_0 (eV)**
$Cu-N_{5/9}$	2	1.89	2.77	8987.1
$Cu-N_1$	1	2.21	2.67	
$Cu-C_{4/6/10/13}$	4	2.91	2.77	
$Cu\cdots N_{5/9}\cdots C_{4/6/10/13}$	8	3.08	4.15	
$Cu\cdots N_{5/9}\cdots C_{8/11}/N_{7/12}$	8	4.11	4.15	

Table S10. EXAFS fitting parameters for the **Cu(I)-*Sm*AA10A·chitin** sample. Note that the contribution from the unbound **Cu(I)-*Sm*AA10A** could not be subtracted out from the data prior to fitting. *N* is the path degeneracy, *R* describes the path distance, σ^2 is the Debye-Waller factor, and E_0 is the origin of the photoelectron wave vector. Atom numbering is similar to that illustrated in Figure S7. Note that the assigned atom numbering only conveys an interpretation of the scattering path. The FT-EXAFS data and fit are depicted in Figure S12. Scattering paths were calculated using FEFF6 and fit to the FT-EXAFS data, with similar scattering paths grouped together as degenerate when within the $\Delta R = 0.172$ Å resolution limit.

**Fit**	**Path**	**N**	**R (Å)**	**σ^2 ($\times10^{-3}$ Å^2)**	**E_0 (eV)**	**red χ^2 *R*-factor**
**Fit 1**	$Cu-N_{5/9}$	2	1.89	3.92	8987.4	12.71
	$Cu-N_1$	1	2.22	2.27		0.028
	$Cu-C_{4/6/10/13}$	4	2.93	4.38		
	$Cu-C_{2/3}$	3	3.19	3.74		
	$Cu\cdots N_{5/9}\cdots C_{8/11}/N_{7/12}$	8	4.12	5.87[a]		
**Fit 2**	$Cu-N_{5/9}$	2	1.90	4.05	8988.0	16.96
	$Cu-N_1$	1	2.22	2.15		0.043
	$Cu-C_{4/6/10/13}$	4	2.91	3.54		
	$Cu\cdots N_{5/9}\cdots C_{4/6/10/13}$	8	3.03	6.07[a]		
	$Cu\cdots N_{5/9}\cdots C_{8/11}/N_{7/12}$	8	4.13	6.07[a]		
**Fit 3**	$Cu-N_{5/9}$	2	1.90	3.86	8988.0	17.26
	$Cu-N_1$	1	2.22	2.29		0.044
	$Cu-C_{4/6/10/13}$	4	2.91	3.86[b]		
	$Cu\cdots N_{5/9}\cdots C_{4/6/10/13}$	8	3.03	5.79[a]		
	$Cu\cdots N_{5/9}\cdots C_{8/11}/N_{7/12}$	8	4.13	5.79[a]		

[a] σ^2 is defined as 1.5 times that of the first Cu-N single scattering path ($1.5 \times \sigma^2_{Cu-N5/9}$).
[b] σ^2 restrain to be equal to or greater than that of the $Cu-N_{5/9}$ single scattering path.

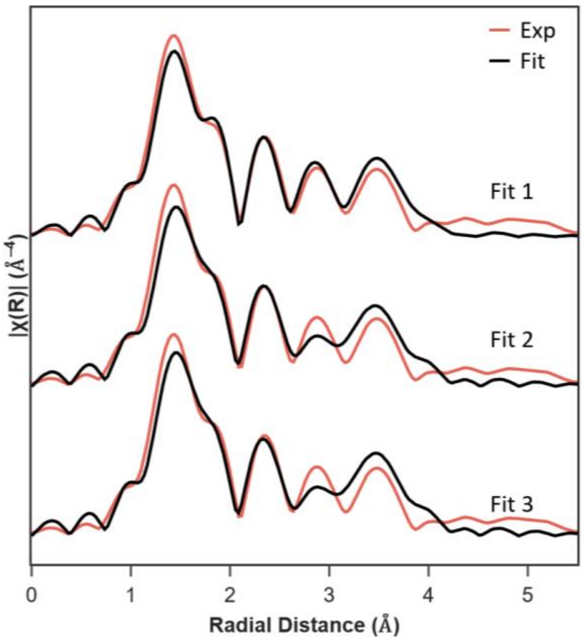

Figure S12. Selected EXAFS fits for the **Cu(I)-*Sm*AA10A·chitin** sample. Fitting parameters are delineated in Table S10.

Further remarks on FT-EXAFS fits of Cu(I)-*Sm*AA10A·chitin. An initial fit which attempted to maximize single scattering paths resulted overall in reasonable path distances (Fit 1). However, the low Debye-Waller factor of the Cu-$C_{2/3}$ path — in particular, the fact that it was lower than that of Cu-$N_{5/9}$ — suggested that another strategy was necessary. Utilization of a Cu···$N_{5/9}$···$C_{4/6/10/13}$ multiple scattering path also yielded reasonable path distances, however leading again to a chemically unreasonable Debye-Waller factors for the single scattering paths (Fit 2). It was nevertheless possible to restrain the Debye-Waller factor of Cu-$C_{4/6/10/13}$ without dramatically altering the fit (Fit 3). In general, however, the fits converged to a model with a 1st shell (N=2) distance of 1.90 Å and a 2nd shell (N=1) distance of 2.22 Å. Notably, the fitted E_0 values in all models suggest a slight shift to higher energy relative to that observed in **Cu(I)-*Sm*AA10A**, which is consistent with the trends in the K-edge spectra.

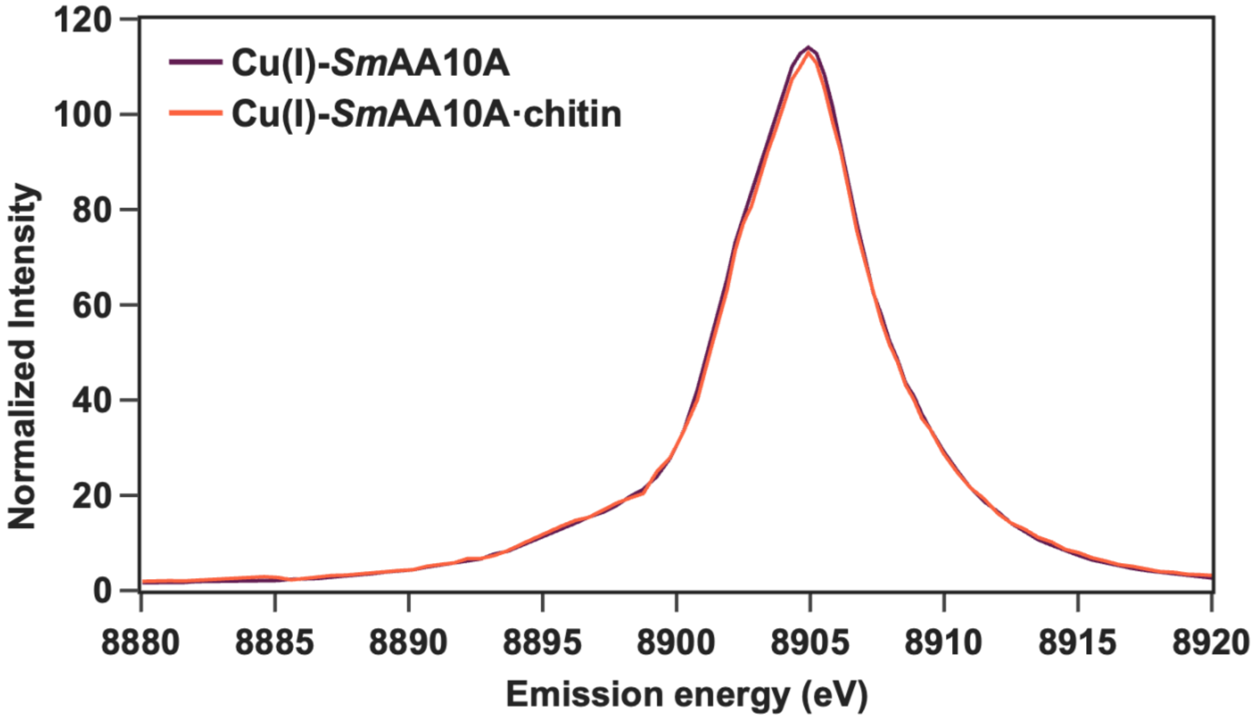

Figure S13. Cu Kβ XES mainlines of **Cu(I)-*Sm*AA10A** (purple) and **Cu(I)-*Sm*AA10A·chitin** (orange). The Kβ XES spectra have been normalized by setting the integral of the entire mainline + valence-to-core region (8880 to 9002 eV) to a value of 1000.

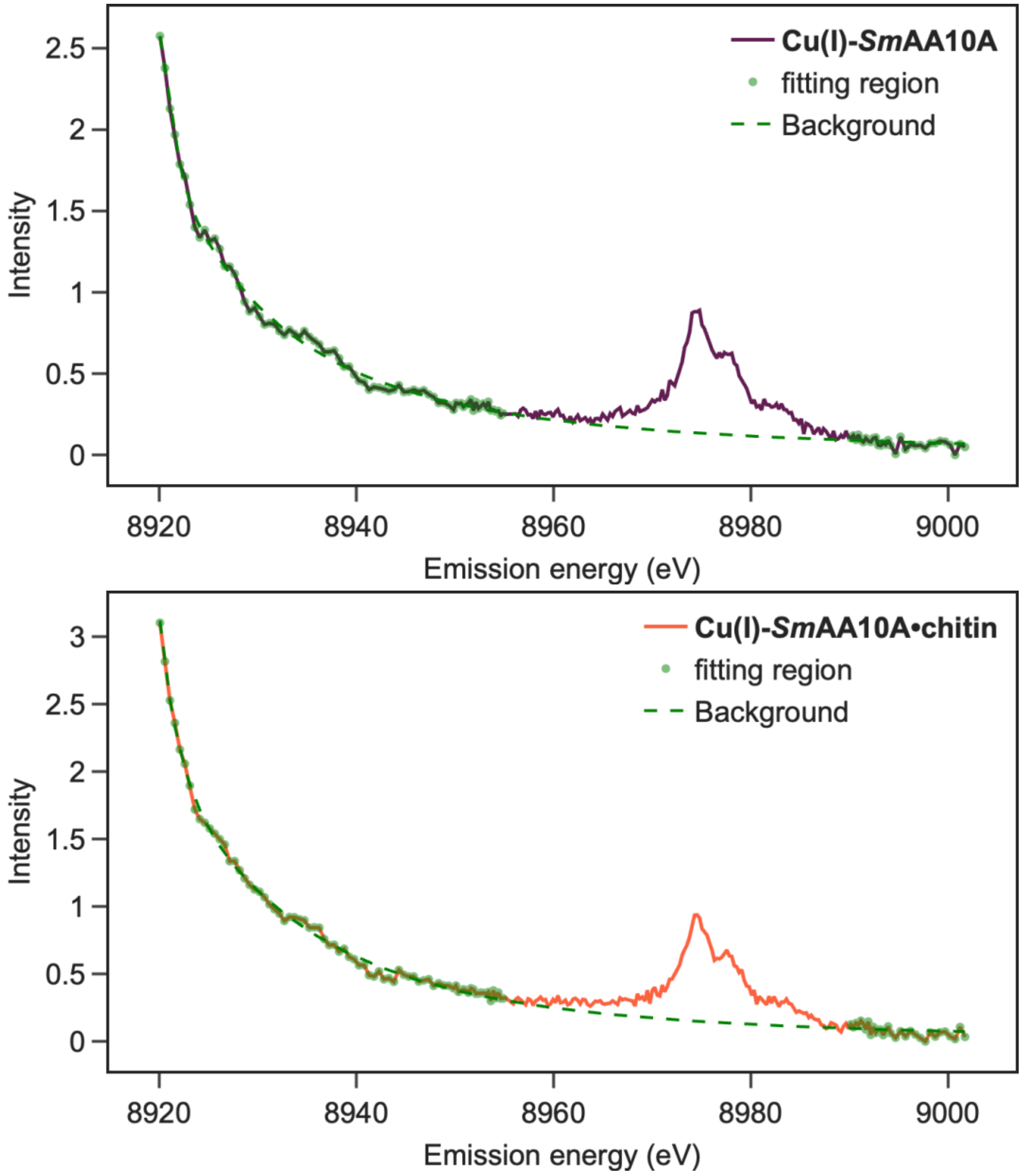

Figure S14. XES data of the VtC region following area normalization. The background of the VtC region could be well-modeled using 2 pseudo-Voigt peaks for data from both **Cu(I)-*Sm*AA10A** (purple, *top*) and **Cu(I)-*Sm*AA10A·chitin** (orange, *bottom*).

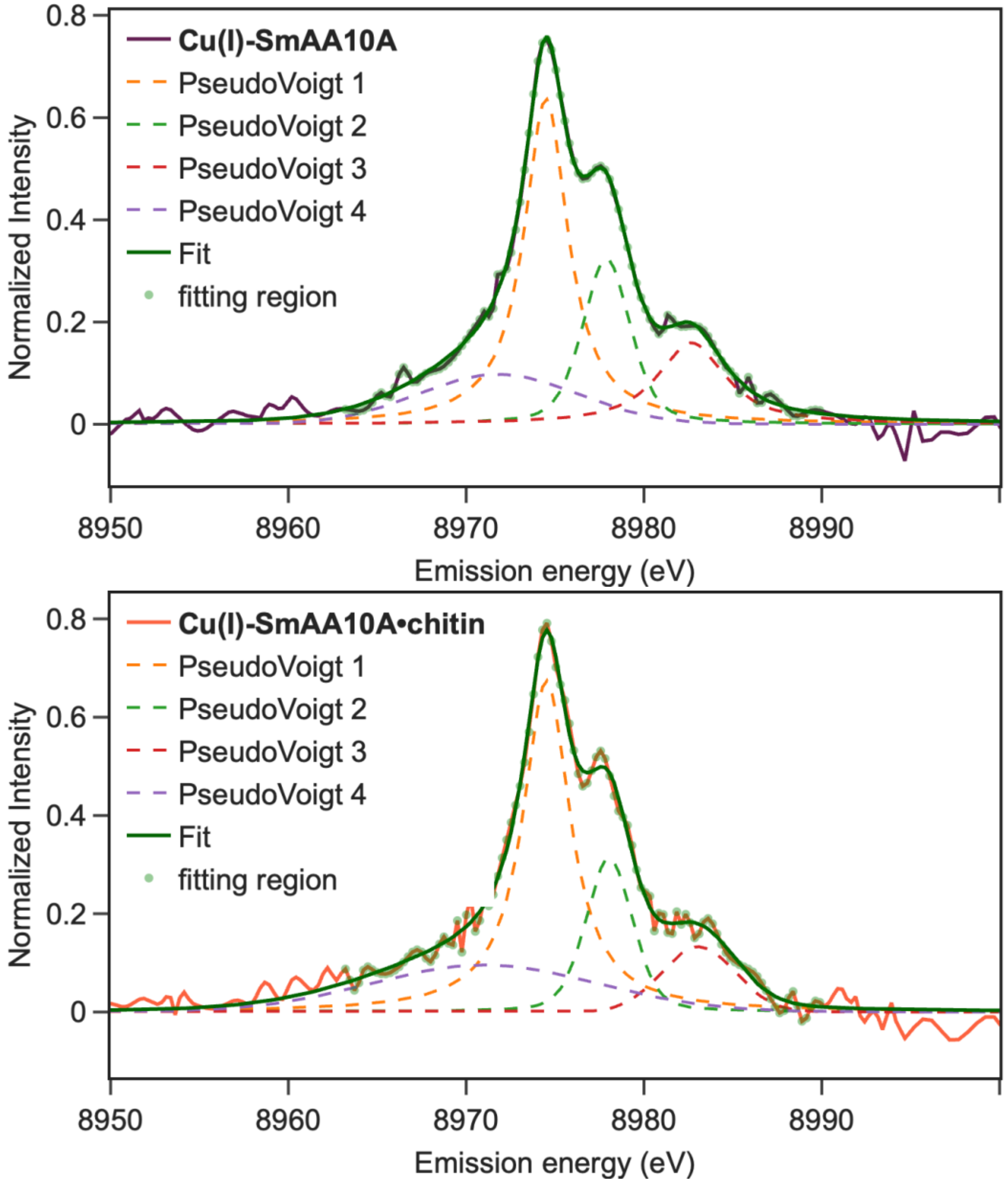

Figure S15. Peak fitting analysis of the VtC-XES spectra for **Cu(I)-*Sm*AA10A** (purple, *top*) and **Cu(I)-*Sm*AA10A·chitin** (orange, *bottom*). A smoothing spline filter (s = 0.05) has been applied to the experimental data prior to fitting to emphasize signal shape.

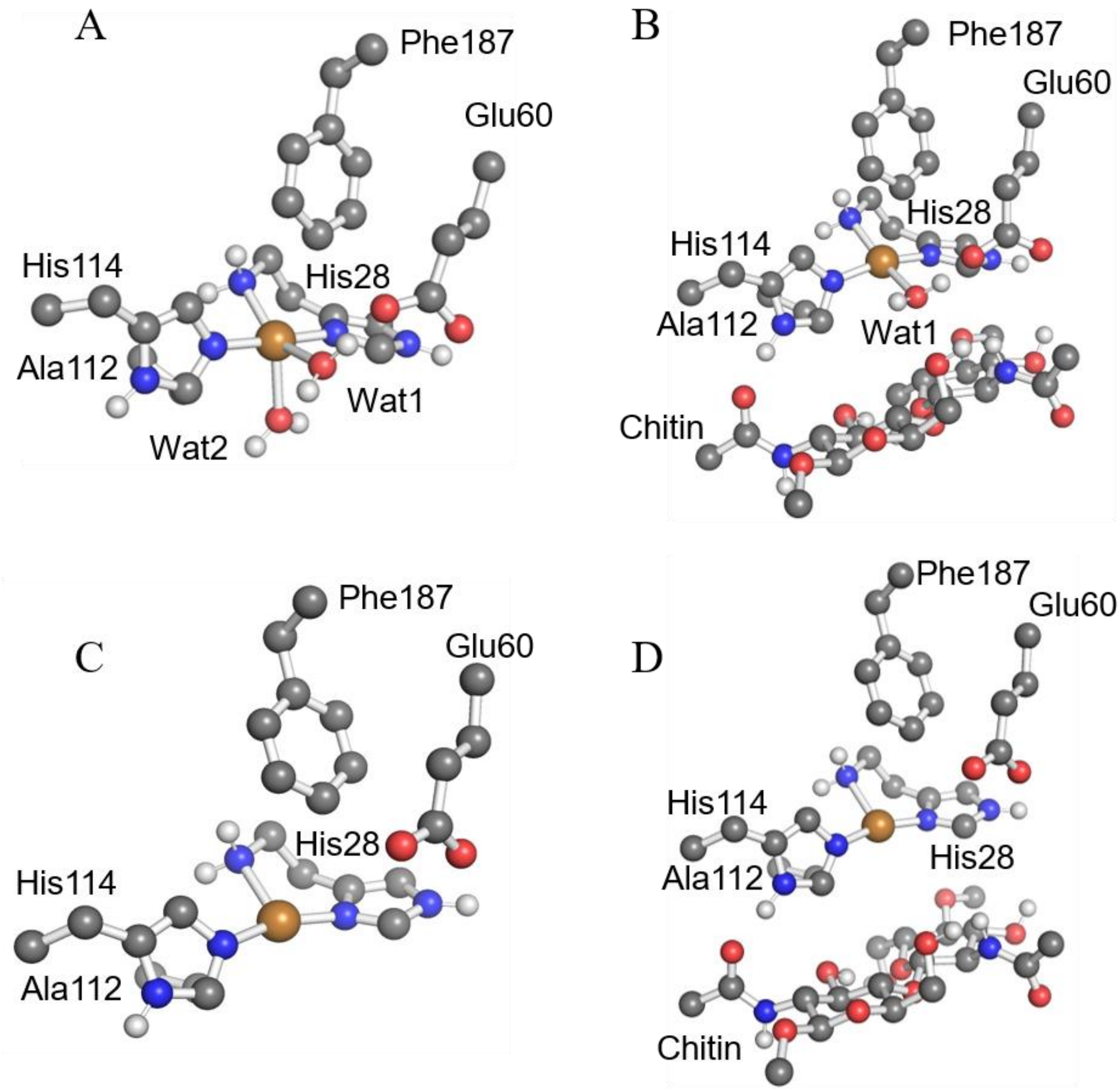

Figure S16. Cluster models for the *Sm*AA10A LPMO. Non-polar hydrogens are omitted for clarity. Illustrated are (a) **Cu(II)-*Sm*AA10A**, (b) **Cu(II)-*Sm*AA10A·chitin**, (c) optimized cluster model for **Cu(I)-*Sm*AA10A**, and (d) optimized cluster model for **Cu(I)-*Sm*AA10A·chitin**. Models (a) and (b) were constructed from optimized QM/MM models in ref. [8]. Model (c) was constructed using coordinates from the published crystal structure of *Sm*AA10A (PDB: 2BEM). Model (d) was constructed by superimposing (c) with (b) and extracting chitin from (b).

Table S11. The optimized parameters for Cu(II) and Cu(I) models.

Model	Cu(II)-*Sm*AA10A[a]	Cu(II)-*Sm*AA10A ·chitin[a]	Cu(I)-*Sm*AA10A	Cu(I)-*Sm*AA10A ·chitin
Cu–$N_{term,His28}$ (Å)	2.11	2.07	2.19	2.21
Cu–$N^{\delta1}_{His28}$ (Å)	1.98	1.94	1.92	1.92
Cu–$N^{\varepsilon2}_{His114}$ (Å)	1.99	1.96	1.92	1.92
Cu–$O_{Wat\ 1}$ (Å)	2.18	2.12	-	-
Cu–$O_{Wat\ 2}$ (Å)	2.26	-	-	-
Cu–H1[b] (Å)	-	3.72	-	3.71
Cu–H4[b] (Å)	-	4.54	-	4.58
Torsion angle[c] (°)	129.6	132.2	140.1	125.4
θ_D (°)	78.6	74.7	58.7	68.9
θ_3 (°)	177.7	166.1	161.5	163.2
θ_T (°)	–0.8	12.3	0.2	0.1

[a] Parameters taken from the optimized QM/MM models adapted from ref. 8

[b] H1 is the hydrogen on the C1 site of chitin. H4 is the hydrogen on the C4 site of chitin.

[c] Defined by $N^{\delta1}_{His28}$–N_{term}–Cu–$N^{\varepsilon2}_{His114}$.

Remarks on DFT-optimized structures. The DFT optimized **Cu(II)-*Sm*AA10A** model (Figure S16) features the expected histidine brace motif with Cu–N_{im} distances of 1.98 Å and 1.99 Å for the imidazole head groups of His28 and His114, respectively, as well as a more elongated Cu–N_{term} distance of 2.11 Å to the N terminus (Table S11). Additionally, two ligated water molecules present Cu–O distances of 2.18 Å and 2.26 Å. We note that the optimized Cu–N_{term} distance is closer to the optimized Cu–O of the proximal water ligand than to the Cu–N_{im} distances, reflecting the slightly ambiguous degeneracy assignment in the EXAFS fitting (Table S4, Fits 4 and 6). As a correlative proxy for the rhombicity enforced by the water ligands upon the Cu site geometry, the angle of each Cu–O bond with respect to the Cu,N,N,N plane (∠O-Cu-CuNNN) was determined, yielding 33.4° and 52.0°, respectively, consistent with the overall rhombic geometry as reflected in the EPR. The chitin-bound **Cu(II)-*Sm*AA10A·chitin** structure exhibits a loss of a water ligand and overall contractions of the Cu–N distances (1.94 Å and 1.96 Å Cu–N_{im} distances for His28 and His114, respectively, and a Cu–N_{term} distance of 2.07 Å). The remaining water ligand, which resides at a distance of 2.12 Å from the Cu, migrates angularly to a ∠O-Cu-CuNNN of 3.71°, reflecting an overall more planar Cu site, consistent with the axial EPR spin Hamiltonian parameters of the species. The Cu–H1 distance (where H1 denotes the hydrogen site on the C1 carbon site of chitin) was found to be 3.72 Å. Considering that H1 is the closest atom to the Cu site from chitin, direct coordination between the Cu site and the chitin substrate is unlikely.

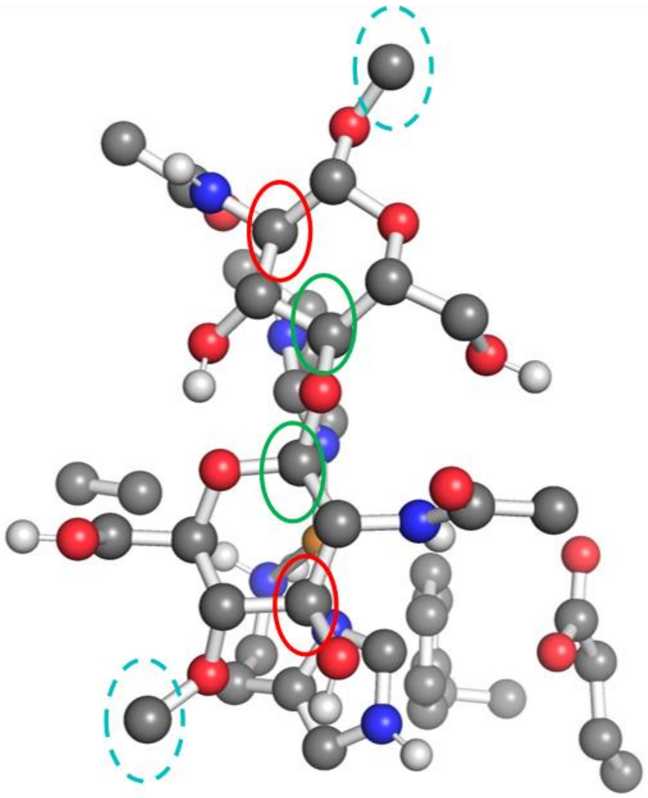

Figure S17. Preliminary cluster model for **Cu(I)-*Sm*AA10A·chitin** optimization setup. Atoms in red circles indicate the *N*-acetylglucosamine (NAG) atoms used to constrain individual NAG monomeric units to other protein residues in Orca (Fragment Constraints protocol), atoms in dashed blue circles indicate fixed carbon atoms. Atoms in green circles indicate the atoms used to connect the two NAG units (green).

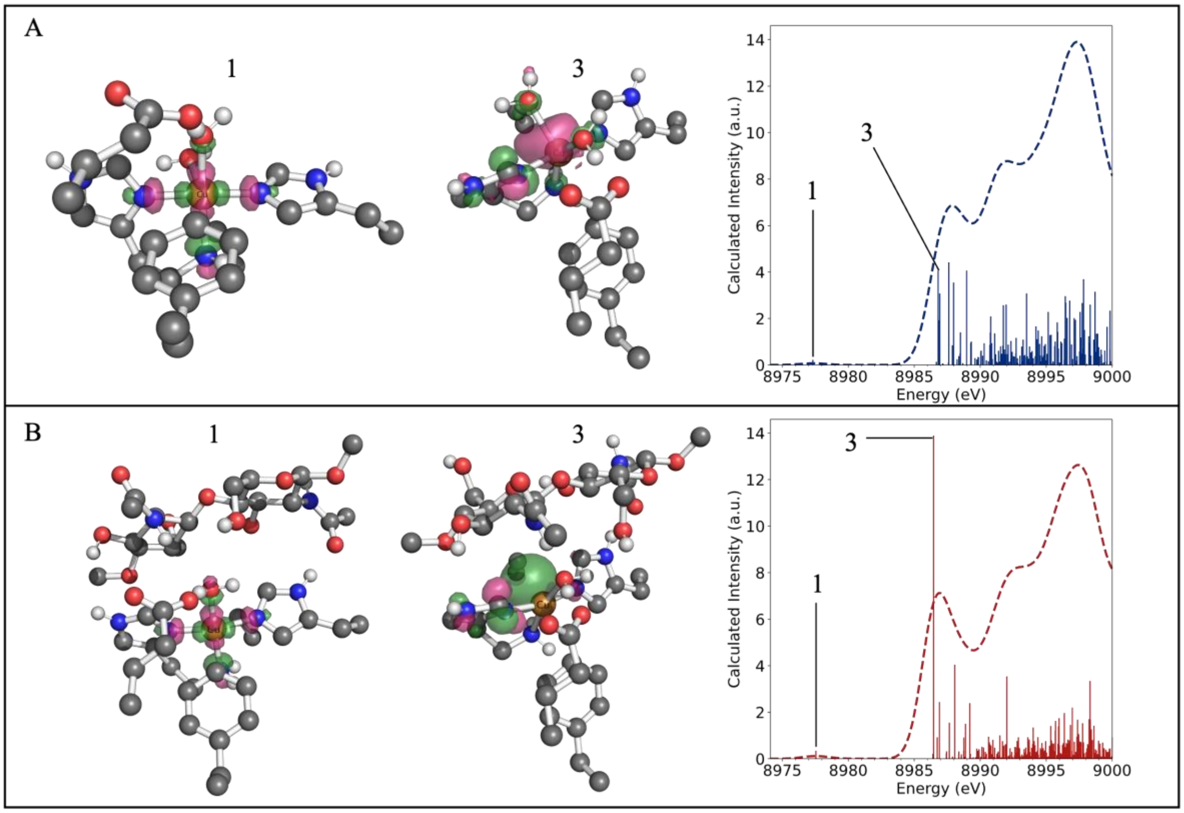

Figure S18. Calculated XAS spectra for the Cu(II) structures, visualized alongside selected natural transition orbitals (NTOs), for (a) **Cu(II)-*Sm*AA10A** and (b) **Cu(II)-*Sm*AA10A·chitin**. Note that the transition intensities (sticks) are scaled up by a factor of 3.

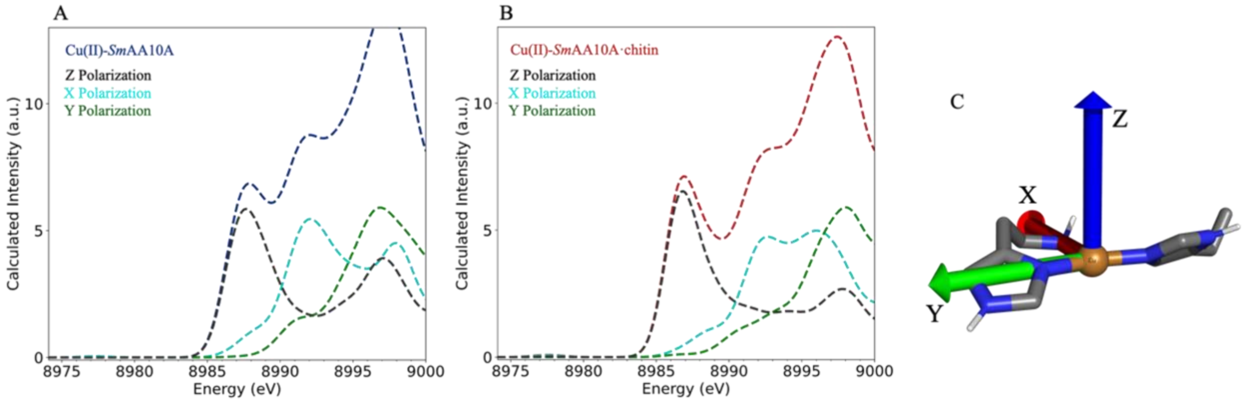

Figure S19. Calculated Cu K-edge XAS spectra with *xyz* polarization components for the models (a) **Cu(II)-*Sm*AA10A** and (b) **Cu(II)-*Sm*AA10A·chitin**. The cartesian orientation of the models is depicted in (c) on the right.

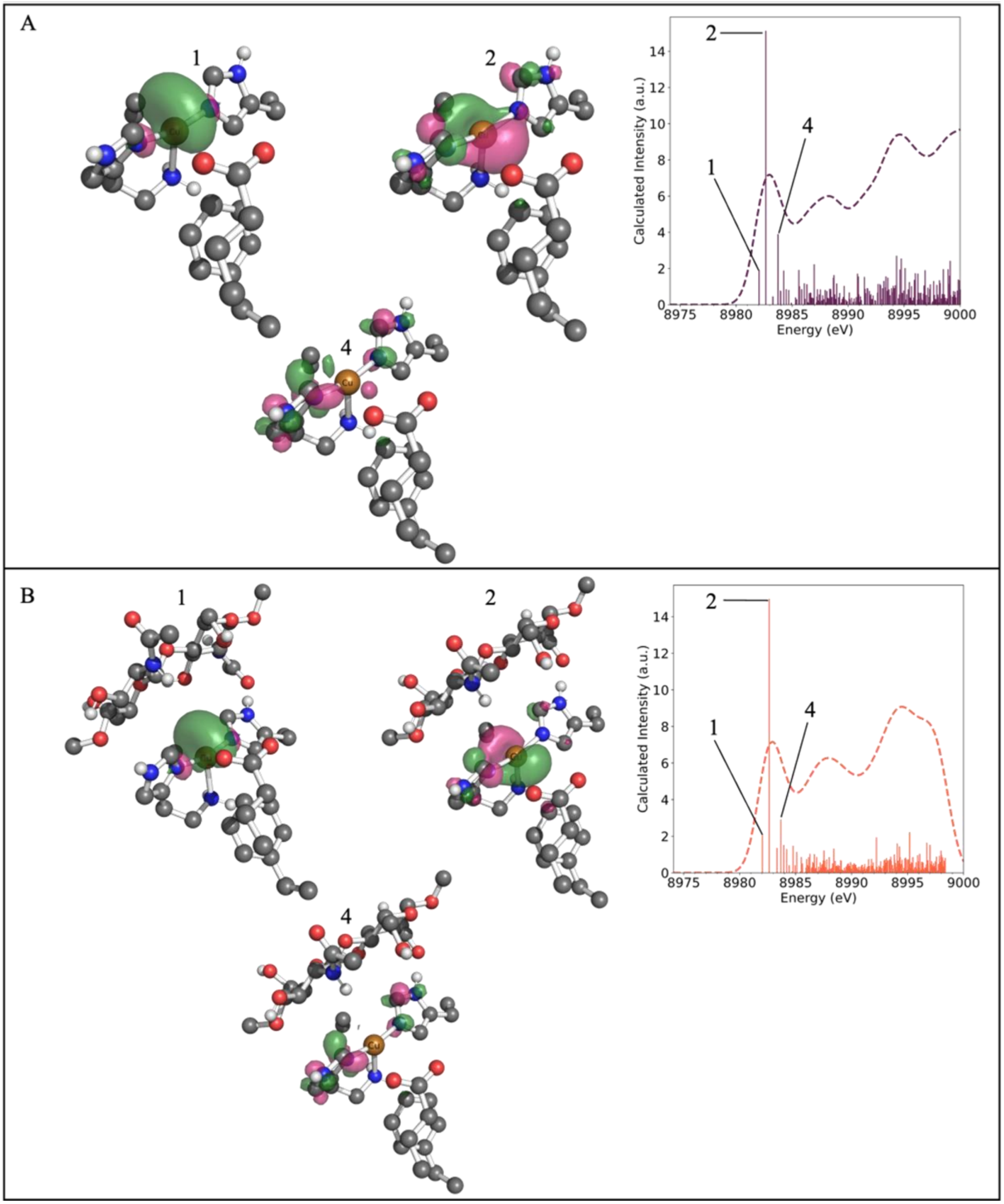

Figure S20. Calculated XAS spectra for the Cu(I) structures visualized alongside selected natural transition orbitals (NTOs) for (a) **Cu(I)-*Sm*AA10A** and (b) **Cu(I)-*Sm*AA10A·chitin**. Note that the transition intensities (sticks) are scaled up by a factor of 3.

Remarks on calculated natural transition orbitals. NTO analysis of the feature reveals contributions from three main transitions (Figure S20). First, a less intense feature at lower energies (8982.0 eV for both **Cu(I)-*Sm*AA10A** and **Cu(I)-*Sm*AA10A·chitin**) in both spectra exhibits Cu 4*s* character (Figure S20, NTO #1). This is followed by an intense, higher-energy feature at ~8982.7 eV in both spectra (Figure S20, NTO #2) which predominantly displays Cu $4p_z$ character, with some charge-transfer character into the imidazole rings π^*. The total Cu contribution versus imidazole π* of His28 is similar: 28/26% for chitin-free and 27/31% for chitin-bound systems. Lastly, the transition at 8983.7 eV (Figure S20, NTO #4) in both **Cu(I)-*Sm*AA10A** and **Cu(I)-*Sm*AA10A·chitin** is predominantly composed of Cu $4p_z$ character admixed with charge transfer to the histidine brace imidazole ring π* acceptor orbital (14/41% in **Cu(I)-*Sm*AA10A** and 10/38% in **Cu(I)-*Sm*AA10A·chitin)**.

Calculated Valence-to-Core Spectra.

The calculated VtC spectra (Figure S21) reproduce the overall profiles observed in the experimental spectra, though a clear increase in intensity at the lower-energy feature for **Cu(I)-*Sm*AA10A·chitin** is apparent in the calculated data. The calculated Kβ'' region shows two low-intensity features at 8960.5 eV and 8967.3 eV associated with ligand 2*s* to Cu 1*s* transitions, which are not observed experimentally. The K$\beta_{2,5}$ region at 8970–8983 eV is characterized by two peaks. Examination of the molecular orbitals for both **Cu(I)-*Sm*AA10A** and **Cu(I)-*Sm*AA10A·chitin** (Figure S22) reveals transitions similar to those in the reported spectrum for solution-state *Hj*LPMO9A.[38] The more intense feature in the K$\beta_{2,5}$ region at 8974.5 eV is dominated by a transition originating from Cu–N_{im} σ orbital. This orbital exhibits 3.4% and 3.5% Cu p_y character and 2.6% and 2.3% Cu d_{xy} character for the chitin-free and chitin-bound structures, respectively. A less intense feature at 8978.7 eV is primarily attributed to a transition originating from an orbital predominantly composed of Cu 3$d_{x^2-y^2}$ (65.3–61.0%) admixed with 5.9–5.5% Cu p_x. Transition polarization component analyses for both structures (Figure S23) reveal the Kβ'' to be almost entirely dominated by *y*-polarized transitions. In the K$\beta_{2,5}$ region, the lower-energy 8974.5 eV feature is predominantly *y*-polarized with minor additional contribution from the *x* direction, while the higher-energy 8978.7 eV feature is mostly *x*-polarized with significant contribution from the *y* direction, consistent with the molecular orbitals analysis.

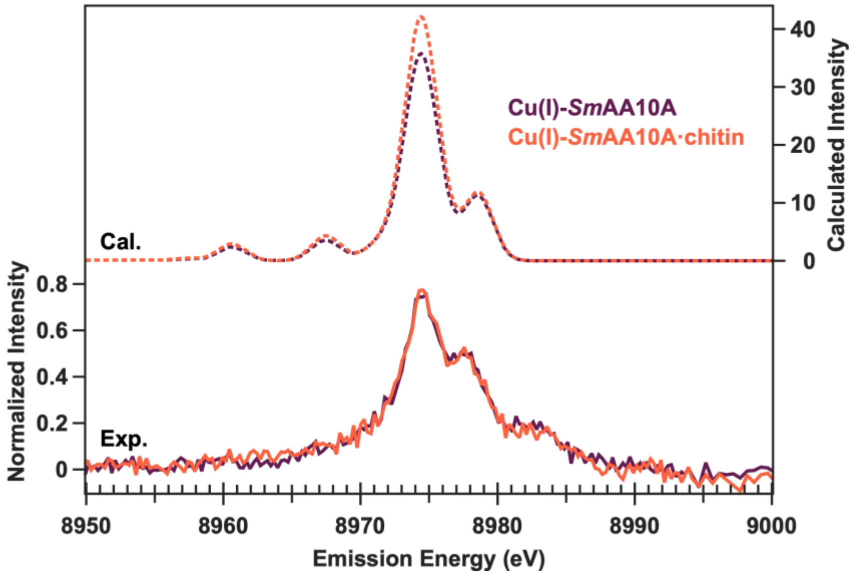

Figure S21. Experimental (solid lines) and calculated (dashed lines) VtC-XES spectra for **Cu(I)-*Sm*AA10A** (purple) and **Cu(I)-*Sm*AA10A·chitin** (orange) samples.

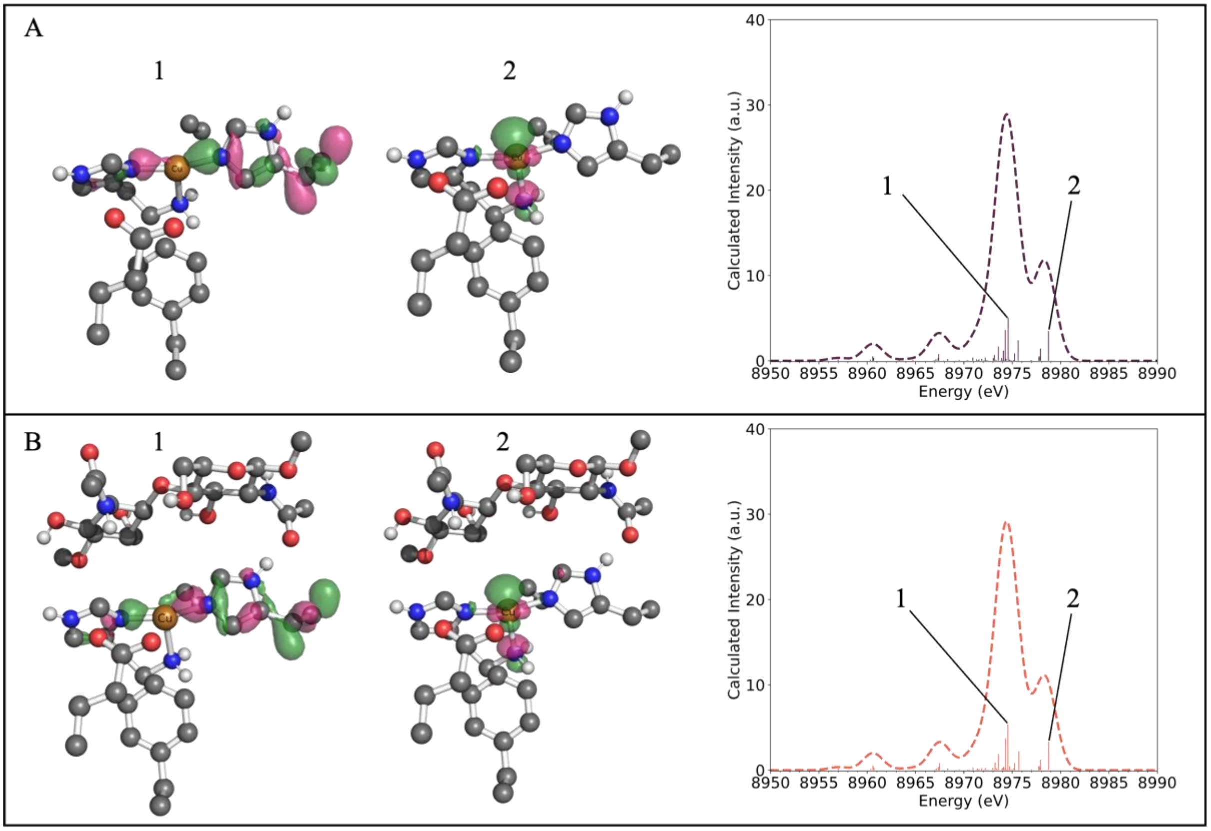

Figure S22. Calculated VtC-XES spectra for Cu(I) structures visualized alongside selected molecular orbitals for (a) **Cu(I)-*Sm*AA10A** and (b) **Cu(I)-*Sm*AA10A·chitin**.

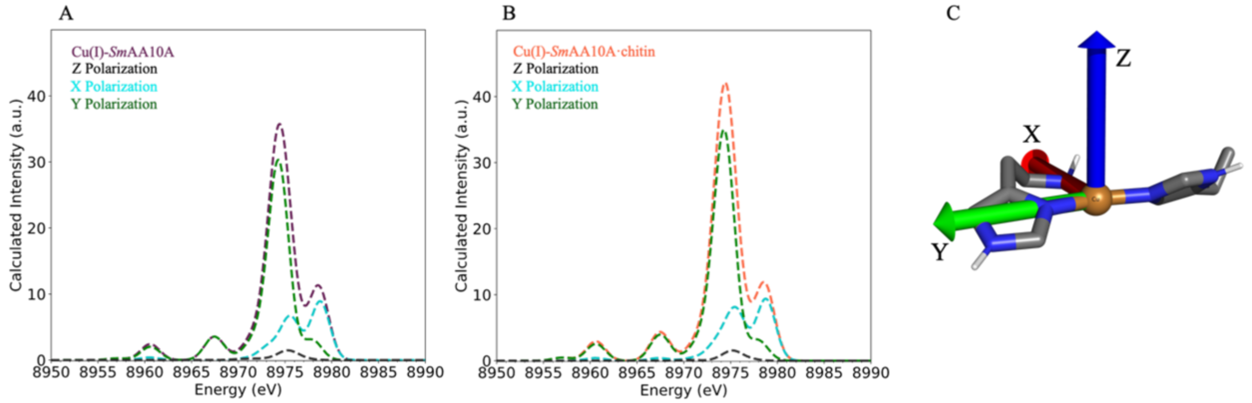

Figure S23. Calculated VtC-XES spectra with *xyz* polarization components for the models (a) **Cu(I)-*Sm*AA10A** and (b) **Cu(I)-*Sm*AA10A·chitin**. The cartesian orientation of the models is depicted in (c) on the right.

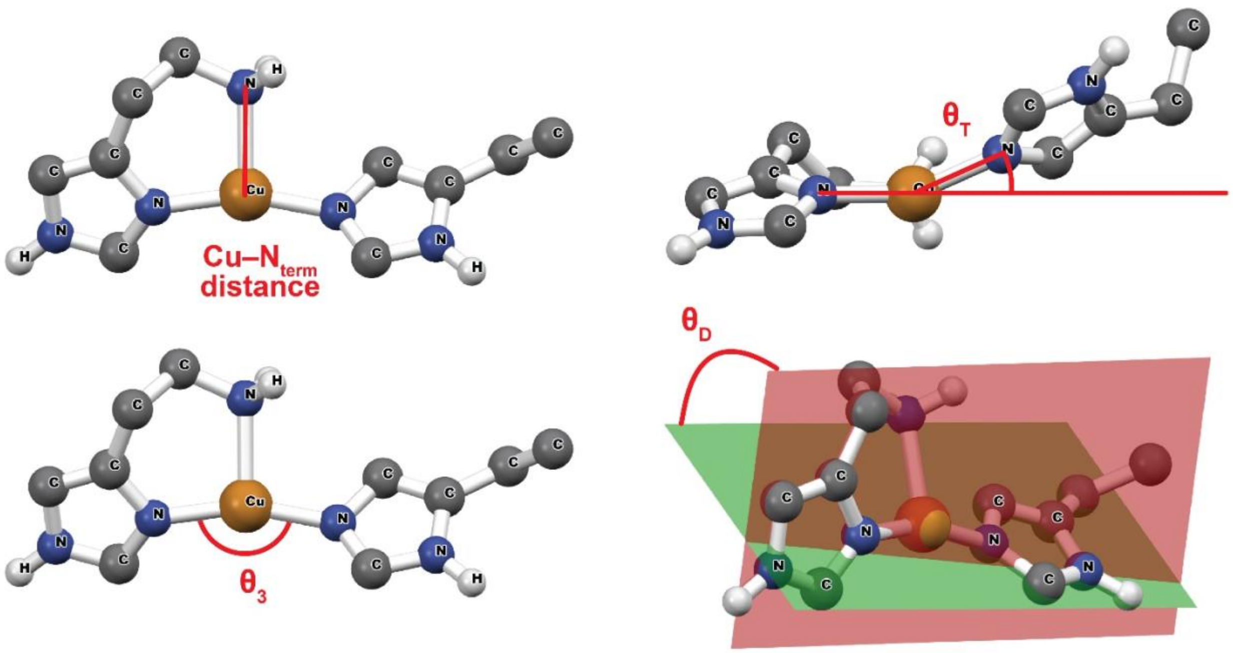

Figure S24. Histidine brace toy models with indications of the conformational parameters investigated: (1) Cu–N$_{term}$ distance, (2) angle θ_T between the Cu–N$^{\varepsilon 2}_{His114}$ axis and the Cu,N$^{\delta 1}_{His28}$,N$_{term}$ His brace plane, (3) angle θ_3 defined by N$^{\varepsilon 2}_{His114}$–Cu–N$^{\delta 1}_{His28}$ and controlled by the rotation angle of the His114 head group around the *z* axis (see Figure S23c for orientation) to emulate T-to-Y geometric deformation, and (4) angle θ_D defined as the dihedral angle between the two imidazole rings. The plane for each imidazole ring was determined by calculating the least-squared plane for the atomic coordinates of the non-hydrogen atoms. Structures for θ_D were generated by torsional rotation of the His114 head group around the Cu–N$^{\varepsilon 2}_{His114}$ bond.

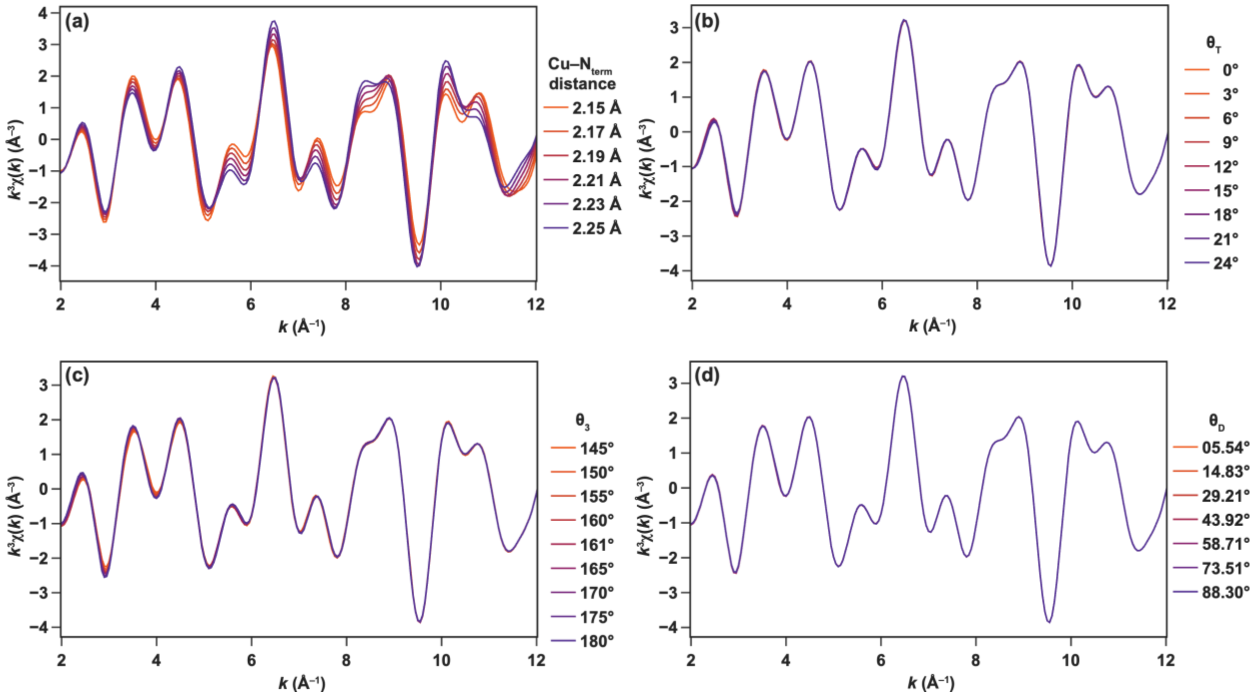

Figure S25. *FEFF10*-calculated EXAFS on Histidine brace toy complexes iterated through the four conformational parameters (a) Cu–N_{term}, (b) θ_T, (c) θ_3, and (d) θ_D.

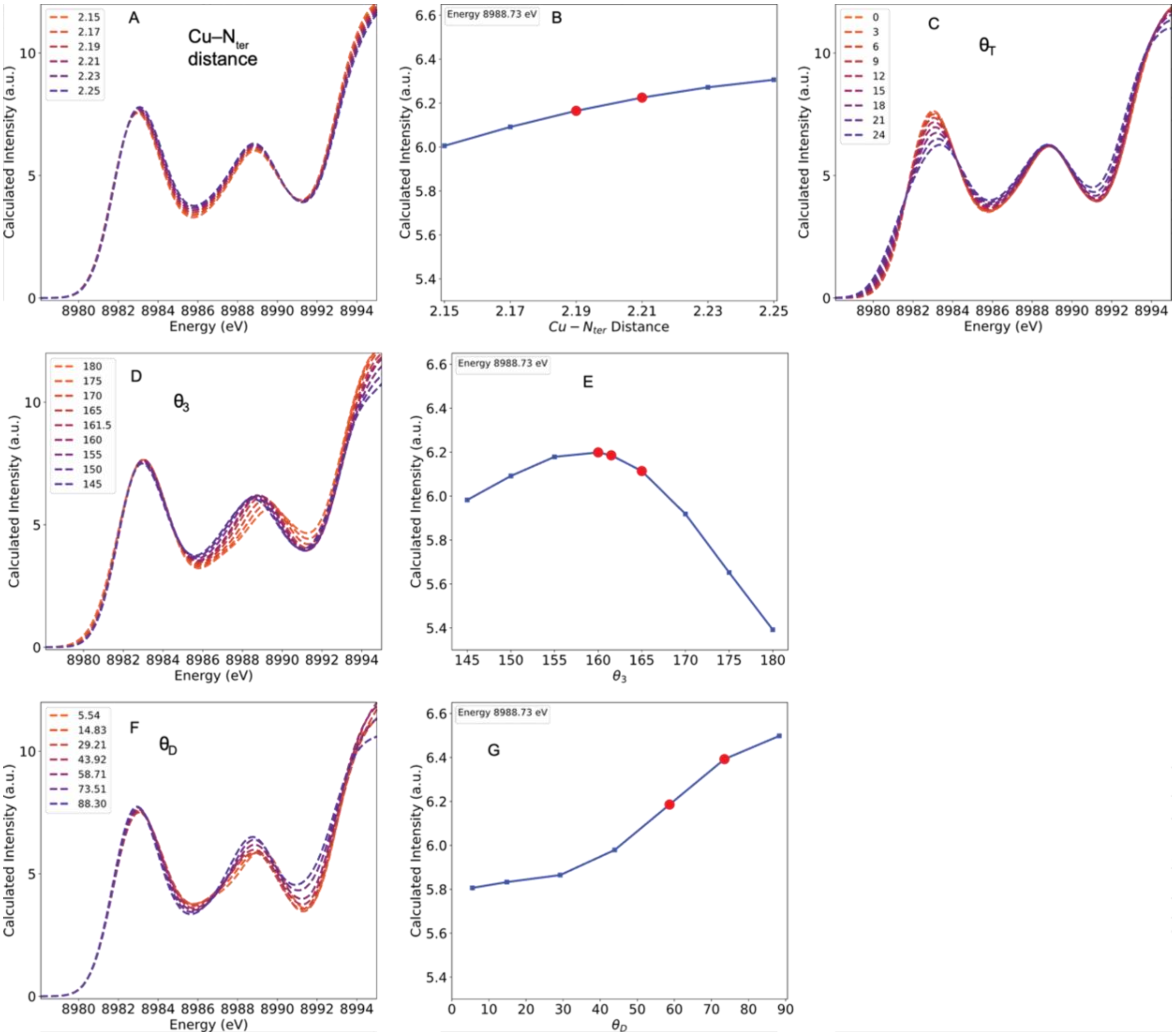

Figure S26. TDDFT-calculated Cu K-edge XAS spectra on Histidine brace toy complexes iterated through the four conformational parameters: (a, b) Cu–N_{term}, (c) θ_T, (d,e) θ_3, and (f,g) θ_D. Figures (b), (e), and (g) depict intensity profiles at 8988.73 eV, with the red points indicating values near those found in the *Sm*AA10A cluster models.

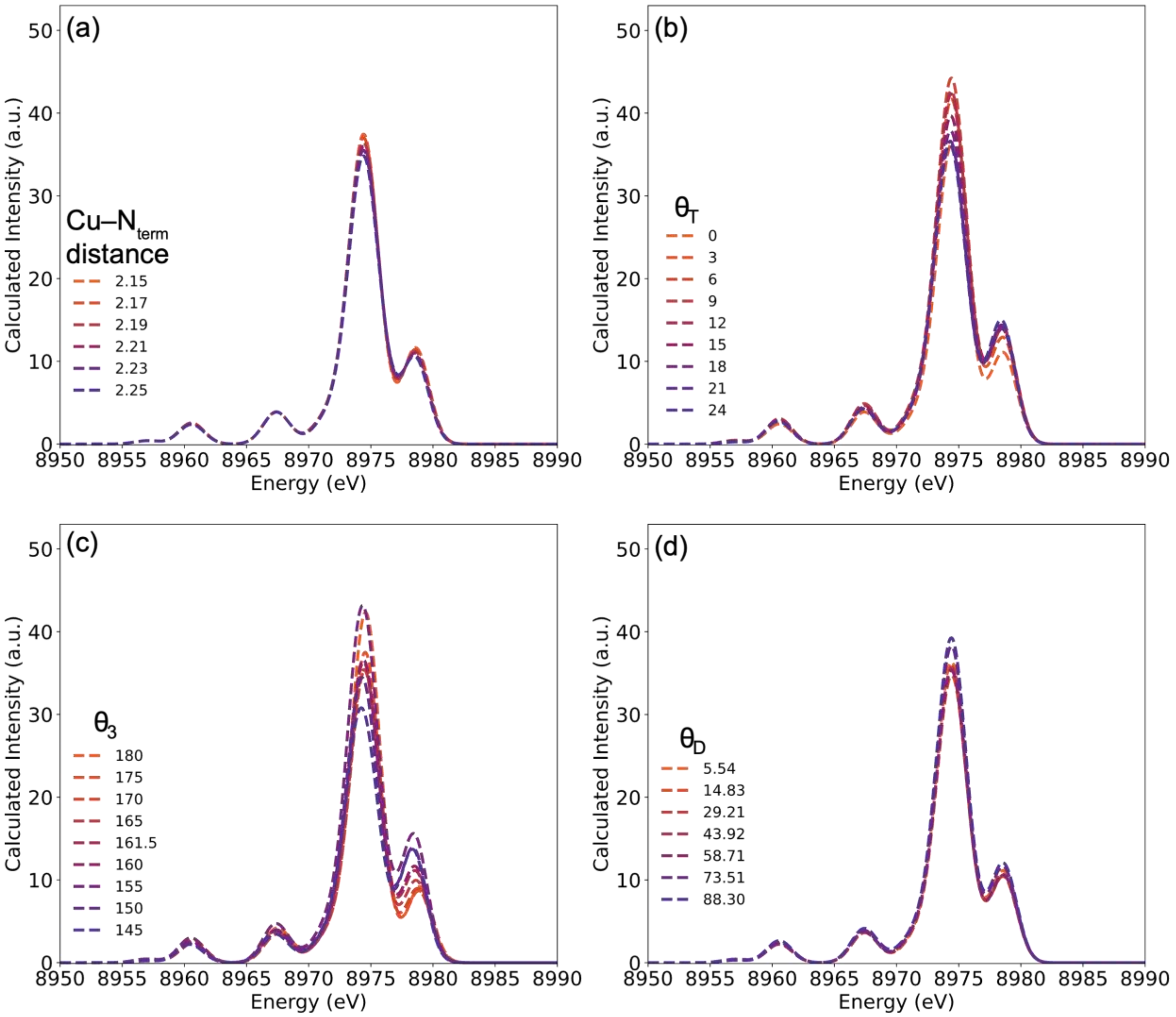

Figure S27. DFT-calculated VtC-XES on Histidine brace toy complexes iterated through the four conformational parameters (a) Cu–N_{term}, (b) θ_T, (c) θ_3, and (d) θ_D.

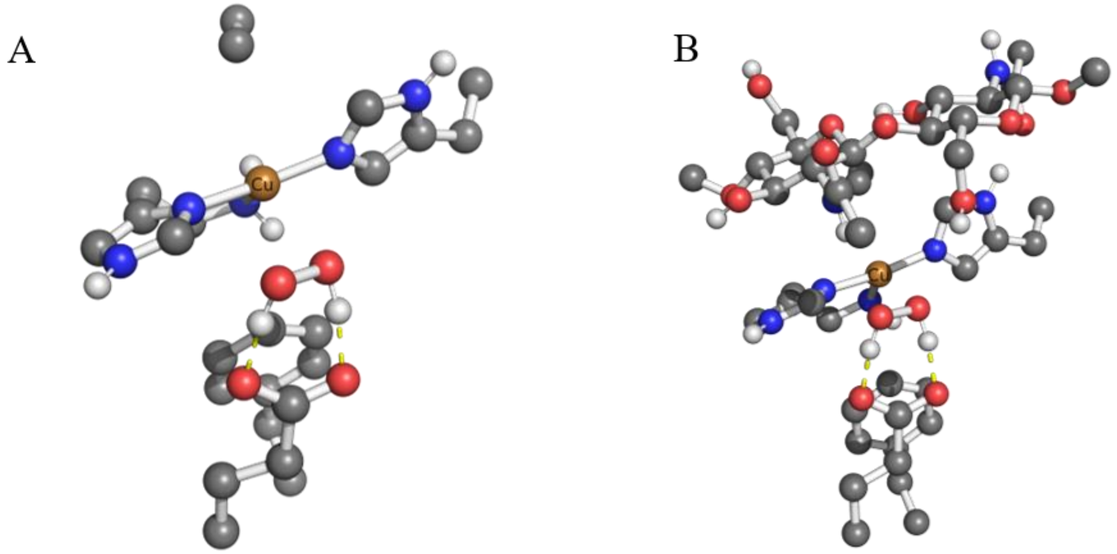

Figure S28. Optimized geometries at BP86/def2-TZVP level of (A) **Cu(I)-*Sm*AA10A·H$_2$O$_2$** and (B) **Cu(I)-*Sm*AA10A·chitin·H$_2$O$_2$** with H$_2$O$_2$ bound to Glu60, used for binding energy calculations.

Table S12. Binding energy calculations for H$_2$O$_2$. The values apply to calculations for the cluster models of **Cu(I)-*Sm*AA10A·H$_2$O$_2$** and **Cu(I)-*Sm*AA10A·chitin·H$_2$O$_2$** at the BP86/-def2-TZVP level of theory.

**Model**	**Electronic energy, E (E_h)**	**Enthalpy, H (E_h)**	**Gibbs free energy, G (E_h)**	**Entropy, S (E_h)**
H_2O_2	-151.641	-151.612	-151.636	0.024
**Cu(I)-*Sm*AA10A**	-3004.411	-3004.043	-3004.399	0.357
**Cu(I)-*Sm*AA10A·H$_2$O$_2$**	-3156.084	-3155.685	-3156.047	0.362
**Cu(I)-*Sm*AA10A·chitin**	-4647.409	-4646.874	-4647.763	0.888
**Cu(I)-*Sm*AA10A·chitin·H$_2$O$_2$**	-4799.084	-4798.519	-4799.413	0.894
**Δ (Difference Between Models)**	**ΔE (kcal/mol)**	**ΔH (kcal/mol)**	**ΔG (kcal/mol)**	**-TΔS (kcal/mol)**
**Cu(I)-*Sm*AA10A·H$_2$O$_2$ – Cu(I)-*Sm*AA10A**	-19.715	-18.983	-7.525	11.458
**Cu(I)-*Sm*AA10A·chitin·H$_2$O$_2$ – Cu(I)-*Sm*AA10A·chitin**	-21.546	-20.951	-9.548	11.404
**ΔΔ (Relative Difference)**	**ΔΔE (kcal/mol)**	**ΔΔH (kcal/mol)**	**ΔΔG (kcal/mol)**	**-TΔΔS (kcal/mol)**
	-1.831	-1.968	-2.023	-0.055

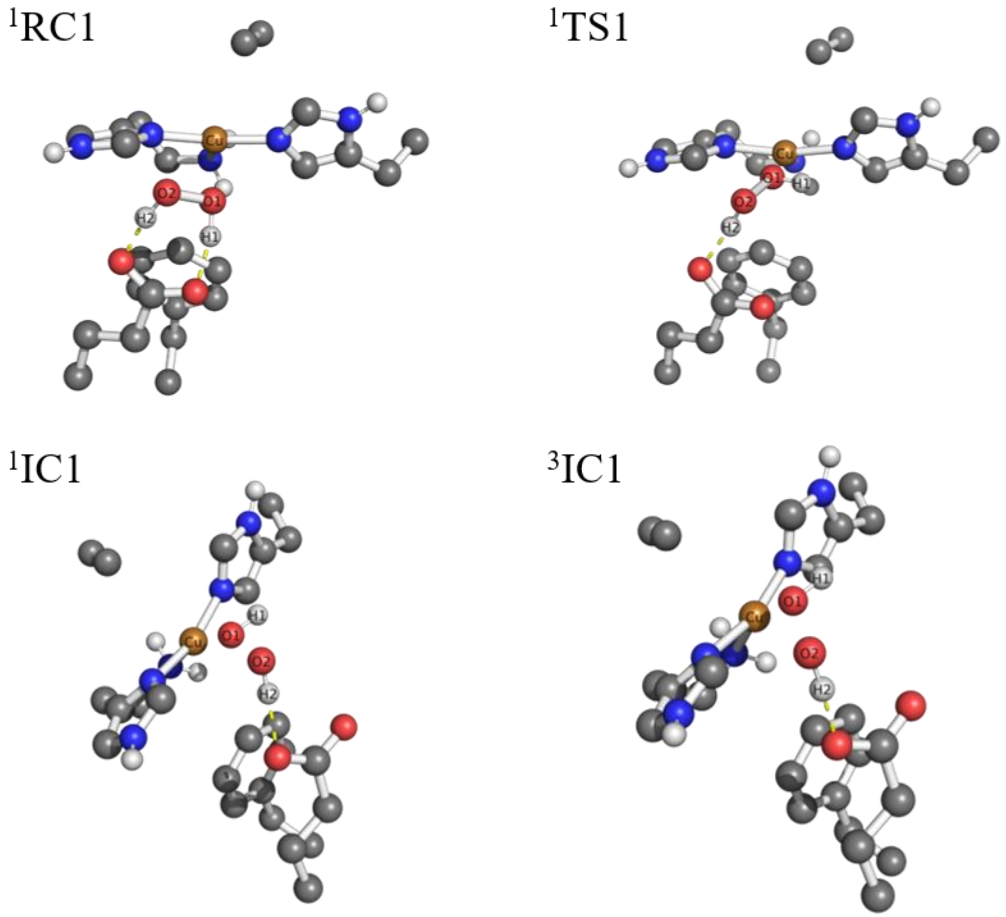

Figure S29. Optimized geometries by DFT for **Cu(I)-*Sm*AA10A·H_2O_2**. Optimized geometries are shown for the reactant complex (RC), the transition state of the O–O homolytic cleavage (TS) and the intermediate complexes of the Cu(II)OH + OH• step (IC1) found along the H_2O_2 splitting path of the reaction. The left superscript indicates the spin multiplicity.

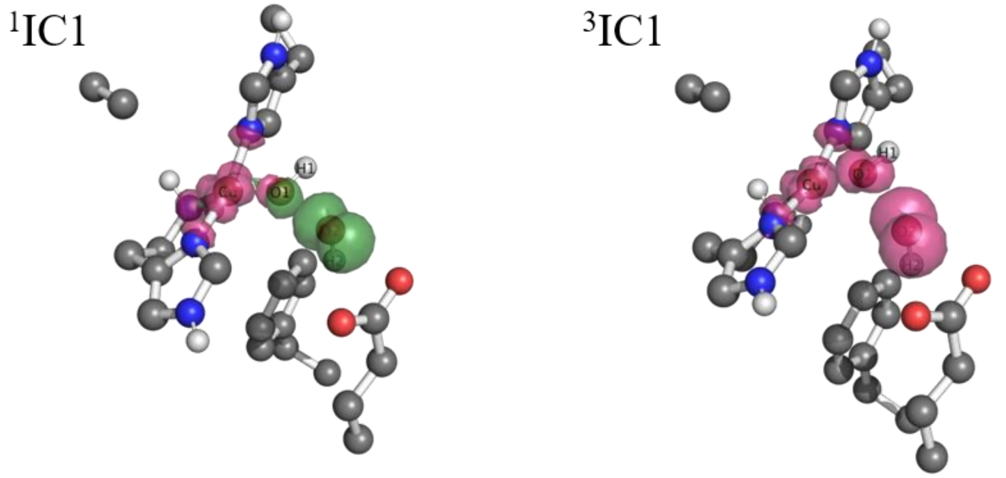

Figure S30. Spin density plots for intermediate complexes for the reaction with **Cu(I)-*Sm*AA10A·H_2O_2**. Net positive spin is indicated by magenta and net negative spin by forest green. Spin population values are listed in Table S13.

Table S13. Key parameters of optimized structures along the H_2O_2 activation pathway. The values apply to calculations for the cluster models of **Cu(I)-*Sm*AA10A·H_2O_2** and **Cu(I)-*Sm*AA10A·chitin·H_2O_2** at the B3LYP/ZORA-def2-TZVP level of theory.

Parameter	Model	^{1}RC	^{1}TS	^{1}IC1	^{3}IC1
O1–O2 (Å)[a]	Cu(I)-*Sm*AA10A·H_2O_2	1.462	1.718	2.292	3.366
	Cu(I)-*Sm*AA10A·chitin·H_2O_2	1.462	1.735	2.290	2.330
Cu–O1 (Å)	Cu(I)-*Sm*AA10A·H_2O_2	3.790	2.142	1.919	1.914
	Cu(I)-*Sm*AA10A·chitin·H_2O_2	3.756	2.205	1.951	1.950
H1–O1 (Å)	Cu(I)-*Sm*AA10A·H_2O_2	1.006	0.969	0.964	0.963
	Cu(I)-*Sm*AA10A·chitin·H_2O_2	1.008	0.996	0.980	0.983
H2–O2 (Å)	Cu(I)-*Sm*AA10A·H_2O_2	1.008	0.995	0.993	1.031
	Cu(I)-*Sm*AA10A·chitin·H_2O_2	1.010	0.992	0.987	0.985
Cu–N(His28)	Cu(I)-*Sm*AA10A·H_2O_2	1.927	1.953	1.955	1.959
	Cu(I)-*Sm*AA10A·chitin·H_2O_2	1.921	1.945	1.938	1.936
Cu–N(His114)	Cu(I)-*Sm*AA10A·H_2O_2	1.927	1.934	1.947	1.958
	Cu(I)-*Sm*AA10A·chitin·H_2O_2	1.922	1.935	1.958	1.977
Cu–$N_{\mathbf{term}}$(His28)	Cu(I)-*Sm*AA10A·H_2O_2	2.259	2.283	2.195	2.210
	Cu(I)-*Sm*AA10A·chitin·H_2O_2	2.272	2.289	2.142	2.137
Cu–Glu60(O_{E1}) [b]	Cu(I)-*Sm*AA10A·H_2O_2	5.237	4.867	4.902	5.154
	Cu(I)-*Sm*AA10A·chitin·H_2O_2	5.069	4.753	4.692	4.685
Cu–Glu60(O_{E2}) [b]	Cu(I)-*Sm*AA10A·H_2O_2	5.268	5.334	5.297	5.239
	Cu(I)-*Sm*AA10A·chitin·H_2O_2	4.987	3.975	4.035	4.048
Cu-Phe187(Cz)	Cu(I)-*Sm*AA10A·H_2O_2	3.576	3.454	3.425	3.467
	Cu(I)-*Sm*AA10A·chitin·H_2O_2	3.503	3.864	3.667	3.655
Cu-O1-O2 (°)	Cu(I)-*Sm*AA10A·H_2O_2	112.8	160.4	153.0	137.9
	Cu(I)-*Sm*AA10A·chitin·H_2O_2	113.1	152.3	140.2	130.9
**H1-O1-O2-H2 dihedral (°)**	Cu(I)-*Sm*AA10A·H_2O_2	33.35	113.58	108.32	102.93
	Cu(I)-*Sm*AA10A·chitin·H_2O_2	29.60	37.05	26.29	18.62
Cu spin pop.[c]	Cu(I)-*Sm*AA10A·H_2O_2	0.000	0.231	0.624	0.649
	Cu(I)-*Sm*AA10A·chitin·H_2O_2	0.000	-0.252	0.639	0.690
O1 spin pop. [c]	Cu(I)-*Sm*AA10A·H_2O_2	0.000	0.000	-0.035	0.161
	Cu(I)-*Sm*AA10A·chitin·H_2O_2	0.000	0.020	-0.159	0.409
O2 spin pop. [c]	Cu(I)-*Sm*AA10A·H_2O_2	0.000	-0.277	-0.757	0.947
	Cu(I)-*Sm*AA10A·chitin·H_2O_2	0.000	0.277	-0.661	0.646
N_{term} (His28) spin pop.[c]	Cu(I)-*Sm*AA10A·H_2O_2	0.000	0.016	0.058	0.055
	Cu(I)-*Sm*AA10A·chitin·H_2O_2	0.000	-0.018	0.067	0.069
N (His28) spin pop.[c]	Cu(I)-*Sm*AA10A·H_2O_2	0.000	0.016	0.048	0.050
	Cu(I)-*Sm*AA10A·chitin·H_2O_2	0.000	-0.019	0.056	0.056
N (His114) spin pop.[c]	Cu(I)-*Sm*AA10A·H_2O_2	0.000	0.017	0.046	0.053
	Cu(I)-*Sm*AA10A·chitin·H_2O_2	0.000	-0.018	0.044	0.056
Cu charge pop. [c]	Cu(I)-*Sm*AA10A·H_2O_2	-0.005	0.109	0.262	0.246
	Cu(I)-*Sm*AA10A·chitin·H_2O_2	-0.004	0.123	0.284	0.283
O1 charge pop. [c]	Cu(I)-*Sm*AA10A·H_2O_2	-0.249	-0.176	-0.344	-0.441
	Cu(I)-*Sm*AA10A·chitin·H_2O_2	-0.240	-0.212	-0.362	-0.360
O2 charge pop. [c]	Cu(I)-*Sm*AA10A·H_2O_2	-0.247	-0.323	-0.359	-0.288
	Cu(I)-*Sm*AA10A·chitin·H_2O_2	-0.202	-0.282	-0.316	-0.317

[a] Involving the H_2O_2 oxygen atoms, in which O1 is the atom closer to the copper atom.

[b] O_{E1}, O_{E2} are referred to as the two oxygen atoms of the Glu60 residue.

[c] Hirshfeld populations.

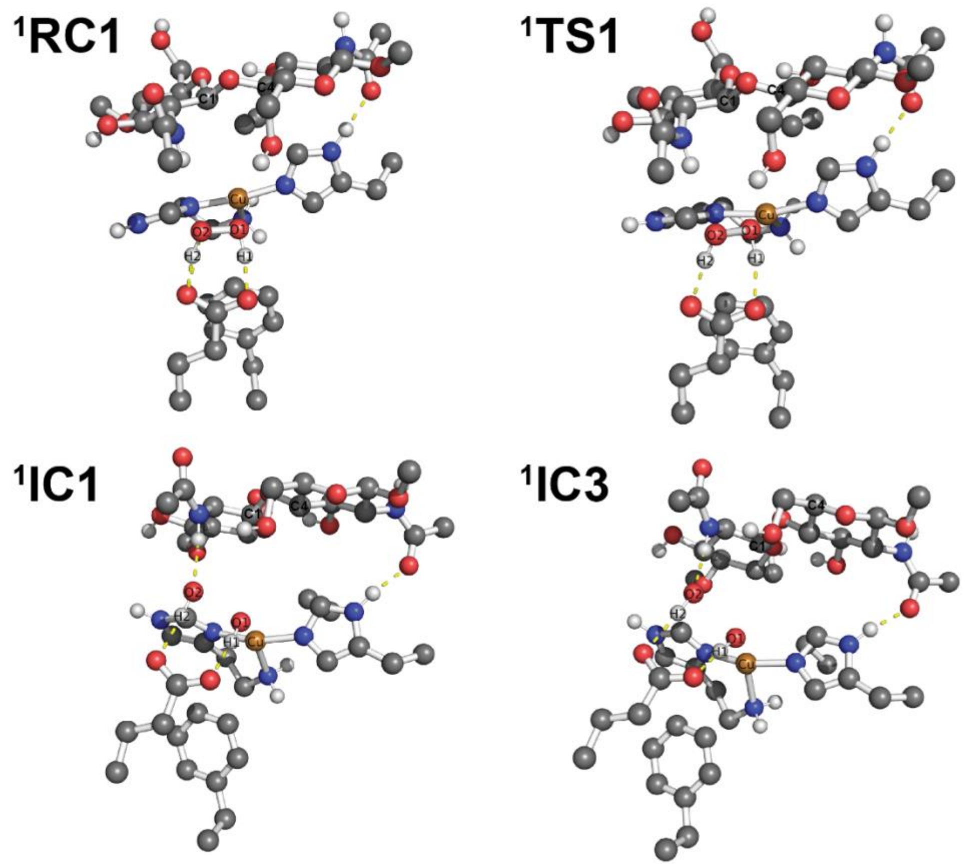

Figure S31. Optimized geometries by DFT for **Cu(I)-*Sm*AA10A·chitin·H_2O_2**. Optimized geometries are shown for the reactant complex (RC), the transition state of the O–O homolytic cleavage (TS) and the intermediate complexes of the Cu(II)OH + OH• step (IC1) found along the H_2O_2 splitting path. The left superscript indicates the spin multiplicity.

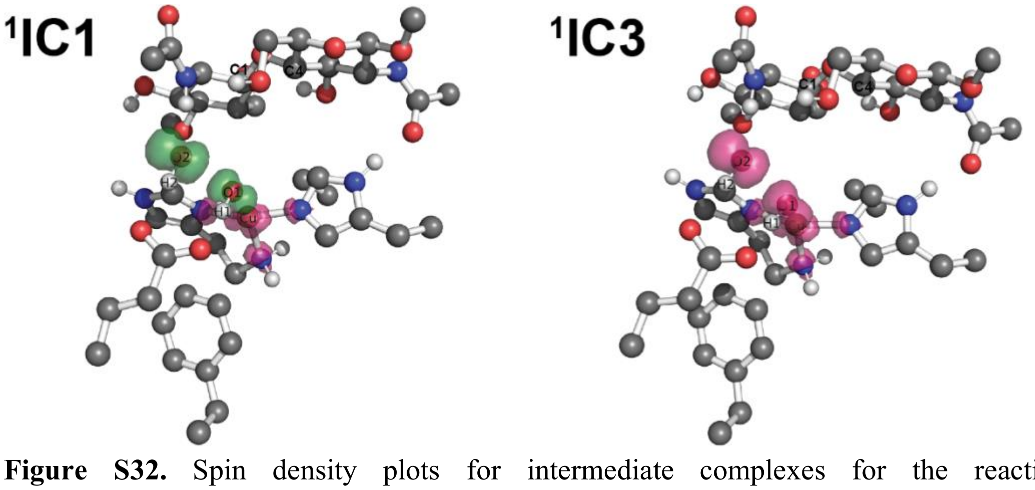

Figure S32. Spin density plots for intermediate complexes for the reaction with **Cu(I)-*Sm*AA10A·chitin·H_2O_2**. Net positive spin is indicated by magenta and net negative spin by forest green. Spin population values are listed in Table S13.

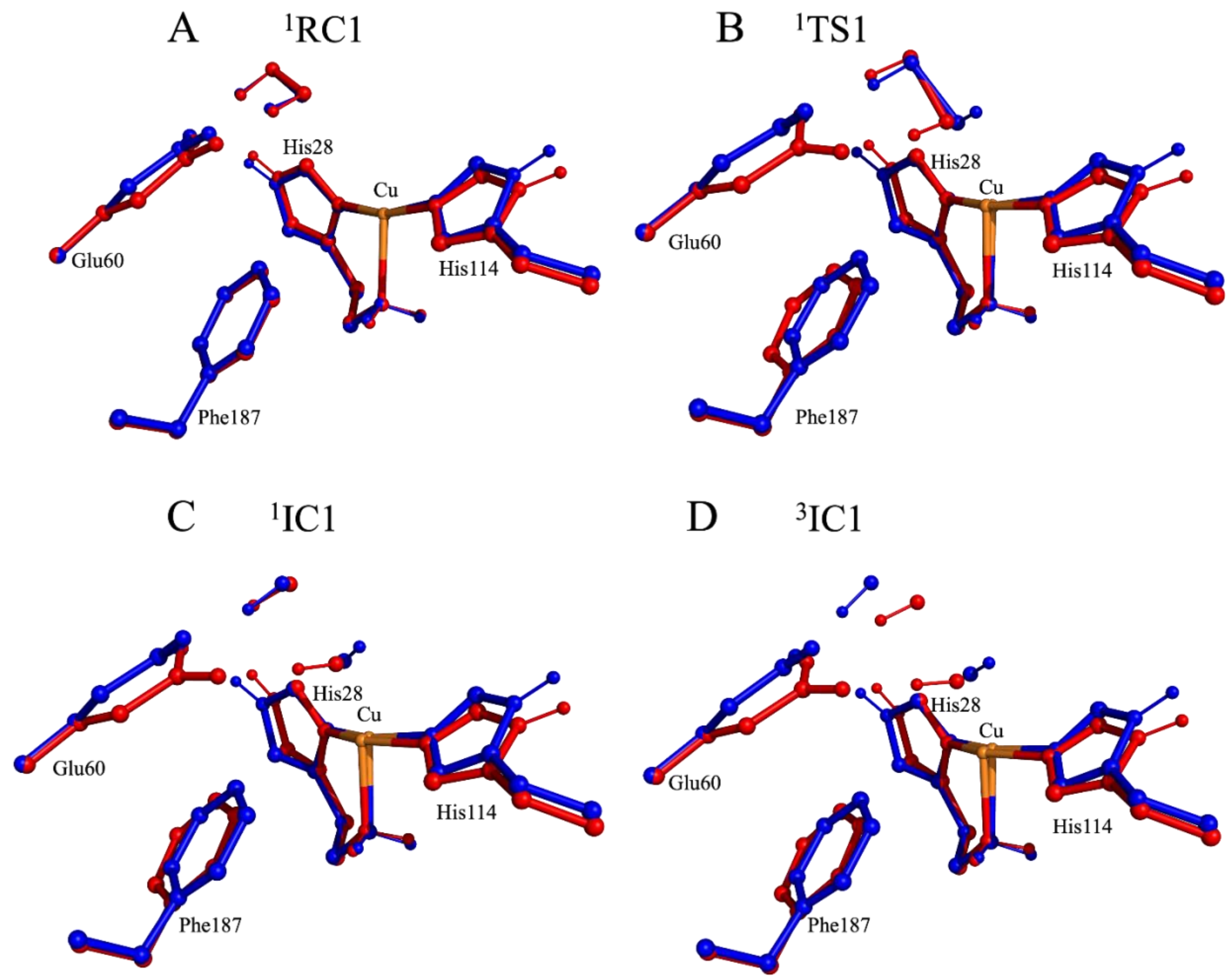

Figure S33. Overlay of optimized structures for **Cu(I)-*Sm*AA10A·H_2O_2** (blue) and **Cu(I)-*Sm*AA10A·chitin·H_2O_2** (red) from the H_2O_2 activation study. Structures were aligned using the Cα of Glu60 and the terminal N atom of His28. A) ^{1}RC B) ^{1}TS1 C) ^{1}IC1 D) ^{3}IC1. The overlay shows shortening of the Cu–Glu60 distance in the chitin-bound structure.

Coordinates for Cu(II)-*Sm*AA10A

N	2.099938794	-0.025456101	-0.180942108
C	2.857562239	1.251784707	-0.165079066
C	2.338470365	2.187963367	0.930334163
C	1.010124485	2.758905314	0.549803595
C	0.558868972	4.066810360	0.567206285
N	0.000000000	1.975754286	0.000000000
C	-1.033024156	2.778876927	-0.303516256
N	-0.713428125	4.049952494	0.025359526
H	2.728469309	1.742667610	-1.129764503
H	3.911854963	1.039174767	0.017307515
H	2.285296777	1.661252122	1.907678439
H	3.050717859	3.017021419	1.082689845
H	1.022539022	4.991196561	0.914927775
H	-1.981215353	2.460626321	-0.748730815
H	-1.345214587	4.868021326	-0.027130986
H	2.474519784	-0.694053410	0.518635421
H	2.269060491	-0.487302498	-1.087104763
C	-0.330888031	4.096093859	-6.267127109
C	-1.168144167	3.635091190	-5.065573240
C	-1.842887450	2.265431502	-5.248982183
C	-2.560225626	1.832752698	-3.954700531
O	-2.446105355	0.610618946	-3.579683659
O	-3.222855177	2.690248497	-3.303497773
H	-0.993055232	4.443448794	-7.059755741
H	0.327831166	4.909230903	-5.961506157
H	0.268328438	3.262405171	-6.634347470
H	-0.496001846	3.569850284	-4.189223809
H	-1.929824813	4.403616411	-4.832368228
H	-2.603375895	2.290048429	-6.062374052
H	-1.106764010	1.479821160	-5.520291366
C	3.015315534	-1.464665429	3.590655290
C	1.629614031	-0.815170191	3.519428316
H	3.163819370	-1.896851762	4.580379284
H	3.089040147	-2.249473546	2.837675940
H	3.781208474	-0.711422979	3.406362549
H	1.558872480	-0.036260582	4.299059288
H	1.478036501	-0.353656325	2.529034857
H	0.845654024	-1.578075787	3.684506355
C	1.610421069	-6.419166132	-0.500775551
C	0.904007402	-5.362297363	-1.367656245
C	0.483628056	-4.125663832	-0.636432125
C	0.709992621	-2.796638751	-0.958701295
N	-0.298630400	-4.090937348	0.507564283
C	-0.520585936	-2.796746558	0.846114042
N	0.070240947	-1.985030237	-0.034266108
H	1.629786487	-6.084128519	0.536326555
H	1.071571112	-7.364208748	-0.567259962
H	2.632123194	-6.557832666	-0.856503480
H	1.599469066	-5.056004215	-2.168017778
H	0.031137913	-5.822841474	-1.870578682
H	-0.625982308	-4.891611304	1.072773045
H	1.263123982	-2.411902144	-1.821750217

622	H	-1.107042996	-2.506362777	1.719691959
623	C	2.992547969	1.602543404	-7.654668921
624	C	3.664155716	1.423605668	-6.291071355
625	C	2.679097489	1.100237522	-5.188433004
626	C	2.219722461	-0.220315351	-4.993621065
627	C	2.221287191	2.116235824	-4.320735372
628	C	1.287162098	-0.504640687	-3.985585215
629	C	1.285201186	1.831163432	-3.312807932
630	C	0.799850682	0.522615088	-3.158470256
631	H	2.008612178	1.134367272	-7.638437210
632	H	2.886266339	2.665531355	-7.870524964
633	H	3.604411372	1.133146979	-8.425314866
634	H	4.426384359	0.631350269	-6.387797143
635	H	4.210632409	2.351575311	-6.032120462
636	H	2.608748638	-1.038644843	-5.622979054
637	H	2.606713056	3.142393284	-4.435773494
638	H	0.945218826	-1.541014498	-3.830623538
639	H	0.943887387	2.628679100	-2.634263424
640	H	0.044798421	0.301674659	-2.391944711
641	Cu	0.000000000	0.000000000	0.000000000
642	O	-1.206248338	0.221478452	1.894132583
643	H	-1.081106265	1.118198257	2.345137075
644	H	-1.431196941	-0.384107480	2.636638345
645	O	-1.926807940	0.025753202	-1.014696474
646	H	-2.127605566	0.288049989	-1.974107079
647	H	-2.563881204	-0.725901500	-0.842932492

Coordinates for Cu(II)-*Sm*AA10A·chitin

C	-7.305539262	-6.256443944	1.894376911
O	-6.016621304	-5.522677039	1.819835474
H	-8.124499068	-5.542842313	1.984796499
H	-7.439454293	-6.849604568	0.989761708
H	-7.300032201	-6.914329234	2.763431355
C	-2.940938019	-6.276864151	4.987164464
C	-5.998273530	-4.070997641	2.113974924
C	-4.609940536	-3.703989858	2.704707772
C	-3.195722274	-5.424939728	3.763218560
N	-4.225881722	-4.541648602	3.829464301
O	-2.502775959	-5.549179813	2.724017340
C	-4.608822429	-2.214546609	3.077158792
O	-3.314287105	-1.857562423	3.546114039
C	-5.002190850	-1.391906015	1.843439670
O	-5.257547418	-0.025254185	2.173898441
C	-6.272910338	-1.904865288	1.131527798
O	-6.223661633	-3.333672032	0.926883382
C	-6.471091589	-1.212866488	-0.214457980
O	-5.271183722	-1.253601784	-0.989755591
H	-6.778534630	-3.844373036	2.882897400
H	-3.862866821	-3.883740651	1.905017187
H	-5.368678824	-2.042019850	3.875278684
H	-4.157559320	-1.453749260	1.122809150
H	-7.156771182	-1.652033478	1.764398872
H	-1.851255594	-6.285591702	5.188106629
H	-3.233158084	-7.324177448	4.758034411
H	-4.780462291	-4.443494332	4.697260446
H	-3.489896017	-5.927874135	5.883051752
H	-3.406797139	-0.946751004	3.928321168
H	-7.307389624	-1.721492073	-0.744388349
H	-6.789075405	-0.168672567	-0.004851872
H	-5.449022006	-0.793781649	-1.867072573
C	-6.336229761	2.810669538	-1.684971892
C	-4.183364450	0.863688577	2.159382192
C	-4.643868293	2.241873936	1.673004880
C	-6.165452763	2.829978345	-0.180851511
N	-5.070582156	2.164935019	0.291004787
O	-6.971880309	3.399088606	0.568031915
C	-3.507230199	3.293889732	1.796148320
O	-4.033507830	4.575102929	1.498414183
C	-2.744532094	3.249492060	3.137211454
O	-1.412001880	3.785907840	3.011397212
C	-2.474884199	1.781981882	3.552107424
O	-3.659215681	0.970989989	3.482641393
C	-1.890890965	1.638327191	4.950915436
O	-2.821444164	2.039140873	5.936662373
H	-3.375448839	0.486761786	1.478889450
H	-5.517295686	2.564923362	2.273797773
H	-2.747475458	3.004169898	1.039866748
H	-3.344576105	3.734312782	3.942716848
H	-1.711122180	1.388156938	2.833118478
H	-6.659204463	3.819773693	-1.988190556

702	H	-7.144719851	2.104091370	-1.962493465
703	H	-4.407805761	1.725634823	-0.368253474
704	H	-5.416510515	2.545615485	-2.238478893
705	H	-3.552454953	4.930906714	0.705863365
706	H	-1.590188965	0.568835559	5.078798173
707	H	-0.967583074	2.259653295	4.963314566
708	H	-2.317041293	2.101925805	6.795749380
709	C	-1.342105739	5.250104498	3.256439358
710	H	-1.936318480	5.499558457	4.135522184
711	H	-0.305428803	5.541859647	3.424599363
712	H	-1.734255846	5.783158807	2.390313213
713	N	2.064495191	-0.167780772	0.002977130
714	H	2.365660469	-0.767841014	0.790596381
715	H	2.261212142	-0.719584925	-0.849052487
716	C	2.886112803	1.076679605	-0.000552446
717	H	2.767854364	1.509206470	-1.017925491
718	C	2.308159413	2.087647966	1.009438139
719	H	2.181724213	1.610577211	2.007604824
720	H	3.040071281	2.896717441	1.151124079
721	C	1.026298524	2.707941230	0.547689041
722	N	0.000000000	1.944865728	0.000000000
723	C	-0.973423413	2.781932269	-0.397417529
724	H	-1.908799530	2.510651461	-0.890985644
725	N	-0.611952494	4.053263343	-0.113285491
726	H	-1.190796129	4.879773036	-0.341315709
727	C	0.632466497	4.037936757	0.488029041
728	H	1.137338292	4.948187081	0.820723954
729	C	-0.984878752	2.979889002	-5.070922766
730	H	-0.295234378	3.018702989	-4.207746906
731	H	-1.637583310	3.872974476	-5.000421957
732	C	-1.844636384	1.705018729	-4.949373001
733	H	-2.562462004	1.597993483	-5.797371729
734	H	-1.216401727	0.793110823	-4.922566791
735	C	-2.688101025	1.798035726	-3.662380957
736	O	-3.307965304	2.880694160	-3.453867534
737	O	-2.725293356	0.789991615	-2.870884957
738	C	1.267973850	-1.022244808	3.613101864
739	H	1.303337133	-0.816770341	2.530661907
740	H	0.432663414	-1.715286458	3.824279509
741	H	1.108386703	-0.075976851	4.160570355
742	C	0.316748666	-5.554830201	0.023077761
743	H	1.133746628	-5.525860009	-0.721126734
744	H	-0.554810058	-6.036392458	-0.463473537
745	C	-0.008208724	-4.144959546	0.393264433
746	N	-0.892687725	-3.756719851	1.383934773
747	H	-1.329212770	-4.376314539	2.125798702
748	C	-0.981536971	-2.406545492	1.389283649
749	H	-1.608579622	-1.862162496	2.108576858
750	N	-0.188274656	-1.898022097	0.431000374
751	C	0.437349237	-2.967411077	-0.188458292
752	H	1.148301922	-2.848135010	-1.014593018
753	C	3.664105305	-0.094417951	-5.915618598
754	H	4.295745938	-1.000930010	-5.877190033
755	H	4.325231016	0.784877105	-5.781799971

756	C	2.630390541	-0.113685488	-4.811510819
757	C	2.024309605	-1.318834571	-4.396019461
758	H	2.378821108	-2.276054448	-4.811648921
759	C	0.980818001	-1.302498680	-3.459369787
760	H	0.491999083	-2.240168160	-3.161857848
761	C	0.551262894	-0.089191211	-2.898286623
762	H	-0.322300360	-0.066800378	-2.228822075
763	C	1.206808744	1.104007614	-3.235442727
764	H	0.914042855	2.050321640	-2.756349444
765	C	2.229647237	1.089398568	-4.193554659
766	H	2.722584145	2.025890738	-4.494844139
767	Cu	0.000000000	0.000000000	0.000000000
768	O	-2.072404140	-0.004029152	-0.468609777
769	H	-2.359043430	0.443575710	-1.338184634
770	H	-2.305503221	-0.939410614	-0.654605946
771	H	3.934027365	0.844414684	0.189258211
772	C	-0.135064026	3.097213934	-6.349848107
773	H	0.056222322	4.148612466	-6.564492339
774	H	-0.670892864	2.646847196	-7.185406619
775	H	0.812480148	2.578397817	-6.204627403
776	C	2.598370491	-1.653373037	4.064013595
777	H	2.936248026	-2.366398856	3.311994480
778	H	2.453624101	-2.167683320	5.014084782
779	H	3.348079323	-0.871500684	4.185307504
780	C	0.770239102	-6.436761763	1.201272363
781	H	0.935240082	-7.455600152	0.850783148
782	H	0.000217064	-6.437531119	1.972741524
783	H	1.697714331	-6.040746887	1.614868109
784	C	3.031719463	-0.016610791	-7.317629241
785	H	2.672502087	-1.003286450	-7.610090118
786	H	3.777511336	0.326440140	-8.034713852
787	H	2.196214820	0.683200253	-7.300388714

Optimized Coordinates for Cu(I)-*Sm*AA10A

N	2.196416684	0.000000000	0.000000000
C	2.826225478	1.337813999	0.093101192
C	2.033706199	2.286244928	1.011085815
C	0.780684617	2.785398217	0.371607008
C	0.417867199	4.054129770	-0.014065282
N	-0.248411732	1.906395938	0.018817748
C	-1.199243770	2.640645452	-0.564403124
N	-0.825850258	3.938925348	-0.600694823
H	1.780638411	1.760543265	1.946570380
H	2.667669142	3.141457855	1.283312505
H	0.926723503	5.006545757	0.076163958
H	-2.137243181	2.272912356	-0.963939055
H	-1.378679093	4.701153219	-0.983584576
H	2.886561261	1.752798294	-0.919502987
H	3.840845690	1.219643345	0.507151587
H	2.509423147	-0.580441816	0.782171662
H	2.532548008	-0.464758767	-0.847387739
C	1.211190717	3.773623191	-7.093525542
C	0.145715022	3.507140270	-5.995068674
C	-0.509869391	2.130546284	-6.113635924
C	-1.519933839	1.742222735	-5.007986886
O	-1.919171008	2.646000357	-4.211217624
O	-1.887065880	0.524981355	-4.989580931
H	0.602338971	3.598009711	-4.999587284
H	-0.639565988	4.277453336	-6.034048818
H	-1.034866796	2.048704766	-7.082479616
H	0.266394386	1.346454832	-6.134343377
H	1.136993095	3.001000998	-7.871529885
H	2.213779008	3.747571404	-6.645565338
H	1.036085535	4.761157699	-7.540657457
C	1.845048273	-1.478856849	4.183230809
C	0.645578555	-0.883190179	3.403052971
H	-0.180915088	-1.601798832	3.314051306
H	0.242779572	0.012477679	3.897152512
H	0.916051808	-0.585291465	2.380382791
H	2.202252743	-0.748406434	4.920287856
H	2.655452664	-1.718636522	3.482653106
H	1.526238987	-2.393025861	4.700077643
C	1.147105099	-6.344860573	0.580494583
C	0.750877089	-5.396329242	-0.580149815
C	0.138368937	-4.088007968	-0.200145417
C	0.526522536	-2.810285103	-0.531453761
N	-1.018791089	-3.922284457	0.540162196
C	-1.296742847	-2.596613990	0.633495367
N	-0.369311767	-1.885885176	-0.010433685
H	1.629008747	-5.161161547	-1.197266593
H	0.033221209	-5.921966741	-1.233366490
H	-1.581983133	-4.666724320	0.943695539
H	1.384984540	-2.508093325	-1.120081584
H	-2.152328665	-2.198720977	1.167149992
H	1.520312027	-5.752507841	1.427334348
H	1.931655401	-7.031777310	0.240226508

842	H	0.268053052	-6.921580033	0.896616745
843	C	4.810562506	1.019239479	-6.787848896
844	C	5.121979084	0.887989479	-5.281501492
845	C	3.845082951	0.882177829	-4.471173331
846	C	3.136837564	-0.314567346	-4.271275299
847	C	3.287251009	2.071773262	-3.977012857
848	C	1.901701192	-0.321790841	-3.616672941
849	C	2.055433945	2.068210310	-3.314987208
850	C	1.352878800	0.872708006	-3.142022099
851	H	5.686256355	-0.040117274	-5.101378857
852	H	5.765464304	1.723275994	-4.964555086
853	H	3.558587454	-1.252253884	-4.642131025
854	H	3.825840884	3.012531582	-4.115468665
855	H	1.364543061	-1.261923801	-3.477321635
856	H	1.643370067	3.001717545	-2.928627247
857	H	0.389660713	0.869913032	-2.630379185
858	H	4.268217737	1.957443844	-6.969672684
859	H	4.192153809	0.169885414	-7.110001902
860	H	5.751233963	1.023347539	-7.357001258
861	Cu	0.000000000	0.000000000	0.000000000

Optimized Coordinates for Cu(I)-*Sm*AA10A·chitin

N	2.199141543	-0.255891803	0.001029910
C	2.984320752	0.993696639	0.040612557
C	2.328278622	2.037488533	0.958676127
C	1.127437969	2.672183197	0.336219455
C	0.894332644	3.978347101	-0.024582139
N	0.000000000	1.916464728	0.000000000
C	-0.878806302	2.756771305	-0.551283856
N	-0.367777910	4.007204366	-0.581534433
H	2.035674093	1.556268018	1.906524753
H	3.063052368	2.816120582	1.205910185
H	1.504463051	4.869109291	0.066282816
H	-1.854959090	2.494979716	-0.941759475
H	-0.841865789	4.828754322	-0.946381722
H	3.045169794	1.388749187	-0.980566261
H	4.015646594	0.809730355	0.386787206
H	2.399500831	-0.815129571	0.834783576
H	2.491302938	-0.820955560	-0.800222283
C	0.434393051	-6.435875162	0.671025132
C	0.127698000	-5.465002149	-0.491360448
C	-0.286887771	-4.082271416	-0.107311195
C	0.129477019	-2.865047236	-0.601601631
N	-1.240324503	-3.778337145	0.845232919
C	-1.375718301	-2.431863915	0.905265832
N	-0.554741538	-1.836946064	0.034394956
H	1.020924509	-5.353043373	-1.123539460
H	-0.643687041	-5.912259779	-1.141986379
H	-1.741246440	-4.455100967	1.456988095
H	0.875265838	-2.671335546	-1.364513412
H	-2.027963474	-1.936912799	1.616931809
H	0.892456556	-5.880186000	1.502680627
H	1.132316160	-7.215776888	0.341600022
H	-0.478325975	-6.907977832	1.055904948
C	1.512478568	3.512214399	-7.148926458
C	0.455154044	3.386168815	-6.034756891
C	-0.048588005	1.950114995	-5.885925635
C	-1.144163633	1.699053872	-4.827436326
O	-1.445399461	2.642213763	-4.033210285
O	-1.668695328	0.540236089	-4.832373400
H	0.859807423	3.742931419	-5.075917185
H	-0.406825762	4.036074605	-6.258702176
H	-0.439621835	1.596697936	-6.855685704
H	0.791786177	1.277459735	-5.644806352
H	1.332107366	2.743014733	-7.915135025
H	2.511199219	3.311427212	-6.730137176
H	1.529701329	4.506121570	-7.615874457
C	1.770191994	-1.641143076	4.188271371
C	0.647670048	-0.929157773	3.433888916
H	0.280214525	-1.534149563	2.595871131
H	-0.207482853	-0.734555314	4.092087753
H	0.978953978	0.033386118	3.019065967
H	2.226457612	-0.947062793	4.908913060
H	2.565055473	-1.965000813	3.499746396

916	H	1.378508977	-2.529633032	4.702277143
917	C	4.772556779	0.360714985	-6.874175783
918	C	5.098217270	0.212455969	-5.373838146
919	C	3.849398758	0.314166795	-4.528255715
920	C	3.050916552	-0.818263568	-4.300435716
921	C	3.415911059	1.545864933	-4.012170255
922	C	1.853485202	-0.722527030	-3.586324317
923	C	2.221485941	1.644905778	-3.292544811
924	C	1.434557702	0.510355022	-3.081170744
925	H	5.582128943	-0.761089749	-5.198393568
926	H	5.815619140	0.992282449	-5.075236255
927	H	3.373000785	-1.786520894	-4.692081933
928	H	4.023888986	2.438576235	-4.178732090
929	H	1.241739277	-1.612748212	-3.430108065
930	H	1.899608671	2.611139402	-2.900858212
931	H	0.497879229	0.585341379	-2.526654463
932	H	4.338964126	1.354339376	-7.059054480
933	H	4.040754718	-0.400468667	-7.178495232
934	H	5.694489486	0.259014249	-7.463973743
935	Cu	0.000000000	0.000000000	0.000000000
936	C	-7.349655306	-5.776379300	1.494296469
937	O	-6.011423560	-5.301283826	1.724865107
938	H	-7.837083836	-5.215803846	0.682899885
939	H	-7.259337306	-6.830849964	1.210078597
940	H	-7.951933699	-5.690484110	2.413975291
941	C	-2.833010013	-6.453459846	4.788686346
942	C	-5.961074490	-4.003128528	2.221303066
943	C	-4.537170381	-3.734588373	2.739057327
944	C	-3.097886976	-5.542757894	3.615048500
945	N	-4.117082629	-4.666528067	3.760153253
946	O	-2.402625724	-5.614394286	2.579921135
947	C	-4.454301003	-2.270325141	3.226496596
948	O	-3.111742503	-1.997460166	3.623487942
949	C	-4.917093814	-1.333508646	2.105115824
950	O	-5.070225853	0.029520487	2.569395467
951	C	-6.302337442	-1.726502022	1.574921927
952	O	-6.254603451	-3.100012342	1.150832317
953	C	-6.771321273	-0.886386642	0.404730866
954	O	-5.762176755	-0.874398326	-0.613530790
955	H	-6.711491842	-3.856960449	3.029731280
956	H	-3.862425398	-3.869016147	1.882819157
957	H	-5.139693068	-2.143334771	4.088116019
958	H	-4.190180078	-1.352206841	1.277827963
959	H	-7.041703792	-1.613569177	2.391993251
960	H	-1.803394066	-6.289941047	5.136097179
961	H	-2.906963460	-7.496106795	4.449951376
962	H	-4.630643433	-4.674225396	4.638415920
963	H	-3.526488120	-6.293561336	5.623007328
964	H	-3.076700675	-1.048716985	3.900652281
965	H	-7.712446753	-1.320476796	0.025692121
966	H	-6.980774110	0.130360040	0.772397535
967	H	-6.096523549	-0.336155356	-1.352258388
968	C	-6.578615474	2.765466295	-0.904500666
969	C	-3.954847836	0.849576599	2.512827956

970	C	-4.387213962	2.287241084	2.180218294
971	C	-6.225743750	2.696429235	0.566005098
972	N	-4.941334718	2.346992750	0.841296941
973	O	-7.062313372	2.941928209	1.450687327
974	C	-3.221898524	3.272143752	2.306089449
975	O	-3.718743395	4.598488206	2.144269863
976	C	-2.388222065	3.091853041	3.568945824
977	O	-1.170843663	3.821852515	3.367430100
978	C	-2.078965488	1.605737208	3.830729856
979	O	-3.297528205	0.824328526	3.796359433
980	C	-1.432864114	1.373531612	5.184150533
981	O	-2.222020168	1.993471017	6.207560690
982	H	-3.234262618	0.491927073	1.746307169
983	H	-5.194697694	2.565694844	2.874763252
984	H	-2.518555143	3.046498684	1.475733372
985	H	-2.938462304	3.502655611	4.431797794
986	H	-1.392131434	1.241808935	3.043221074
987	H	-6.940998064	3.777463674	-1.132680621
988	H	-7.402771227	2.066672779	-1.105807725
989	H	-4.305627076	2.188775440	0.062906495
990	H	-5.735079814	2.528431420	-1.564493929
991	H	-2.939756505	5.182232388	2.078984265
992	H	-1.358244945	0.287921807	5.360852575
993	H	-0.409922783	1.786511854	5.156547407
994	H	-1.780435867	1.823353565	7.058325781
995	C	-0.778430482	4.646951007	4.471532108
996	H	-0.541996819	4.049775498	5.366949828
997	H	0.120636712	5.186606544	4.149353386
998	H	-1.570054293	5.372000690	4.723336454

Optimized Coordinates for Cu(I)-*Sm*AA10A+H_2O_2 (BP86/def2-TZVP)

N	2.469799	-0.026487	-0.050358
C	3.082447	1.242094	0.408507
C	2.170854	2.001146	1.389977
C	1.019864	2.661525	0.706292
C	0.715714	3.993202	0.552102
N	0.048883	1.908398	0.038797
C	-0.811257	2.775285	-0.503718
N	-0.433497	4.040073	-0.211234
H	1.789099	1.299156	2.149672
H	2.761800	2.760481	1.920774
H	1.207345	4.889823	0.910736
H	-1.674828	2.526787	-1.118716
H	-0.924613	4.882220	-0.498555
H	3.283857	1.858082	-0.475418
H	4.029461	1.008469	0.921999
H	2.667099	-0.765956	0.628577
H	2.922692	-0.315962	-0.921041
C	2.488546	5.177815	-6.250795
C	1.280793	4.720389	-5.389638
C	0.653205	3.419556	-5.892616
C	-0.430697	2.806666	-5.005865
O	-0.945779	3.519897	-4.084412
O	-0.764682	1.600419	-5.253225
H	1.593858	4.586592	-4.345173
H	0.509452	5.504885	-5.370665
H	0.211190	3.572887	-6.892960
H	1.430526	2.649715	-6.029746
H	2.522566	4.588064	-7.177274
H	3.418571	5.027703	-5.686548
H	2.378821	6.241679	-6.498200
C	1.536789	-2.337272	3.637237
C	0.458551	-1.554721	2.845077
H	-0.348367	-2.212985	2.494389
H	-0.007938	-0.770514	3.458277
H	0.869290	-1.057628	1.955134
H	1.788662	-1.788763	4.553820
H	2.436673	-2.449808	3.018668
H	1.147834	-3.329171	3.900540
C	1.341798	-6.316807	-0.974715
C	1.112514	-5.134381	-1.951150
C	0.454719	-3.916187	-1.390237
C	0.887666	-2.610182	-1.384526
N	-0.796746	-3.873362	-0.801419
C	-1.083770	-2.588831	-0.468224
N	-0.072712	-1.788339	-0.809617
H	2.068639	-4.802588	-2.378958
H	0.492300	-5.489200	-2.792222
H	-1.412986	-4.667774	-0.648640
H	1.821881	-2.217944	-1.769356
H	-2.006798	-2.285227	0.012353
H	1.594027	-5.926758	0.021052
H	2.165251	-6.941027	-1.342750

1053	H	0.426504	-6.919616	-0.910440
1054	C	6.006549	2.311061	-6.048505
1055	C	6.104683	1.857663	-4.576060
1056	C	4.726339	1.721872	-3.967700
1057	C	3.995666	0.531657	-4.120867
1058	C	4.105457	2.799895	-3.317197
1059	C	2.681194	0.426519	-3.656797
1060	C	2.793201	2.696539	-2.844532
1061	C	2.072263	1.512583	-3.021860
1062	H	6.637100	0.895509	-4.521298
1063	H	6.698709	2.587646	-4.004725
1064	H	4.464114	-0.321419	-4.618340
1065	H	4.658758	3.732425	-3.181136
1066	H	2.129096	-0.505659	-3.791078
1067	H	2.332896	3.541520	-2.330024
1068	H	1.048205	1.431228	-2.655142
1069	H	5.496079	3.282770	-6.099614
1070	H	5.438008	1.567473	-6.624236
1071	H	7.017407	2.405346	-6.470517
1072	Cu	0.288268	0.042616	-0.353123
1073	O	-2.145006	0.807967	-3.164117
1074	O	-2.835587	2.103823	-2.964874
1075	H	-2.130344	2.740477	-3.366476
1076	H	-1.622208	1.017957	-4.023772

Optimized Coordinates for Cu(I)-*Sm*AA10A·chitin+H_2O_2 (BP86/def2-TZVP)

N	2.195511	0.028538	-0.022852
C	2.830198	1.359691	0.044343
C	2.038657	2.311712	0.955721
C	0.784978	2.805841	0.314138
C	0.407483	4.080018	-0.038328
N	-0.243336	1.929124	-0.040846
C	-1.209472	2.665945	-0.595699
N	-0.843688	3.967222	-0.609271
H	1.786627	1.791484	1.894657
H	2.672125	3.169111	1.222288
H	0.909900	5.034117	0.067930
H	-2.141747	2.293795	-1.015644
H	-1.404993	4.731660	-0.974167
H	2.865575	1.770115	-0.971963
H	3.868758	1.292598	0.411056
H	2.443309	-0.513847	0.809212
H	2.565885	-0.489394	-0.823519
C	1.158860	-6.322585	0.578490
C	0.762348	-5.384484	-0.583957
C	0.180483	-4.063436	-0.198823
C	0.460586	-2.800257	-0.673997
N	-0.820887	-3.883677	0.735867
C	-1.114645	-2.563033	0.804339
N	-0.352196	-1.866441	-0.043808
H	1.648199	-5.162754	-1.197261
H	0.061591	-5.913688	-1.252713
H	-1.250476	-4.620913	1.331659
H	1.193070	-2.511914	-1.419723
H	-1.834509	-2.155520	1.506161
H	1.532594	-5.724032	1.422473
H	1.949693	-7.012435	0.258216
H	0.300726	-6.901715	0.942197
C	1.209578	3.748752	-7.157300
C	0.151996	3.489836	-6.063718
C	-0.189179	2.008188	-5.907238
C	-1.220106	1.661987	-4.830639
O	-1.673364	2.591293	-4.086081
O	-1.570146	0.438305	-4.740846
H	0.494819	3.894013	-5.099759
H	-0.775312	4.034148	-6.305048
H	-0.569223	1.607089	-6.862402
H	0.716981	1.422500	-5.681926
H	1.135384	2.970154	-7.931667
H	2.216364	3.666649	-6.719563
H	1.110361	4.740613	-7.616622
C	1.855278	-1.434028	4.151011
C	0.671415	-0.852329	3.378875
H	0.392194	-1.490436	2.531481
H	-0.212342	-0.762952	4.021857
H	0.895886	0.144961	2.974762
H	2.213101	-0.697291	4.884813
H	2.695523	-1.656657	3.476362

1131	H	1.560710	-2.366426	4.651914
1132	C	4.811309	0.999248	-6.837191
1133	C	5.123565	0.877589	-5.331628
1134	C	3.854547	0.848002	-4.509969
1135	C	3.173120	-0.360926	-4.294387
1136	C	3.285781	2.028175	-4.004733
1137	C	1.959011	-0.389519	-3.602933
1138	C	2.074745	2.002845	-3.306352
1139	C	1.405222	0.793108	-3.107456
1140	H	5.701638	-0.041830	-5.149681
1141	H	5.750729	1.725982	-5.017282
1142	H	3.600762	-1.290837	-4.677818
1143	H	3.801264	2.979049	-4.161020
1144	H	1.439927	-1.338221	-3.455855
1145	H	1.648986	2.930160	-2.919592
1146	H	0.458513	0.769588	-2.566248
1147	H	4.267615	1.936659	-7.024544
1148	H	4.179396	0.160501	-7.160893
1149	H	5.749950	1.010588	-7.409005
1150	Cu	-0.027028	0.025268	-0.056367
1151	C	-6.662178	-6.587854	1.249782
1152	O	-5.393898	-5.960662	1.510209
1153	H	-7.196719	-6.081276	0.432557
1154	H	-6.442750	-7.621735	0.960305
1155	H	-7.287778	-6.581618	2.157806
1156	C	-2.163388	-6.760560	4.628841
1157	C	-5.506339	-4.670034	2.016173
1158	C	-4.133956	-4.240697	2.563764
1159	C	-2.510957	-5.877306	3.455791
1160	N	-3.626827	-5.125486	3.587257
1161	O	-1.794261	-5.860501	2.433032
1162	C	-4.232971	-2.780698	3.061765
1163	O	-2.940015	-2.355713	3.488458
1164	C	-4.780547	-1.895013	1.936930
1165	O	-5.101924	-0.563499	2.406459
1166	C	-6.100376	-2.443573	1.377913
1167	O	-5.884255	-3.798457	0.946451
1168	C	-6.644199	-1.655923	0.203668
1169	O	-5.628480	-1.528985	-0.799542
1170	H	-6.283621	-4.620638	2.810845
1171	H	-3.432014	-4.287764	1.720258
1172	H	-4.945613	-2.742273	3.909687
1173	H	-4.041093	-1.820763	1.124017
1174	H	-6.862303	-2.424646	2.181771
1175	H	-1.163991	-6.485206	4.993345
1176	H	-2.114883	-7.802579	4.283533
1177	H	-4.151148	-5.198885	4.456074
1178	H	-2.883130	-6.685382	5.452789
1179	H	-3.022080	-1.411585	3.771559
1180	H	-7.525976	-2.190591	-0.189640
1181	H	-6.970301	-0.670303	0.570860
1182	H	-6.000833	-1.009297	-1.533221
1183	C	-6.807321	1.992586	-1.113684
1184	C	-4.089785	0.382338	2.377971

1185	C	-4.681470	1.762000	2.045568
1186	C	-6.501639	1.955556	0.368543
1187	N	-5.194270	1.763873	0.685438
1188	O	-7.391780	2.089728	1.225212
1189	C	-3.642642	2.875495	2.200453
1190	O	-4.288707	4.135819	2.036912
1191	C	-2.817595	2.783845	3.478515
1192	O	-1.689925	3.652339	3.305850
1193	C	-2.341705	1.341779	3.737821
1194	O	-3.459312	0.423358	3.674829
1195	C	-1.698394	1.175921	5.102246
1196	O	-2.574571	1.689359	6.113865
1197	H	-3.317809	0.118686	1.623576
1198	H	-5.536555	1.938572	2.715364
1199	H	-2.902180	2.740214	1.382971
1200	H	-3.428163	3.120540	4.332865
1201	H	-1.602198	1.067079	2.961872
1202	H	-7.267979	2.961301	-1.353499
1203	H	-7.546150	1.211787	-1.343604
1204	H	-4.521093	1.694353	-0.078049
1205	H	-5.917788	1.849294	-1.740755
1206	H	-3.582454	4.807194	1.989078
1207	H	-1.499159	0.105246	5.273585
1208	H	-0.731259	1.707233	5.097539
1209	H	-2.130317	1.568889	6.971675
1210	C	-1.419581	4.509883	4.421760
1211	H	-1.136379	3.938025	5.320140
1212	H	-0.581453	5.151266	4.122550
1213	H	-2.294760	5.137619	4.658025
1214	O	-2.838732	0.219446	-2.451217
1215	O	-3.496131	1.545457	-2.514849
1216	H	-2.818480	2.047057	-3.112637
1217	H	-2.361858	0.210173	-3.364450

Optimized Coordinates of ^{1}RC1 for Cu(I)-*Sm*AA10A+H_2O_2

N	2.475520	-0.019959	-0.048654
C	3.090270	1.241104	0.406682
C	2.187541	2.001524	1.388663
C	1.038034	2.665060	0.710035
C	0.744338	3.988606	0.560269
N	0.063080	1.923529	0.047545
C	-0.786968	2.790498	-0.481731
N	-0.406210	4.045117	-0.192683
H	1.808990	1.306472	2.143978
H	2.778883	2.753316	1.913333
H	1.242018	4.873502	0.915473
H	-1.652334	2.548842	-1.077825
H	-0.891155	4.883253	-0.474634
H	3.288596	1.851472	-0.470314
H	4.030589	1.006973	0.915108
H	2.652577	-0.752007	0.630975
H	2.921648	-0.317253	-0.909240
C	2.488670	5.175813	-6.253768
C	1.285303	4.723988	-5.390149
C	0.653452	3.429134	-5.889620
C	-0.434073	2.823684	-5.008166
O	-0.934630	3.523416	-4.084317
O	-0.780363	1.634658	-5.267043
H	1.601331	4.590991	-4.356151
H	0.523844	5.506679	-5.369909
H	0.215455	3.583565	-6.882409
H	1.421421	2.661469	-6.021574
H	2.516941	4.589926	-7.173782
H	3.413622	5.023497	-5.696887
H	2.382630	6.232760	-6.499262
C	1.545984	-2.334208	3.638979
C	0.470603	-1.550002	2.852789
H	-0.333374	-2.202376	2.508475
H	0.013704	-0.770001	3.465078
H	0.881056	-1.061313	1.970955
H	1.801485	-1.790162	4.548373
H	2.437093	-2.449324	3.021339
H	1.157054	-3.317927	3.902082
C	1.319582	-6.313733	-0.971547
C	1.091457	-5.132959	-1.945412
C	0.440620	-3.914400	-1.380741
C	0.875491	-2.619456	-1.361650
N	-0.810099	-3.872122	-0.803394
C	-1.090543	-2.596355	-0.464999
N	-0.084471	-1.802884	-0.789918
H	2.041465	-4.809028	-2.372189
H	0.471619	-5.483271	-2.777607
H	-1.426543	-4.658364	-0.661601
H	1.806698	-2.230024	-1.732744
H	-2.008714	-2.295536	0.009146
H	1.575105	-5.927140	0.016335
H	2.133780	-6.936695	-1.339956

1272	H	0.408597	-6.909118	-0.904267
1273	C	5.996960	2.296334	-6.063923
1274	C	6.098530	1.844278	-4.596527
1275	C	4.726744	1.713176	-3.982354
1276	C	4.003603	0.525342	-4.116946
1277	C	4.109319	2.792104	-3.348083
1278	C	2.697513	0.423713	-3.650359
1279	C	2.805780	2.691743	-2.873355
1280	C	2.091000	1.510975	-3.032396
1281	H	6.623771	0.887403	-4.544711
1282	H	6.693039	2.567552	-4.032852
1283	H	4.468525	-0.326424	-4.601263
1284	H	4.654652	3.721876	-3.229708
1285	H	2.152338	-0.505144	-3.767955
1286	H	2.347708	3.536234	-2.374443
1287	H	1.076782	1.433138	-2.666659
1288	H	5.493388	3.263069	-6.112975
1289	H	5.427630	1.559928	-6.633411
1290	H	6.999661	2.386257	-6.486740
1291	Cu	0.237421	0.045449	-0.348478
1292	O	-2.169862	0.826517	-3.170226
1293	O	-2.860883	2.102225	-2.987301
1294	H	-2.171368	2.740498	-3.353634
1295	H	-1.653210	1.013509	-4.013593

Optimized Coordinates of [1]TS1 for Cu(I)-*Sm*AA10A+H_2O_2

N	2.392471	0.018614	-0.055929
C	3.056557	1.267437	0.380774
C	2.159468	2.026675	1.359625
C	1.019779	2.699890	0.682090
C	0.772353	4.037110	0.559961
N	0.030580	2.008133	0.008461
C	-0.799443	2.911633	-0.494831
N	-0.382686	4.144892	-0.178455
H	1.773408	1.330594	2.110579
H	2.754336	2.771686	1.889740
H	1.298803	4.896474	0.935649
H	-1.667228	2.702640	-1.095157
H	-0.846717	5.002198	-0.437471
H	3.268673	1.871660	-0.497111
H	3.997950	1.017946	0.879272
H	2.491700	-0.688955	0.664332
H	2.863443	-0.343880	-0.878187
C	2.508334	5.234831	-6.266618
C	1.295456	4.786201	-5.406615
C	0.703135	3.493210	-5.968161
C	-0.362490	2.721443	-5.178687
O	-0.882471	3.251645	-4.153425
O	-0.652384	1.584804	-5.627438
H	1.600841	4.644319	-4.370559
H	0.535599	5.571390	-5.381728
H	0.279106	3.691141	-6.959233
H	1.515846	2.780821	-6.142481
H	2.535467	4.653546	-7.188618
H	3.430507	5.073283	-5.707344
H	2.410564	6.294097	-6.507534
C	1.479752	-2.314975	3.587212
C	0.412541	-1.519414	2.801226
H	-0.394720	-2.164754	2.451365
H	-0.040983	-0.739044	3.415455
H	0.829219	-1.030340	1.924394
H	1.736094	-1.777080	4.499980
H	2.372044	-2.433500	2.971956
H	1.082998	-3.297117	3.844362
C	1.240349	-6.270954	-1.042878
C	1.024272	-5.086478	-2.009844
C	0.382515	-3.865669	-1.450518
C	0.836075	-2.577859	-1.469779
N	-0.845385	-3.799988	-0.831376
C	-1.099624	-2.516894	-0.506481
N	-0.093355	-1.745049	-0.881645
H	1.977212	-4.766264	-2.432726
H	0.404388	-5.430825	-2.845015
H	-1.463966	-4.576959	-0.651245
H	1.758405	-2.206844	-1.880082
H	-1.996579	-2.193157	-0.007753
H	1.495375	-5.890818	-0.052228
H	2.051275	-6.897905	-1.411558

1350	H	0.324963	-6.860148	-0.981405
1351	C	5.995465	2.329622	-6.078988
1352	C	6.088980	1.869929	-4.613445
1353	C	4.716702	1.744789	-4.001892
1354	C	3.987340	0.561922	-4.144783
1355	C	4.106165	2.824202	-3.362170
1356	C	2.678655	0.466994	-3.684748
1357	C	2.800054	2.730367	-2.894688
1358	C	2.077634	1.556270	-3.066140
1359	H	6.607321	0.909085	-4.565010
1360	H	6.686885	2.586790	-4.045060
1361	H	4.448948	-0.289805	-4.632311
1362	H	4.657976	3.749202	-3.236682
1363	H	2.125895	-0.456169	-3.810998
1364	H	2.344323	3.575440	-2.394635
1365	H	1.056453	1.490404	-2.720186
1366	H	5.498913	3.300160	-6.125095
1367	H	5.422802	1.599971	-6.653833
1368	H	7.000138	2.414410	-6.498082
1369	Cu	0.168560	0.143787	-0.557743
1370	O	-1.457055	0.569794	-1.886683
1371	O	-2.558892	1.454372	-2.864253
1372	H	-1.894028	2.020825	-3.340801
1373	H	-1.627338	-0.282816	-2.314515

Optimized Coordinates of ^{1}IC1 for Cu(I)-*Sm*AA10A+H_2O_2

N	2.338044	0.019337	-0.032877
C	3.045014	1.267491	0.379659
C	2.147803	2.022431	1.349500
C	1.013135	2.692185	0.668866
C	0.754385	4.026114	0.538573
N	0.038723	1.993466	-0.001412
C	-0.803027	2.879003	-0.520728
N	-0.400519	4.115466	-0.204909
H	1.759567	1.326443	2.099233
H	2.739787	2.768479	1.881592
H	1.266813	4.893023	0.916102
H	-1.655661	2.640972	-1.135582
H	-0.872833	4.966561	-0.470122
H	3.266647	1.862425	-0.502180
H	3.990310	1.009792	0.862514
H	2.416165	-0.668523	0.709686
H	2.807919	-0.383467	-0.838417
C	2.523512	5.226511	-6.277181
C	1.308741	4.777424	-5.422478
C	0.703717	3.485485	-5.966754
C	-0.396795	2.778660	-5.163681
O	-0.880691	3.352256	-4.145898
O	-0.747808	1.650893	-5.592818
H	1.611891	4.636993	-4.385684
H	0.549555	5.563415	-5.402723
H	0.300505	3.665316	-6.969798
H	1.500804	2.747562	-6.104561
H	2.554969	4.644546	-7.198995
H	3.443673	5.066302	-5.714115
H	2.425542	6.285443	-6.519090
C	1.464009	-2.318666	3.576918
C	0.400132	-1.525543	2.787013
H	-0.405076	-2.171221	2.434135
H	-0.056768	-0.745291	3.398856
H	0.819452	-1.037776	1.913535
H	1.716115	-1.779961	4.490372
H	2.358905	-2.436567	2.965271
H	1.067329	-3.301067	3.833056
C	1.247420	-6.277612	-1.051758
C	1.034742	-5.098663	-2.016412
C	0.392485	-3.889874	-1.454395
C	0.917921	-2.631896	-1.414563
N	-0.833404	-3.789359	-0.842133
C	-1.022480	-2.509579	-0.465796
N	0.031157	-1.783513	-0.799525
H	1.986774	-4.774812	-2.437733
H	0.418145	-5.446583	-2.853296
H	-1.491554	-4.540527	-0.694861
H	1.863522	-2.298082	-1.803694
H	-1.907851	-2.154417	0.031543
H	1.498107	-5.896570	-0.060205
H	2.060627	-6.903981	-1.416894

1428	H	0.332400	-6.867813	-0.993582
1429	C	6.013071	2.325270	-6.073950
1430	C	6.101286	1.866371	-4.607240
1431	C	4.724109	1.741604	-4.005651
1432	C	3.984689	0.567997	-4.172459
1433	C	4.116842	2.818335	-3.357895
1434	C	2.667307	0.482827	-3.735345
1435	C	2.802176	2.734451	-2.913118
1436	C	2.067442	1.572972	-3.116408
1437	H	6.620560	0.906081	-4.556576
1438	H	6.695916	2.583539	-4.036062
1439	H	4.443898	-0.282627	-4.664132
1440	H	4.677146	3.735148	-3.211941
1441	H	2.105463	-0.431579	-3.883625
1442	H	2.348025	3.578937	-2.410686
1443	H	1.034536	1.520033	-2.803116
1444	H	5.515647	3.295209	-6.122605
1445	H	5.443536	1.594687	-6.650613
1446	H	7.019324	2.410926	-6.489093
1447	Cu	0.223933	0.143993	-0.608090
1448	O	-1.332270	0.436784	-1.691363
1449	O	-2.799198	1.772437	-2.839407
1450	H	-2.039158	2.212144	-3.302433
1451	H	-1.570562	-0.355185	-2.186184

Optimized Coordinates of ^{3}IC1 for Cu(I)-*Sm*AA10A+H_2O_2

N	2.346622	0.002292	-0.037309
C	3.041264	1.256838	0.375247
C	2.133341	2.007525	1.338343
C	0.995957	2.667027	0.649471
C	0.725317	3.998507	0.512648
N	0.030795	1.956857	-0.023544
C	-0.811048	2.832287	-0.554174
N	-0.422739	4.074911	-0.242450
H	1.746783	1.310738	2.088084
H	2.716866	2.759765	1.871281
H	1.228529	4.871981	0.887507
H	-1.662421	2.593311	-1.166142
H	-0.899814	4.920454	-0.516783
H	3.262348	1.851414	-0.506954
H	3.985788	1.008381	0.864551
H	2.444474	-0.690055	0.698521
H	2.810633	-0.386174	-0.853157
C	2.522594	5.192522	-6.295648
C	1.309144	4.737216	-5.447216
C	0.683577	3.442245	-5.953437
C	-0.476200	2.864035	-5.135250
O	-0.956938	3.579773	-4.199300
O	-0.884901	1.723722	-5.445277
H	1.612670	4.603437	-4.409684
H	0.546713	5.519254	-5.436027
H	0.313869	3.581950	-6.975741
H	1.445482	2.659477	-6.019999
H	2.563252	4.607880	-7.216058
H	3.441047	5.041122	-5.727547
H	2.417634	6.249690	-6.541065
C	1.472118	-2.332357	3.574925
C	0.406193	-1.549891	2.777447
H	-0.391801	-2.202832	2.422632
H	-0.059456	-0.771613	3.385322
H	0.826511	-1.061481	1.904682
H	1.715415	-1.789070	4.488081
H	2.370992	-2.445035	2.968120
H	1.081804	-3.317092	3.831928
C	1.309712	-6.306148	-1.043237
C	1.092998	-5.133075	-2.011214
C	0.438073	-3.940252	-1.435686
C	0.958377	-2.683322	-1.349114
N	-0.793878	-3.865001	-0.832661
C	-0.990583	-2.597770	-0.417608
N	0.063008	-1.858182	-0.716973
H	2.042981	-4.800698	-2.430435
H	0.483488	-5.485650	-2.851265
H	-1.449459	-4.623406	-0.714387
H	1.908227	-2.335724	-1.715084
H	-1.883641	-2.261334	0.079031
H	1.552401	-5.920343	-0.051619
H	2.129745	-6.927311	-1.402519

1506	H	0.398979	-6.903330	-0.987939
1507	C	6.033556	2.319173	-6.066441
1508	C	6.117927	1.865232	-4.598116
1509	C	4.739385	1.731056	-4.001855
1510	C	4.008675	0.550156	-4.151400
1511	C	4.123935	2.813642	-3.370950
1512	C	2.691976	0.462776	-3.711263
1513	C	2.810572	2.728261	-2.924072
1514	C	2.084898	1.558077	-3.108437
1515	H	6.644459	0.909106	-4.542281
1516	H	6.703983	2.588437	-4.025849
1517	H	4.473559	-0.305091	-4.629491
1518	H	4.678568	3.735957	-3.238819
1519	H	2.137256	-0.458479	-3.843101
1520	H	2.350845	3.576312	-2.432853
1521	H	1.056233	1.500064	-2.782936
1522	H	5.528856	3.285049	-6.120417
1523	H	5.472633	1.582505	-6.643838
1524	H	7.041185	2.411478	-6.476766
1525	Cu	0.208154	0.089794	-0.589407
1526	O	-1.353996	0.309890	-1.673332
1527	O	-3.131263	2.836148	-3.010096
1528	H	-2.258400	3.081704	-3.499929
1529	H	-1.550124	-0.535726	-2.090091

Optimized Coordinates of ^{1}RC1 for Cu(I)-*Sm*AA10A·chitin+H_2O_2

N	2.217098	0.018404	-0.009055
C	2.835414	1.349192	0.048386
C	2.047332	2.300419	0.957435
C	0.795724	2.795411	0.316808
C	0.416738	4.064659	-0.008691
N	-0.223970	1.926019	-0.057217
C	-1.182173	2.664354	-0.595217
N	-0.829025	3.959248	-0.583109
H	1.796900	1.785155	1.889041
H	2.677420	3.150769	1.221524
H	0.912896	5.010513	0.117427
H	-2.108966	2.299840	-1.006208
H	-1.389777	4.721097	-0.932362
H	2.866290	1.752983	-0.960439
H	3.868511	1.294009	0.409304
H	2.442884	-0.506303	0.829950
H	2.595760	-0.506436	-0.789454
C	1.155421	-6.332806	0.584126
C	0.760624	-5.396423	-0.576400
C	0.189141	-4.072595	-0.195352
C	0.479723	-2.819615	-0.661403
N	-0.822373	-3.890633	0.719259
C	-1.108981	-2.576865	0.782133
N	-0.338916	-1.889023	-0.046088
H	1.639965	-5.185031	-1.188078
H	0.057920	-5.919074	-1.234335
H	-1.253339	-4.616787	1.301488
H	1.218399	-2.534856	-1.389852
H	-1.832553	-2.167463	1.463418
H	1.527619	-5.738593	1.421856
H	1.939200	-7.018970	0.264616
H	0.302035	-6.906435	0.944617
C	1.217831	3.735276	-7.155929
C	0.162531	3.478838	-6.064307
C	-0.215308	2.007414	-5.938712
C	-1.246857	1.667988	-4.866008
O	-1.684672	2.587361	-4.119904
O	-1.610111	0.458546	-4.786511
H	0.519155	3.851216	-5.102695
H	-0.744901	4.046632	-6.288554
H	-0.615065	1.645066	-6.891803
H	0.668349	1.396719	-5.737500
H	1.142867	2.961985	-7.924467
H	2.217341	3.654678	-6.721255
H	1.118080	4.720132	-7.612246
C	1.859521	-1.443794	4.154255
C	0.678940	-0.862033	3.383927
H	0.413542	-1.488989	2.535280
H	-0.201305	-0.787647	4.019332
H	0.899651	0.133501	2.997365
H	2.216129	-0.712288	4.882676
H	2.693402	-1.666447	3.483949

1584	H	1.565566	-2.369132	4.652392
1585	C	4.815991	0.980909	-6.835707
1586	C	5.127495	0.859820	-5.335027
1587	C	3.863753	0.862847	-4.513159
1588	C	3.168767	-0.323972	-4.272237
1589	C	3.317286	2.057035	-4.039678
1590	C	1.959379	-0.316946	-3.586354
1591	C	2.110766	2.066737	-3.349225
1592	C	1.426441	0.879452	-3.124044
1593	H	5.683985	-0.062931	-5.152092
1594	H	5.768091	1.690795	-5.029627
1595	H	3.578699	-1.261647	-4.631274
1596	H	3.842731	2.988726	-4.217772
1597	H	1.429856	-1.246250	-3.416275
1598	H	1.703336	3.001887	-2.986386
1599	H	0.488289	0.883840	-2.588376
1600	H	4.277403	1.912351	-7.021962
1601	H	4.187057	0.148931	-7.156730
1602	H	5.748220	0.989472	-7.403604
1603	Cu	-0.054187	0.012121	-0.069588
1604	C	-6.643354	-6.580298	1.232821
1605	O	-5.390763	-5.947176	1.505223
1606	H	-7.175729	-6.071844	0.426523
1607	H	-6.416849	-7.601175	0.932440
1608	H	-7.268627	-6.592689	2.130861
1609	C	-2.174524	-6.759042	4.628931
1610	C	-5.501675	-4.669015	2.025171
1611	C	-4.133695	-4.242916	2.570109
1612	C	-2.526896	-5.882193	3.456393
1613	N	-3.628947	-5.125360	3.590803
1614	O	-1.822260	-5.875149	2.438856
1615	C	-4.230369	-2.781214	3.068597
1616	O	-2.940973	-2.364969	3.485659
1617	C	-4.780071	-1.896700	1.945684
1618	O	-5.095949	-0.568850	2.412996
1619	C	-6.098689	-2.441377	1.397844
1620	O	-5.890157	-3.792411	0.977391
1621	C	-6.646783	-1.660572	0.225684
1622	O	-5.638125	-1.518958	-0.771965
1623	H	-6.265435	-4.636627	2.819561
1624	H	-3.441910	-4.288565	1.731818
1625	H	-4.930956	-2.741193	3.913281
1626	H	-4.050687	-1.825639	1.137865
1627	H	-6.850830	-2.416729	2.197433
1628	H	-1.190184	-6.466496	4.998105
1629	H	-2.106656	-7.791828	4.284486
1630	H	-4.145865	-5.191655	4.454735
1631	H	-2.896958	-6.695834	5.441037
1632	H	-3.007069	-1.441828	3.792154
1633	H	-7.512780	-2.201653	-0.168457
1634	H	-6.982950	-0.686928	0.586016
1635	H	-6.009068	-1.025349	-1.512689
1636	C	-6.806218	1.992947	-1.091937
1637	C	-4.083287	0.379890	2.381146

1638	C	-4.673570	1.760191	2.049236
1639	C	-6.490471	1.961268	0.384358
1640	N	-5.192429	1.765487	0.696471
1641	O	-7.372167	2.101066	1.234060
1642	C	-3.632841	2.872637	2.203454
1643	O	-4.273419	4.126430	2.040544
1644	C	-2.810663	2.775099	3.477382
1645	O	-1.675479	3.621532	3.320801
1646	C	-2.346572	1.335205	3.739243
1647	O	-3.454101	0.420964	3.662680
1648	C	-1.716852	1.172253	5.106394
1649	O	-2.596313	1.685802	6.105147
1650	H	-3.325116	0.117857	1.629152
1651	H	-5.512390	1.936996	2.723206
1652	H	-2.900517	2.739387	1.394358
1653	H	-3.418020	3.111191	4.321500
1654	H	-1.604581	1.062741	2.979811
1655	H	-7.274583	2.950227	-1.326599
1656	H	-7.532750	1.208348	-1.311009
1657	H	-4.528872	1.690213	-0.062305
1658	H	-5.926482	1.857100	-1.719113
1659	H	-3.585312	4.797939	1.947323
1660	H	-1.522278	0.111746	5.282431
1661	H	-0.759580	1.701701	5.113249
1662	H	-2.184040	1.548441	6.966338
1663	C	-1.448141	4.536869	4.389261
1664	H	-1.239403	4.017827	5.328479
1665	H	-0.581498	5.133580	4.108499
1666	H	-2.310514	5.195356	4.529737
1667	O	-2.911658	0.228339	-2.496227
1668	O	-3.530296	1.552846	-2.538728
1669	H	-2.867490	2.051599	-3.114714
1670	H	-2.435522	0.207245	-3.384826

Optimized Coordinates of ^{1}TS1 for Cu(I)-*Sm*AA10A·chitin+H_2O_2

N	2.073888	-0.032297	-0.138889
C	2.796782	1.246717	-0.006127
C	2.025218	2.206177	0.898563
C	0.772225	2.706939	0.262799
C	0.406480	4.011884	0.090835
N	-0.247526	1.904340	-0.215102
C	-1.201294	2.711206	-0.660171
N	-0.838186	3.989488	-0.490374
H	1.777067	1.698725	1.835888
H	2.663227	3.053107	1.152551
H	0.914199	4.928874	0.332038
H	-2.125236	2.393609	-1.110338
H	-1.392319	4.792300	-0.746370
H	2.910749	1.679914	-0.995433
H	3.799128	1.091049	0.406852
H	2.181197	-0.574336	0.712927
H	2.484873	-0.586839	-0.882235
C	1.064423	-6.422133	0.588018
C	0.668481	-5.489330	-0.577089
C	0.144595	-4.133419	-0.242031
C	0.438571	-2.921913	-0.808565
N	-0.844347	-3.870530	0.677979
C	-1.118276	-2.553520	0.645539
N	-0.358897	-1.942630	-0.250660
H	1.538883	-5.323108	-1.215177
H	-0.064184	-6.004507	-1.208132
H	-1.270447	-4.546838	1.321492
H	1.157843	-2.698614	-1.577862
H	-1.833265	-2.086107	1.298420
H	1.446908	-5.825385	1.419256
H	1.839650	-7.116039	0.265403
H	0.209452	-6.986754	0.958430
C	1.148890	3.593357	-7.218155
C	0.096151	3.351470	-6.113792
C	-0.147476	1.865555	-5.842616
C	-1.100627	1.497349	-4.692100
O	-1.850136	2.384598	-4.201193
O	-1.086780	0.290994	-4.313961
H	0.407661	3.848188	-5.193035
H	-0.852031	3.813747	-6.402556
H	-0.561608	1.399240	-6.745152
H	0.797069	1.354567	-5.652766
H	1.062713	2.815339	-7.981170
H	2.150651	3.512825	-6.789317
H	1.045773	4.575820	-7.679754
C	1.830621	-1.515482	4.121202
C	0.648214	-0.929102	3.354355
H	0.372621	-1.559230	2.509481
H	-0.227925	-0.846854	3.994319
H	0.873365	0.063784	2.963310
H	2.197772	-0.782069	4.842290
H	2.657984	-1.748946	3.446756

1725	H	1.532722	-2.434952	4.627644
1726	C	4.729543	0.813985	-6.904512
1727	C	5.051220	0.700189	-5.401770
1728	C	3.907253	1.160169	-4.541653
1729	C	2.980481	0.259457	-4.017571
1730	C	3.715392	2.523765	-4.309461
1731	C	1.877364	0.711101	-3.305751
1732	C	2.615051	2.977326	-3.593589
1733	C	1.687134	2.070567	-3.099612
1734	H	5.318019	-0.330581	-5.157066
1735	H	5.927667	1.316528	-5.183723
1736	H	3.118206	-0.803337	-4.183783
1737	H	4.432622	3.235253	-4.703621
1738	H	1.151165	0.005295	-2.930201
1739	H	2.478222	4.039035	-3.427655
1740	H	0.819710	2.418774	-2.560357
1741	H	4.197002	1.747691	-7.093162
1742	H	4.081454	-0.005404	-7.221161
1743	H	5.659025	0.797218	-7.475262
1744	Cu	-0.193418	-0.025354	-0.453988
1745	C	-6.736267	-6.602447	1.296817
1746	O	-5.475283	-5.982811	1.561230
1747	H	-7.262124	-6.096955	0.484411
1748	H	-6.523459	-7.629887	1.008994
1749	H	-7.361446	-6.595569	2.194996
1750	C	-2.177469	-6.709788	4.698470
1751	C	-5.569009	-4.696612	2.065147
1752	C	-4.195003	-4.277931	2.597439
1753	C	-2.532569	-5.832785	3.526727
1754	N	-3.690088	-5.157423	3.620297
1755	O	-1.783440	-5.758149	2.544371
1756	C	-4.277722	-2.812133	3.085468
1757	O	-2.978352	-2.405366	3.483096
1758	C	-4.823523	-1.932379	1.958181
1759	O	-5.129395	-0.599232	2.416737
1760	C	-6.143557	-2.471363	1.408589
1761	O	-5.948807	-3.829468	1.006404
1762	C	-6.669337	-1.698255	0.220755
1763	O	-5.644142	-1.572409	-0.762677
1764	H	-6.330799	-4.644854	2.860483
1765	H	-3.508116	-4.335417	1.755645
1766	H	-4.969277	-2.758433	3.936447
1767	H	-4.091799	-1.873608	1.151346
1768	H	-6.902428	-2.427222	2.200874
1769	H	-1.213160	-6.388178	5.094611
1770	H	-2.065976	-7.735973	4.344988
1771	H	-4.235407	-5.266012	4.462056
1772	H	-2.921725	-6.681447	5.492714
1773	H	-3.033114	-1.481442	3.788226
1774	H	-7.532736	-2.237032	-0.181819
1775	H	-7.003993	-0.718789	0.566586
1776	H	-6.005813	-1.099159	-1.521084
1777	C	-6.768065	1.902092	-1.148936
1778	C	-4.110445	0.342458	2.376127

1779	C	-4.691840	1.723854	2.035858
1780	C	-6.479147	1.916044	0.333505
1781	N	-5.191162	1.709294	0.673065
1782	O	-7.373237	2.104434	1.161118
1783	C	-3.642450	2.829032	2.176738
1784	O	-4.274543	4.086454	2.009978
1785	C	-2.812493	2.734522	3.446532
1786	O	-1.670614	3.568662	3.275193
1787	C	-2.361047	1.292330	3.718214
1788	O	-3.478020	0.388348	3.656353
1789	C	-1.722409	1.133917	5.081730
1790	O	-2.589945	1.661446	6.083433
1791	H	-3.356198	0.070242	1.624410
1792	H	-5.535750	1.913916	2.699045
1793	H	-2.916549	2.683091	1.364167
1794	H	-3.410918	3.083914	4.291693
1795	H	-1.626488	1.007142	2.956081
1796	H	-7.172758	2.874870	-1.433813
1797	H	-7.536494	1.153785	-1.349988
1798	H	-4.520472	1.571725	-0.073619
1799	H	-5.886191	1.687692	-1.751489
1800	H	-3.582221	4.752278	1.907394
1801	H	-1.534879	0.073051	5.263615
1802	H	-0.760842	1.655597	5.077731
1803	H	-2.171682	1.527961	6.942332
1804	C	-1.423288	4.487149	4.336164
1805	H	-1.207535	3.971372	5.275633
1806	H	-0.555100	5.075236	4.042154
1807	H	-2.278363	5.153590	4.483569
1808	O	-2.014313	0.160905	-1.683927
1809	O	-3.402643	1.033868	-2.251371
1810	H	-2.917381	1.545823	-2.948495
1811	H	-1.626003	0.068989	-2.596578

Optimized Coordinates of ^{1}IC1 for Cu(I)-*Sm*AA10A·chitin+H_2O_2

N	2.002820	0.003311	-0.162415
C	2.795871	1.256672	0.002065
C	2.024350	2.212069	0.899285
C	0.775340	2.709680	0.260831
C	0.375802	4.010216	0.140449
N	-0.217421	1.908463	-0.251659
C	-1.198885	2.698691	-0.666842
N	-0.867912	3.975953	-0.443606
H	1.774159	1.705421	1.836395
H	2.660864	3.059862	1.154113
H	0.855032	4.929789	0.425744
H	-2.116038	2.360072	-1.123634
H	-1.444641	4.773524	-0.664309
H	2.952756	1.691244	-0.981109
H	3.768706	1.023250	0.439582
H	2.092153	-0.551301	0.684663
H	2.408882	-0.557788	-0.905890
C	1.073718	-6.417649	0.596464
C	0.681652	-5.490535	-0.566061
C	0.205526	-4.128654	-0.221184
C	0.574997	-2.928423	-0.758906
N	-0.794415	-3.836213	0.677304
C	-1.006216	-2.508677	0.658805
N	-0.188410	-1.928251	-0.203737
H	1.543525	-5.348323	-1.221214
H	-0.075970	-5.988935	-1.181012
H	-1.257036	-4.501103	1.307332
H	1.320567	-2.730727	-1.510175
H	-1.724312	-2.013974	1.288312
H	1.453267	-5.819456	1.428090
H	1.852195	-7.111531	0.281077
H	0.219423	-6.982963	0.967451
C	1.171235	3.589503	-7.221578
C	0.114687	3.347650	-6.119520
C	-0.192588	1.868777	-5.885827
C	-1.150247	1.517797	-4.729165
O	-1.810898	2.437647	-4.175499
O	-1.223970	0.297247	-4.414728
H	0.446526	3.805288	-5.185906
H	-0.816196	3.855235	-6.388618
H	-0.637386	1.445125	-6.794668
H	0.728827	1.308833	-5.719831
H	1.087984	2.810549	-7.984013
H	2.171860	3.508844	-6.789839
H	1.070231	4.571344	-7.684678
C	1.824687	-1.505959	4.125809
C	0.644527	-0.922135	3.355508
H	0.376358	-1.553478	2.510705
H	-0.234786	-0.842381	3.991390
H	0.868196	0.071774	2.965723
H	2.189089	-0.771277	4.847047
H	2.654198	-1.739326	3.453995

1866	H	1.526452	-2.425203	4.632417
1867	C	4.752390	0.813835	-6.894455
1868	C	5.069734	0.702102	-5.391584
1869	C	3.906037	1.115346	-4.538140
1870	C	2.964377	0.184545	-4.102250
1871	C	3.705057	2.462865	-4.233271
1872	C	1.833639	0.593888	-3.409564
1873	C	2.578524	2.873623	-3.532737
1874	C	1.633808	1.939455	-3.130678
1875	H	5.369829	-0.321318	-5.153621
1876	H	5.923929	1.345609	-5.164416
1877	H	3.109266	-0.865999	-4.328730
1878	H	4.435069	3.195277	-4.559631
1879	H	1.088890	-0.133705	-3.118880
1880	H	2.433076	3.923319	-3.308331
1881	H	0.742481	2.257562	-2.613866
1882	H	4.219434	1.747029	-7.085699
1883	H	4.106594	-0.007103	-7.211739
1884	H	5.683477	0.798278	-7.462765
1885	Cu	-0.103406	-0.002860	-0.550401
1886	C	-6.726829	-6.605182	1.277707
1887	O	-5.468003	-5.983264	1.546442
1888	H	-7.251433	-6.100051	0.464262
1889	H	-6.511102	-7.631919	0.989495
1890	H	-7.354777	-6.600540	2.173986
1891	C	-2.157717	-6.685820	4.693168
1892	C	-5.565552	-4.697798	2.051847
1893	C	-4.193591	-4.276997	2.587559
1894	C	-2.520832	-5.809147	3.523789
1895	N	-3.690819	-5.154486	3.612580
1896	O	-1.767853	-5.716539	2.545729
1897	C	-4.279076	-2.810670	3.073991
1898	O	-2.980297	-2.402871	3.472799
1899	C	-4.824078	-1.931596	1.945626
1900	O	-5.131526	-0.598497	2.403380
1901	C	-6.142612	-2.471614	1.394136
1902	O	-5.945614	-3.830189	0.992921
1903	C	-6.666566	-1.699081	0.204975
1904	O	-5.640952	-1.576349	-0.778133
1905	H	-6.329434	-4.648728	2.845320
1906	H	-3.503833	-4.334978	1.748221
1907	H	-4.971501	-2.757172	3.924343
1908	H	-4.089726	-1.873209	1.140725
1909	H	-6.903199	-2.428112	2.184806
1910	H	-1.197881	-6.354731	5.092269
1911	H	-2.033965	-7.709586	4.336638
1912	H	-4.240025	-5.271482	4.450552
1913	H	-2.903948	-6.668487	5.485890
1914	H	-3.035567	-1.478192	3.775287
1915	H	-7.531391	-2.236219	-0.197337
1916	H	-6.998790	-0.718712	0.550019
1917	H	-5.982200	-1.051881	-1.511929
1918	C	-6.817891	1.944048	-1.126031
1919	C	-4.113189	0.343884	2.363754

1920	C	-4.695361	1.725182	2.022178
1921	C	-6.501102	1.927038	0.350528
1922	N	-5.206728	1.718253	0.664094
1923	O	-7.382923	2.092446	1.197029
1924	C	-3.646801	2.831706	2.163586
1925	O	-4.279651	4.088315	1.993537
1926	C	-2.819945	2.738300	3.435240
1927	O	-1.678125	3.573311	3.269396
1928	C	-2.367444	1.296789	3.708647
1929	O	-3.482438	0.390607	3.644958
1930	C	-1.731620	1.141486	5.073911
1931	O	-2.603135	1.667532	6.072930
1932	H	-3.357681	0.071923	1.613259
1933	H	-5.534790	1.913850	2.691658
1934	H	-2.918851	2.685614	1.352833
1935	H	-3.421636	3.086126	4.278731
1936	H	-1.630466	1.012299	2.948625
1937	H	-7.248820	2.914618	-1.378000
1938	H	-7.574571	1.184605	-1.330984
1939	H	-4.543352	1.612989	-0.097367
1940	H	-5.943023	1.762949	-1.748988
1941	H	-3.587880	4.754852	1.892016
1942	H	-1.541341	0.081386	5.257398
1943	H	-0.771701	1.666211	5.071490
1944	H	-2.184813	1.540038	6.932693
1945	C	-1.445306	4.502108	4.324480
1946	H	-1.239998	3.995298	5.271217
1947	H	-0.574733	5.089244	4.035568
1948	H	-2.303214	5.168354	4.455433
1949	O	-1.782843	-0.052829	-1.542454
1950	O	-3.541914	1.306702	-2.093463
1951	H	-2.961772	1.626921	-2.824796
1952	H	-1.573817	0.039080	-2.495869

Optimized Coordinates of ^{3}IC1 for Cu(I)-*Sm*AA10A·chitin+H_2O_2

N	2.001145	0.007932	-0.167947
C	2.795049	1.259203	0.001343
C	2.023556	2.214552	0.898656
C	0.774069	2.712173	0.260185
C	0.373522	4.012502	0.145696
N	-0.220387	1.912080	-0.255924
C	-1.201553	2.704809	-0.665614
N	-0.870776	3.980871	-0.436976
H	1.773537	1.707894	1.835813
H	2.659989	3.062361	1.153369
H	0.852879	4.930701	0.435101
H	-2.122388	2.366677	-1.118236
H	-1.448212	4.779288	-0.652710
H	2.954974	1.695629	-0.980671
H	3.766455	1.022954	0.440482
H	2.086315	-0.548790	0.678050
H	2.407794	-0.551980	-0.912012
C	1.073696	-6.415261	0.596122
C	0.681541	-5.488577	-0.565903
C	0.204640	-4.130475	-0.207827
C	0.583557	-2.922728	-0.720527
N	-0.795984	-3.848435	0.693138
C	-0.996982	-2.518153	0.700132
N	-0.172510	-1.927158	-0.147264
H	1.543585	-5.341611	-1.219665
H	-0.075523	-5.985895	-1.182266
H	-1.263043	-4.521575	1.310627
H	1.332412	-2.717143	-1.466476
H	-1.714301	-2.031773	1.337207
H	1.453298	-5.817023	1.427678
H	1.852645	-7.109228	0.281686
H	0.219552	-6.980681	0.967402
C	1.169228	3.591772	-7.222116
C	0.113597	3.350012	-6.120693
C	-0.202449	1.872979	-5.897970
C	-1.158946	1.522472	-4.742695
O	-1.815451	2.437193	-4.179087
O	-1.233002	0.298637	-4.436315
H	0.449431	3.798932	-5.184355
H	-0.814498	3.865420	-6.384738
H	-0.651455	1.458323	-6.808853
H	0.715276	1.305578	-5.736570
H	1.085945	2.812796	-7.984528
H	2.169950	3.510402	-6.790629
H	1.069268	4.573656	-7.685329
C	1.824665	-1.503439	4.125269
C	0.644651	-0.919883	3.355313
H	0.379021	-1.549341	2.509206
H	-0.235434	-0.842552	3.990505
H	0.867439	0.075254	2.968009
H	2.189107	-0.768690	4.846467
H	2.654318	-1.736590	3.453529

2007	H	1.526750	-2.422855	4.631794
2008	C	4.750673	0.816435	-6.895428
2009	C	5.068225	0.704759	-5.392628
2010	C	3.903677	1.116677	-4.539987
2011	C	2.959168	0.185748	-4.110900
2012	C	3.704194	2.463323	-4.230256
2013	C	1.826751	0.594243	-3.420475
2014	C	2.576014	2.873235	-3.531968
2015	C	1.628201	1.939071	-3.137110
2016	H	5.369244	-0.318460	-5.154908
2017	H	5.921695	1.349100	-5.165180
2018	H	3.103071	-0.864126	-4.341074
2019	H	4.436629	3.195713	-4.551167
2020	H	1.080163	-0.133370	-3.134967
2021	H	2.431506	3.922243	-3.303772
2022	H	0.735675	2.257009	-2.622218
2023	H	4.217605	1.749580	-7.086617
2024	H	4.104909	-0.004567	-7.212612
2025	H	5.681697	0.800915	-7.463860
2026	Cu	-0.099348	0.004254	-0.561553
2027	C	-6.726752	-6.603944	1.279196
2028	O	-5.467957	-5.981335	1.546685
2029	H	-7.252206	-6.099261	0.466020
2030	H	-6.510764	-7.630661	0.991100
2031	H	-7.353876	-6.599350	2.176044
2032	C	-2.170227	-6.700314	4.687944
2033	C	-5.565730	-4.695981	2.052294
2034	C	-4.193607	-4.275015	2.587867
2035	C	-2.530859	-5.824267	3.517274
2036	N	-3.690577	-5.152525	3.612932
2037	O	-1.784391	-5.746258	2.533060
2038	C	-4.279043	-2.808835	3.074433
2039	O	-2.980765	-2.400769	3.474586
2040	C	-4.824378	-1.929716	1.946042
2041	O	-5.131816	-0.596624	2.403838
2042	C	-6.143631	-2.469949	1.396106
2043	O	-5.946477	-3.828304	0.993903
2044	C	-6.671259	-1.697995	0.208310
2045	O	-5.649277	-1.576888	-0.778797
2046	H	-6.329157	-4.647349	2.846247
2047	H	-3.504067	-4.332868	1.748334
2048	H	-4.971982	-2.755518	3.924416
2049	H	-4.090445	-1.871161	1.140720
2050	H	-6.902715	-2.427429	2.188298
2051	H	-1.206989	-6.374436	5.083262
2052	H	-2.054295	-7.725786	4.333852
2053	H	-4.235361	-5.261142	4.454951
2054	H	-2.913717	-6.675773	5.483046
2055	H	-3.036635	-1.476018	3.776915
2056	H	-7.537791	-2.235251	-0.190245
2057	H	-7.001797	-0.717143	0.553560
2058	H	-5.991501	-1.048775	-1.509494
2059	C	-6.809572	1.952178	-1.130457
2060	C	-4.113543	0.345813	2.363996

2061	C	-4.695884	1.727115	2.022502
2062	C	-6.498223	1.926565	0.347251
2063	N	-5.204249	1.721012	0.663428
2064	O	-7.384303	2.081526	1.191399
2065	C	-3.647478	2.833673	2.163735
2066	O	-4.280478	4.090230	1.993743
2067	C	-2.820471	2.740313	3.435229
2068	O	-1.678737	3.575544	3.269599
2069	C	-2.367610	1.298866	3.708352
2070	O	-3.482539	0.392592	3.645082
2071	C	-1.731283	1.143787	5.073336
2072	O	-2.602633	1.669884	6.072554
2073	H	-3.358321	0.073964	1.613222
2074	H	-5.535952	1.915488	2.691281
2075	H	-2.919676	2.687541	1.352938
2076	H	-3.422166	3.087868	4.278825
2077	H	-1.630934	1.014478	2.947985
2078	H	-7.242262	2.922932	-1.378523
2079	H	-7.562745	1.191447	-1.343415
2080	H	-4.536673	1.633010	-0.097242
2081	H	-5.931621	1.777030	-1.750933
2082	H	-3.588729	4.756592	1.890921
2083	H	-1.540759	0.083807	5.256969
2084	H	-0.771450	1.668739	5.070715
2085	H	-2.188942	1.532546	6.933046
2086	C	-1.447887	4.506017	4.323736
2087	H	-1.243658	4.000481	5.271388
2088	H	-0.577243	5.093181	4.035111
2089	H	-2.306398	5.171906	4.452463
2090	O	-1.693403	-0.173261	-1.671300
2091	O	-3.492239	1.270341	-2.000175
2092	H	-2.970222	1.593340	-2.769884
2093	H	-1.519607	0.063551	-2.609308

References

(1) Vaaje-Kolstad, G.; Houston, D. R.; Riemen, A. H. K.; Eijsink, V. G. H.; Van Aalten, D. M. F. Crystal Structure and Binding Properties of the Serratia Marcescens Chitin-Binding Protein CBP21. *J. Biol. Chem.* **2005**, *280* (12), 11313–11319. https://doi.org/10.1074/jbc.M407175200.

(2) Manoil, C.; Beckwith, J. A Genetic Approach to Analyzing Membrane Protein Topology. *Science* **1986**, *233* (4771), 1403–1408. https://doi.org/10.1126/science.3529391.

(3) Frandsen, K. E. H.; Simmons, T. J.; Dupree, P.; Poulsen, J.-C. N.; Hemsworth, G. R.; Ciano, L.; Johnston, E. M.; Tovborg, M.; Johansen, K. S.; Von Freiesleben, P.; Marmuse, L.; Fort, S.; Cottaz, S.; Driguez, H.; Henrissat, B.; Lenfant, N.; Tuna, F.; Baldansuren, A.; Davies, G. J.; Lo Leggio, L.; Walton, P. H. The Molecular Basis of Polysaccharide Cleavage by Lytic Polysaccharide Monooxygenases. *Nat. Chem. Biol.* **2016**, *12* (4), 298–303. https://doi.org/10.1038/nchembio.2029.

(4) Lindley, P. J.; Parkin, A.; Davies, G. J.; Walton, P. H. Mapping the Protonation States of the Histidine Brace in an AA10 Lytic Polysaccharide Monooxygenase Using CW-EPR Spectroscopy and DFT Calculations. *Faraday Discuss.* **2022**, *234* (0), 336–348. https://doi.org/10.1039/D1FD00068C.

(5) Gómez-Piñeiro, R. J.; Drosou, M.; Bertaina, S.; Decroos, C.; Simaan, A. J.; Pantazis, D. A.; Orio, M. Decoding the Ambiguous Electron Paramagnetic Resonance Signals in the Lytic Polysaccharide Monooxygenase from *Photorhabdus Luminescens*. *Inorg. Chem.* **2022**, *61* (20), 8022–8035. https://doi.org/10.1021/acs.inorgchem.2c00766.

(6) Haak, J.; Golten, O.; Sørlie, M.; Eijsink, V. G. H.; Cutsail, G. E. pH-Mediated Manipulation of the Histidine Brace in LPMOs and Generation of a Tri-Anionic Variant, Investigated by EPR, ENDOR, ESEEM and HYSCORE Spectroscopy. *Chem. Sci.* **2024**, *16* (1), 233–254. https://doi.org/10.1039/D4SC04794J.

(7) Hall, K. R.; Joseph, C.; Ayuso-Fernández, I.; Tamhankar, A.; Rieder, L.; Skaali, R.; Golten, O.; Neese, F.; Røhr, Å. K.; Jannuzzi, S. A. V.; DeBeer, S.; Eijsink, V. G. H.; Sørlie, M. A Conserved Second Sphere Residue Tunes Copper Site Reactivity in Lytic Polysaccharide Monooxygenases. *J. Am. Chem. Soc.* **2023**, *145* (34), 18888–18903. https://doi.org/10.1021/jacs.3c05342.

(8) Bissaro, B.; Isaksen, I.; Vaaje-Kolstad, G.; Eijsink, V. G. H.; Røhr, Å. K. How a Lytic Polysaccharide Monooxygenase Binds Crystalline Chitin. *Biochemistry* **2018**, *57* (12), 1893–1906. https://doi.org/10.1021/acs.biochem.8b00138.

(9) Stoll, S.; Schweiger, A. EasySpin, a Comprehensive Software Package for Spectral Simulation and Analysis in EPR. *J. Magn. Reson.* **2006**, *178* (1), 42–55. https://doi.org/10.1016/j.jmr.2005.08.013.

(10) The PyMOL Molecular Graphics System, Version 2.5.4 Schrödinger, LLC.

(11) Neese, F. The ORCA Program System. *WIREs Comput. Mol. Sci.* **2012**, *2* (1), 73–78. https://doi.org/10.1002/wcms.81.

(12) Neese, F.; Wennmohs, F.; Becker, U.; Riplinger, C. The ORCA Quantum Chemistry Program Package. *J. Chem. Phys.* **2020**, *152* (22), 224108. https://doi.org/10.1063/5.0004608.

(13) Neese, F. Software Update: The ORCA Program System—Version 5.0. *WIREs Comput. Mol. Sci.* **2022**, *12* (5), e1606. https://doi.org/10.1002/wcms.1606.

(14) Lehtola, S.; Steigemann, C.; Oliveira, M. J. T.; Marques, M. A. L. Recent Developments in Libxc — A Comprehensive Library of Functionals for Density Functional Theory. *SoftwareX* **2018**, *7*, 1–5. https://doi.org/10.1016/j.softx.2017.11.002.

(15) Ekström, U.; Visscher, L.; Bast, R.; Thorvaldsen, A. J.; Ruud, K. Arbitrary-Order Density Functional Response Theory from Automatic Differentiation. *J. Chem. Theory Comput.* **2010**, *6* (7), 1971–1980. https://doi.org/10.1021/ct100117s.

(16) Liu, Z.; Lu, T.; Chen, Q. An Sp-Hybridized All-Carboatomic Ring, Cyclo[18]Carbon: Electronic Structure, Electronic Spectrum, and Optical Nonlinearity. *Carbon* **2020**, *165*, 461–467. https://doi.org/10.1016/j.carbon.2020.05.023.

(17) Lu, T.; Chen, F. Multiwfn: A Multifunctional Wavefunction Analyzer. *J. Comput. Chem.* **2012**, *33* (5), 580–592. https://doi.org/10.1002/jcc.22885.

(18) Bissaro, B.; Streit, B.; Isaksen, I.; Eijsink, V. G. H.; Beckham, G. T.; DuBois, J. L.; Røhr, Å. K. Molecular Mechanism of the Chitinolytic Peroxygenase Reaction. *Proc. Natl. Acad. Sci.* **2020**, *117* (3), 1504–1513. https://doi.org/10.1073/pnas.1904889117.

(19) Williams, C. J.; Headd, J. J.; Moriarty, N. W.; Prisant, M. G.; Videau, L. L.; Deis, L. N.; Verma, V.; Keedy, D. A.; Hintze, B. J.; Chen, V. B.; Jain, S.; Lewis, S. M.; Arendall, W. B.; Snoeyink, J.; Adams, P. D.; Lovell, S. C.; Richardson, J. S.; Richardson, D. C. MolProbity: More and Better Reference Data for Improved All-atom Structure Validation. *Protein Sci.* **2018**, *27* (1), 293–315. https://doi.org/10.1002/pro.3330.

(20) Olsson, M. H. M.; Søndergaard, C. R.; Rostkowski, M.; Jensen, J. H. PROPKA3: Consistent Treatment of Internal and Surface Residues in Empirical p K_a Predictions. *J. Chem. Theory Comput.* **2011**, *7* (2), 525–537. https://doi.org/10.1021/ct100578z.

(21) Becke, A. D. Density-Functional Exchange-Energy Approximation with Correct Asymptotic Behavior. *Phys. Rev. A* **1988**, *38* (6), 3098–3100. https://doi.org/10.1103/PhysRevA.38.3098.

(22) Perdew, J. P. Density-Functional Approximation for the Correlation Energy of the Inhomogeneous Electron Gas. *Phys. Rev. B* **1986**, *33* (12), 8822–8824. https://doi.org/10.1103/PhysRevB.33.8822.

(23) Weigend, F.; Ahlrichs, R. Balanced Basis Sets of Split Valence, Triple Zeta Valence and Quadruple Zeta Valence Quality for H to Rn: Design and Assessment of Accuracy. *Phys. Chem. Chem. Phys.* **2005**, *7* (18), 3297. https://doi.org/10.1039/b508541a.

(24) Weigend, F. Accurate Coulomb-Fitting Basis Sets for H to Rn. *Phys. Chem. Chem. Phys.* **2006**, *8* (9), 1057. https://doi.org/10.1039/b515623h.

(25) Helmich-Paris, B.; De Souza, B.; Neese, F.; Izsák, R. An Improved Chain of Spheres for Exchange Algorithm. *J. Chem. Phys.* **2021**, *155* (10), 104109. https://doi.org/10.1063/5.0058766.

(26) Barone, V.; Cossi, M. Quantum Calculation of Molecular Energies and Energy Gradients in Solution by a Conductor Solvent Model. *J. Phys. Chem. A* **1998**, *102* (11), 1995–2001. https://doi.org/10.1021/jp9716997.

(27) Garcia-Ratés, M.; Neese, F. Effect of the Solute Cavity on the Solvation Energy and Its Derivatives within the Framework of the Gaussian Charge Scheme. *J. Comput. Chem.* **2020**, *41* (9), 922–939. https://doi.org/10.1002/jcc.26139.

(28) Caldeweyher, E.; Ehlert, S.; Hansen, A.; Neugebauer, H.; Spicher, S.; Bannwarth, C.; Grimme, S. A Generally Applicable Atomic-Charge Dependent London Dispersion Correction. *J. Chem. Phys.* **2019**, *150* (15), 154122. https://doi.org/10.1063/1.5090222.

(29) Caldeweyher, E.; Bannwarth, C.; Grimme, S. Extension of the D3 Dispersion Coefficient Model. *J. Chem. Phys.* **2017**, *147* (3), 034112. https://doi.org/10.1063/1.4993215.

(30) Tandrup, T.; Muderspach, S. J.; Banerjee, S.; Santoni, G.; Ipsen, J. Ø.; Hernández-Rollán, C.; Nørholm, M. H. H.; Johansen, K. S.; Meilleur, F.; Lo Leggio, L. Changes in Active-Site Geometry on X-Ray Photoreduction of a Lytic Polysaccharide Monooxygenase Active-Site Copper and Saccharide Binding. *IUCrJ* **2022**, *9* (Pt 5), 666–681. https://doi.org/10.1107/S2052252522007175.

(31) Vu, V. V.; Ngo, S. T. Copper Active Site in Polysaccharide Monooxygenases. *Coord. Chem. Rev.* **2018**, *368*, 134–157. https://doi.org/10.1016/j.ccr.2018.04.005.

(32) Kas, J. J.; Vila, F. D.; Pemmaraju, C. D.; Tan, T. S.; Rehr, J. J. Advanced Calculations of X-Ray Spectroscopies with FEFF10 and Corvus. *J. Synchrotron Radiat.* **2021**, *28* (6), 1801–1810. https://doi.org/10.1107/S1600577521008614.

(33) Beckwith, M. A.; Ames, W.; Vila, F. D.; Krewald, V.; Pantazis, D. A.; Mantel, C.; Pécaut, J.; Gennari, M.; Duboc, C.; Collomb, M.-N.; Yano, J.; Rehr, J. J.; Neese, F.; DeBeer, S. How Accurately Can Extended X-Ray Absorption Spectra Be Predicted from First Principles? Implications for Modeling the Oxygen-Evolving Complex in Photosystem II. *J. Am. Chem. Soc.* **2015**, *137* (40), 12815–12834. https://doi.org/10.1021/jacs.5b00783.

(34) Kjaergaard, C. H.; Qayyum, M. F.; Wong, S. D.; Xu, F.; Hemsworth, G. R.; Walton, D. J.; Young, N. A.; Davies, G. J.; Walton, P. H.; Johansen, K. S.; Hodgson, K. O.; Hedman, B.; Solomon, E. I. Spectroscopic and Computational Insight into the Activation of O_2 by the Mononuclear Cu Center in Polysaccharide Monooxygenases. *Proc. Natl. Acad. Sci.* **2014**, *111* (24), 8797–8802. https://doi.org/10.1073/pnas.1408115111.

(35) Munzone, A.; Pujol, M.; Tamhankar, A.; Joseph, C.; Mazurenko, I.; Réglier, M.; Jannuzzi, S. A. V.; Royant, A.; Sicoli, G.; DeBeer, S.; Orio, M.; Simaan, A. J.; Decroos, C. Integrated Experimental and Theoretical Investigation of Copper Active Site Properties of a Lytic Polysaccharide Monooxygenase from Serratia Marcescens. *Inorg. Chem.* **2024**, *63* (24), 11063–11078. https://doi.org/10.1021/acs.inorgchem.4c00602.

(36) Glatzel, P.; Jacquamet, L.; Bergmann, U.; de Groot, F. M. F.; Cramer, S. P. Site-Selective EXAFS in Mixed-Valence Compounds Using High-Resolution Fluorescence Detection: A Study of Iron in Prussian Blue. *Inorg. Chem.* **2002**, *41* (12), 3121–3127. https://doi.org/10.1021/ic010709m.

(37) Ravel, B.; Newville, M. *ATHENA* , *ARTEMIS* , *HEPHAESTUS* : Data Analysis for X-Ray Absorption Spectroscopy Using *IFEFFIT*. *J. Synchrotron Radiat.* **2005**, *12* (4), 537–541. https://doi.org/10.1107/S0909049505012719.

(38) Lim, H.; Brueggemeyer, M. T.; Transue, W. J.; Meier, K. K.; Jones, S. M.; Kroll, T.; Sokaras, D.; Kelemen, B.; Hedman, B.; Hodgson, K. O.; Solomon, E. I. Kβ X-Ray Emission Spectroscopy of Cu(I)-Lytic Polysaccharide Monooxygenase: Direct Observation of the Frontier Molecular Orbital for H2O2 Activation. *J. Am. Chem. Soc.* **2023**, *145* (29), 16015–16025. https://doi.org/10.1021/jacs.3c04048.
